# Mild deprotection of the *N-tert*-butyloxycarbonyl (*N*-Boc) group using oxalyl chloride†

**DOI:** 10.1039/d0ra04110f

**Published:** 2020-06-23

**Authors:** Nathaniel George, Samuel Ofori, Sean Parkin, Samuel G. Awuah

**Affiliations:** Department of Chemistry, University of Kentucky Lexington Kentucky 40506 USA awuah@uky.edu

## Abstract

We report a mild method for the selective deprotection of the *N*-Boc group from a structurally diverse set of compounds, encompassing aliphatic, aromatic, and heterocyclic substrates by using oxalyl chloride in methanol. The reactions take place under room temperature conditions for 1–4 h with yields up to 90%. This mild procedure was applied to a hybrid, medicinally active compound FC1, which is a novel dual inhibitor of IDO1 and DNA Pol gamma. A broader mechanism involving the electrophilic character of oxalyl chloride is postulated for this deprotection strategy.

## Abstract 2

We report a mild method for the selective deprotection of the *N*-Boc group from a structurally diverse set of compounds, encompassing aliphatic, aromatic, and heterocyclic substrates by using oxalyl chloride in methanol.

## Graphical abstract

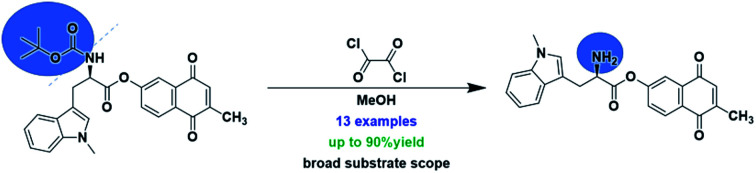

## Introduction

Synthetic organic transformations require the appropriate selection of reagents, catalysts, and most importantly, temporal masking and demasking agents. The objective for the deployment of relevant masking–demasking agents is to selectively form bonds of interest, whilst minimizing competing reactions with reactive functional groups. A good protecting group will selectively block the functional group of interest, will be stable to the projected reactions, and can be removed with readily available de-masking agents.^1^

The amino group is a key functionality that is present in several compounds: natural products, amino acids and peptides.^2^ As such, there is an emergent need for its masking and demasking in forward synthesis. The *tert*-butyloxycarbonyl (Boc) group is one of the classical masking functionalities employed in organic synthesis for the protection of amino groups.^3–5^ Boc fulfills this requirement of a ‘good’ protecting group, and is preferred in amino protection because of its stability to nucleophilic reagents, hydrogenolysis and base hydrolysis.^6,7^ The Boc-masking group is installed by reacting the amino-substrate with di-*tert*-butyl dicarbonate under basic conditions,^8–10^ or solvent free conditions.^11–13^

Traditional approaches for *N*-Boc deprotection relies largely on TFA-induced cleavage.^14^ Other strategies reported for the deprotection of *N*-Boc include the use of metal catalysts,^15,16^ as well as acetylchloride in methanol,^17^*N*-Boc removal with HCl in organic solvents: ethylacetate,^18^ dioxane,^19^ in acetone.^20^ Other *N*-Boc deprotection methodologies include aqueous phosphoric acid,^21,22^ conc. sulfuric acid in *tert* butylacetate,^3^ boiling water;^23^ silica gel has also been reported to effect the deprotection of *N*-Boc from thermally-sensitive heterocycles including heterocondensed pyrroles.^24^ Solvent free *N*-Boc deprotection strategies have been reported; Pal *et al.* reported the deprotection of several structurally diverse *N*-Boc substrates by using catalytic amounts of iodine.^25^ Aouf and co-workers have also reported the selective cleavage of *N*-Boc from *N*-Boc chiral cyclosulfamides by fusion: mixing *N*-Boc substrates with catalytic amounts of iodine under reduced pressure.^26^ Guillaumet and co-workers have developed the basic deprotection of *N*-Boc substrates – using sodium carbonate in refluxing DME.^27^ Similar basic *N*-Boc deprotection has been reported by Ewing *et al.*, where sodium *t*-butoxide in slightly wet tetrahydrofuran was used to cleave off unactivated primary *N*-Boc from base stable substrates.^28^ Microwave assisted basic deprotection of secondary *N*-Boc substrates have been reported by Williams & Dandepally.^29^ Jia and co-workers have also developed a catalyst-free water-based deprotection of *N*-Boc aliphatic and aromatic substrates.^30^

Most recently, several *N*-Boc deprotection schemes have been reported. These include *N*-Boc deprotection *via* thermolysis^31,32^ and TMSI-mediated deprotection of *N*-Boc in zwitterionic compounds.^33^

In most cases, small molecules with sensitive functional groups or unique scaffolds are not compatible with these harsh deprotection conditions. Therefore, alternative reagents for deprotection, while providing functional group tolerance will be quintessential in the masking and unmasking of amines – a paradigm for broad utility.

Oxalyl chloride is a highly accessible organic reagent that has many applications: from the routine synthesis of acid chlorides to the preparation of dihydroquinolines *via* a modified Bischler–Napieralski ring closure.^34^ The reactivity of oxalyl chloride with amides manifests in useful products through a typical imidoyl chloride intermediate when acetamide starting materials are used.^35^

## Results and discussion

In our effort to generate an acylchloride from the C-terminus of a *N*-Boc protected peptidomimetic using oxalyl chloride, we observed the concomitant formation of the deprotected *N*-Boc to form the peptidomimetic with a free amine. We therefore set out to investigate if oxalyl chloride can mildly promote the deprotection of *N*-Boc substrates. Using 1-napthylamine as a model compound, we screened oxalyl chloride with a host of organic solvents at varying temperature conditions and equivalence. We found that the deprotection reaction proceeds poorly in neat oxalyl chloride. In addition, the reaction proceeds to access deprotected amino substrates in CHCl_3_ in moderate yields at room temperature over 24–48 h. However, under refluxing conditions in CHCl_3_, *N*-chloroalkyl products formed, which were detected by GC-MS even after aqueous work-up.

Further optimization led to the identification of a reaction condition that involved the use of five (5) equivalents of oxalyl chloride in methanol, which rapidly deprotects *N*-Boc substrates with respectable yields (Table 1). Ultimately, the use of three (3) equivalents of oxalyl chloride in MeOH achieved good-to-excellent yields of deprotected *tert*-butyl carbamates. Here, we report a mild, and selective deprotection of *tert*-butyl carbamates using oxalyl chloride in methanol. The approach is tolerant to several functional groups.

**Table 1:** Optimization of deprotection using *N*-Boc-1-naphthylamine-amine^a^

Entry	Reaction conditions	Time (h)	Yield (%)^a^
1	Oxalyl chloride, neat, RT	72	0
2	Oxalyl chloride, CHCl_3_	24	23^b^
3	Oxalyl chloride, CHCl_3_, 50 °C	24	12
4	Oxalyl chloride, CHCl_3_, 62 °C	24	0
5	Oxalyl chloride, MeOH, RT	0.5	80^b^

a Conditions: (a) (COCl)_2_ (1–3 equiv.), (b) (COCl)_2_ (3 equiv.).

**Scheme 1:**
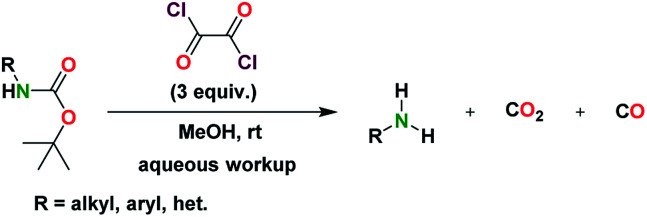
General deprotection reaction scheme.

The general deprotection scheme is shown in Scheme 1. We then applied this deprotection strategy to a variety of aromatic, aliphatic, and heterocyclic substrates.

Table 2 illustrates the wide substrate scope of the oxalyl chloride–methanol deprotection strategy. It was effective against structurally diverse *N*-Boc amines; from aromatics, heterocyclic, aliphatics to alicyclic systems.

**Table 2:** Deprotection of structurally diverse *N*-Boc-amines

Entry^a^	Substrate (a)	Time/h	Product (b)	Yield (%)
1	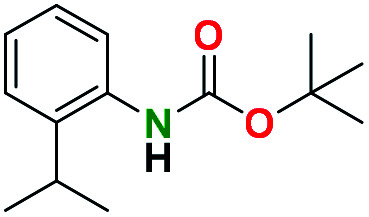	2	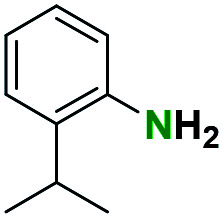	70
2	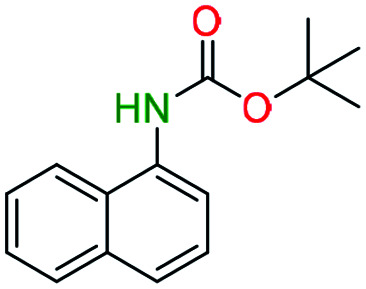	1.5	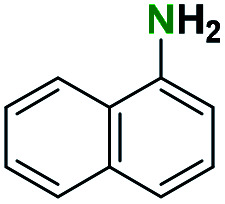	87
3	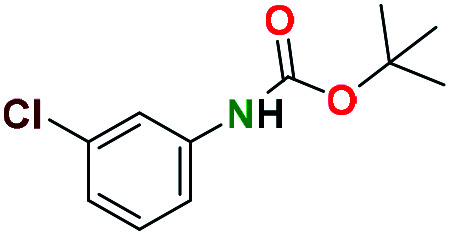	1.5	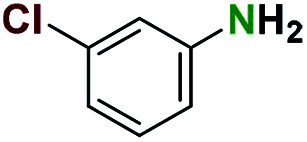	83
4	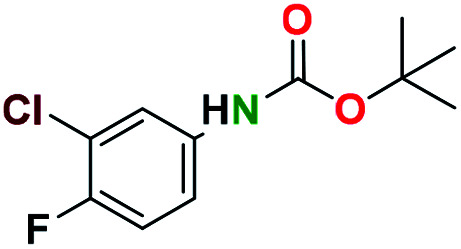	1	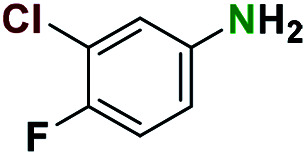	80
5	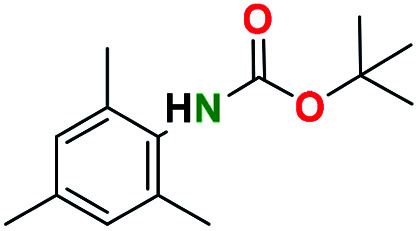	2	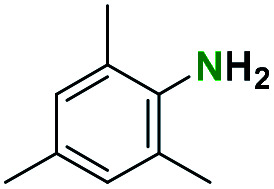	83
6	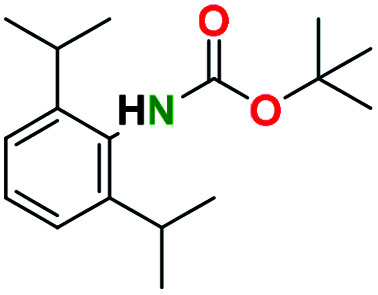	3	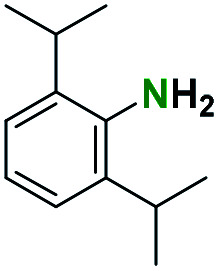	78
7	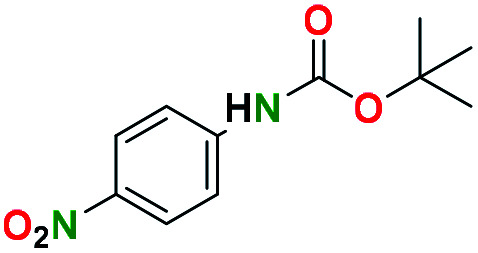	1.5	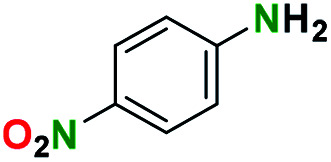	76
8	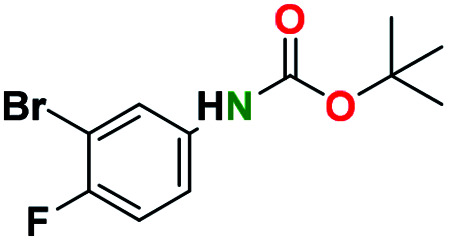	1.5	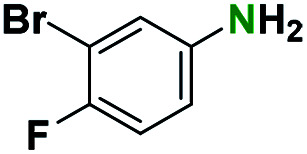	81
9	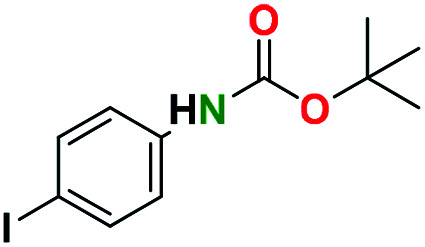	1	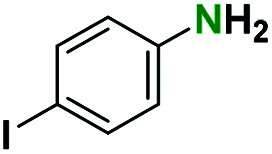	86
10	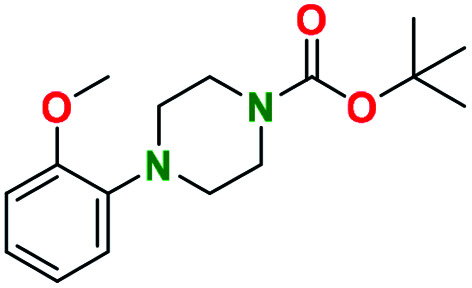	2.5	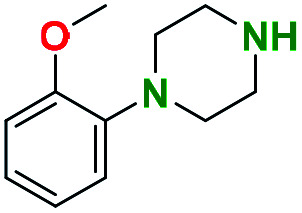	64
11	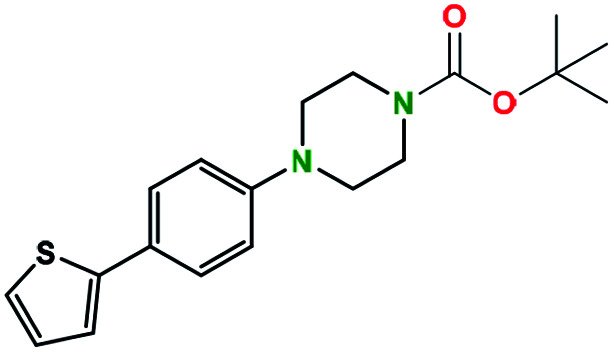	4	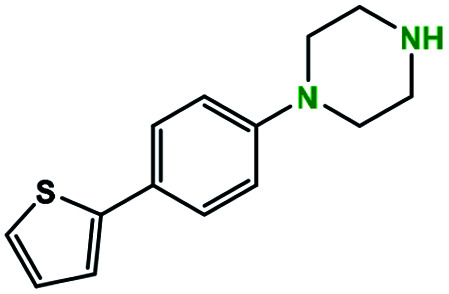	87
12	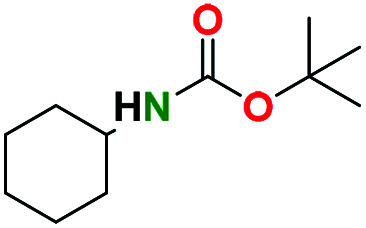	3.5	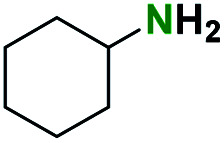	55
13	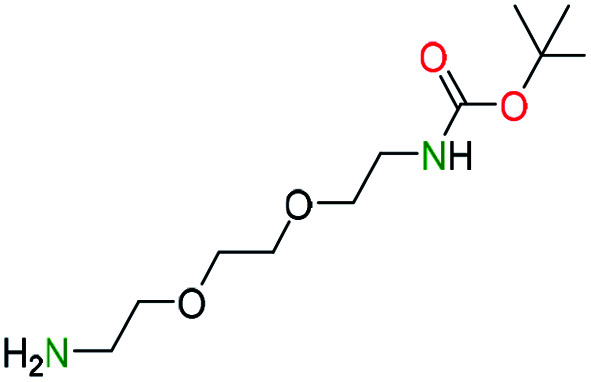	2	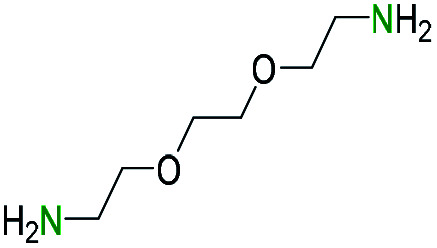	90

a All reactions were conducted in methanol at RT, with *N*-Boc substrate (1 equiv.), (COCl)_2_ (3 equiv.).

Generally, the deprotection of *N*-Boc directly linked to aromatic moieties (entries 1–9) were reasonably fast, occurring within 3 h and with high yields, >70%. Especially, compounds with electron withdrawing groups (EWG) including nitro, fluoro, chloro, iodo, or bromo display a faster response to the oxalyl chloride deprotection reagent with reactions in an hour.

Conceivably, electronic destabilization of the aryl carbamate induced by EWG promotes its cleavage by oxalyl chloride. We further observed that steric hindrance of methyl or isopropyl units attached to the aromatic ring and adjacent to the *N*-Boc group slows the reaction as seen for entries 2, 5, and 6. Moreover, the deprotection reaction of heteroaromatics in entry 11 proceeded modestly in 4 h. Taken together, favorable deprotection of aromatics by this deprotection strategy can be attributed to favorable electronic effects of these selected aromatic systems. The enhanced reactivity of the aromatic systems in contrast to their non-aromatic counterparts can be rationalized on the basis of the weakly nucleophilic oxygen atom of the carbonyl *N*-Boc atom. This oxygen is often stabilized or destabilized by the side group/chain directly connected to the *N*-Boc group. For entries possessing aromatics and electron-withdrawing groups, the pronounced ground-state destabilization of the carbonyl group caused by resonance or inductive effects, informs the increased O-atom reactivity to the electrophilic oxalyl chloride. This phenomenon could explain why the rate of reaction for alicyclic or heterocyclic systems were relatively slower.

Hybrid drugs possess multiple pharmacological activity.^36^ These agents often have sensitive functional groups and their synthesis require protection and deprotection steps that are mild and of broad tolerance. One such molecule of interest in our laboratory is 5, which is a dual inhibitor of indoleamine-2,3-dioxygenase 1 (IDO1) and DNA polymerase gamma.

**Scheme 2:**
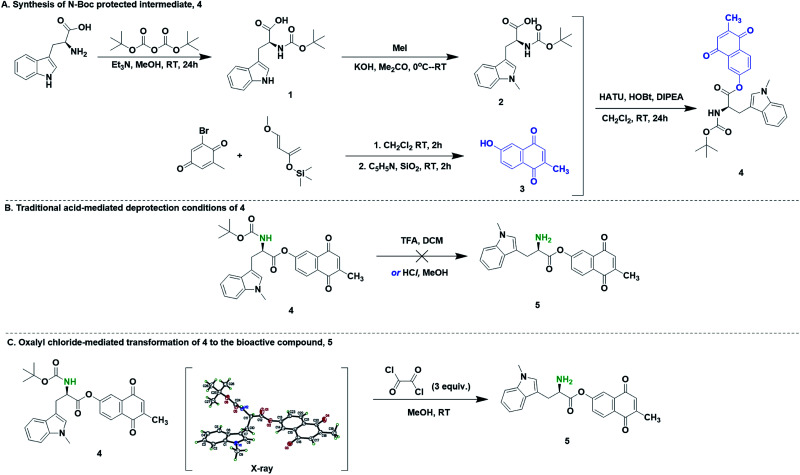
Synthesis of 5 (FC1) *via* oxalyl chloride-mediated deprotection of 4 (EC1). The X-ray of EC1 is drawn at 50% thermal ellipsoid. Solvent molecules were removed for clarity.

Hybrid drugs possess multiple pharmacological activity. These agents often have sensitive functional groups and their synthesis require protection and deprotection steps that are mild and of broad tolerance. One such molecule of interest in our laboratory is 5, which is a dual inhibitor of indoleamine-2,3-dioxygenase 1 (IDO1) and DNA polymerase gamma. To demonstrate the versatility of this deprotection strategy, we used the *N*-Boc protected small-molecule precursor of 5, which possess acid-labile functionality. The synthesis of 4 was accomplished through, first, *N*-Boc protection of d-tryptophan, 1 and the subsequent *N*-methylation of the indole nitrogen to yield compound 2. Second, hydroxymenadione, 3 was synthesized from a derivatized quinone and a Danishefsky diene. Third, amide coupling of d-methyl tryptophan and hydroxymenadione afforded 4 in 10% yield. Initial attempts to convert 4 to 5 using traditional acid-mediated protocols were unsuccessful. For example, experiments with (1–60%) TFA in DCM, and HCl in dioxane/methanol did not yield FC1. However, products corresponding to the cleavage of the ester bond in EC1 were observed. We envisaged that the facile functional group tolerance of the described oxalyl chloride/MeOH methodology may work for our compound, 4. We therefore applied our deprotection methodology to 5. We observed a clean transformation of 4 to 5 (Scheme 2). Overall, oxalyl chloride is a worthy *N*-Boc deprotection reagent for compounds with multiple functional groups and acid-labile groups.

### Determination of HCl effectiveness in deprotection

**Fig. 1:**
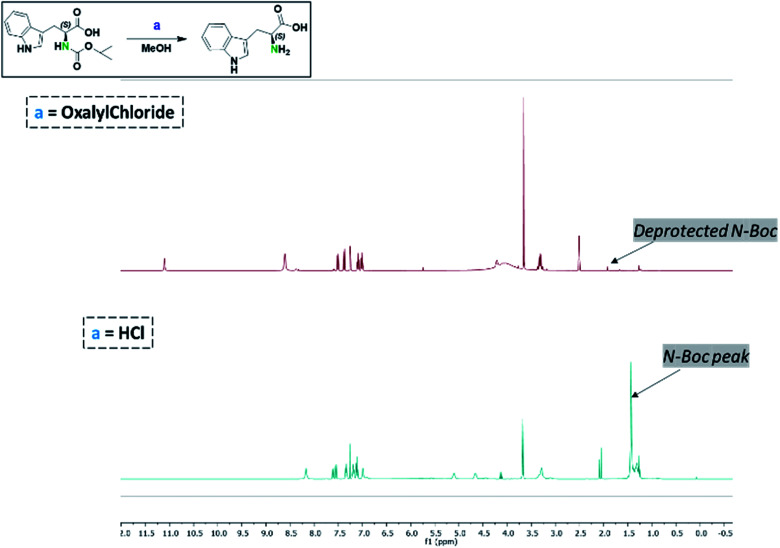
Comparative reaction conditions for the deprotection of *N*-Boc-l-tryptophan (methanol was used as the reaction solvent).

Considering that HCl/MeOH was incapable of deprotecting the *N*-Boc group in 4, we further explored the potential role of HCl to deepen our understanding of this reaction. One of the by-products of oxalyl chloride–alcohol reactions is the generation of HCl. Thus, the presumptive role of *in situ* generated HCl in the deprotection of *N*-Boc substrates is reasonable. To examine this assertion, an equivalent mole of HCl generated in the oxalyl chloride–MeOH system was utilized in a model reaction as shown in Fig. 1. Specifically, *N*-Boc-l-tryptophan was used as a model *N*-Boc amine. l-Tryptophan (0.657 mmol) in HCl (3.94 mmol) in MeOH, was compared to an equal mmol of *N*-Boc l-tryptophan in oxalyl chloride (1.97 mmol) in MeOH system. After monitoring both reactions for 3 h, the *N*-Boc l-tryptophan had been completely converted to l-tryptophan in the oxalyl chloride–MeOH system, whereas the HCl–methanol system did not yield an observable deprotection of the *N*-Boc-l-tryptophan substrate. This suggests that the deprotection of *N*-Boc in oxalyl chloride–MeOH system involves a broader mechanism than simply *in situ* generation of HCl.

### Proposed mechanism

**Scheme 3:**
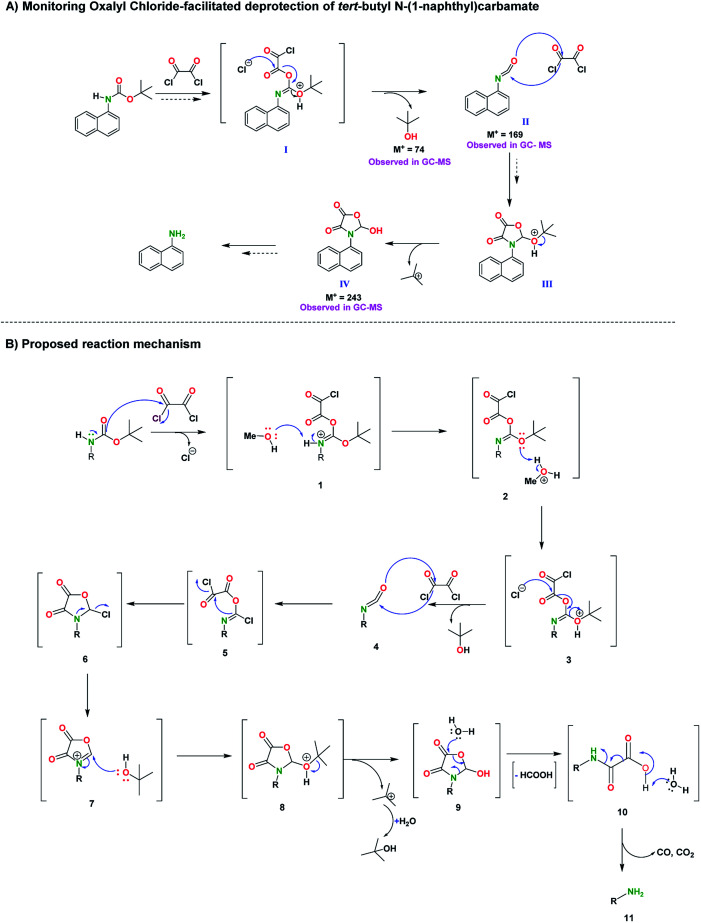
Proposed mechanism of oxalyl-chloride mediated deprotection of *N*-Boc group.

In the course of the reaction of the oxalyl chloride-mediated deprotection of model compound, *tert*-butyl *N*-(1-naphthyl)carbamate, the GC-products, isocyanate ester, II; the *tert*-butanol as well as the hydroxy-oxazolidinedione, IV (Scheme 3a) were observed. We therefore propose a possible mechanism for the oxalyl chloride mediated deprotection of the *N*-Boc group (Scheme 3b). The electrophilic character of oxalyl chloride present opportunities for unique reactivity. In this context, addition reactions of the carbonyl unit of the carbamate with oxalyl chloride is plausible. Therefore, it is possible that the intermediate, 1, can be formed from such addition reactions. It has been shown that the oxalyl chloride mediated reactions with carboxylic acid derivatives can yield isocyanate products.^37,38^ Therefore, it is not far-fetched that the removal of *tert* butanol, from intermediate, 3, yields the isocyanate ester intermediate, 4. The subsequent release of *tert* butyl ion from intermediate, 8 yields the oxazolidinedione-like intermediate, 9. It has been shown that the oxazolidinedione scaffold undergoes hydrolysis and ring opening to release carbon dioxide and related products.^39,40^ It is therefore plausible that after the workup, the oxazolidinedione-like intermediate, 9 will undergo a ring opening and subsequently yield the amine product, 11, by releasing carbon dioxide, carbon monoxide and possible formyl side products. It must be noted that the *tert* butyl cation side-product produced from the conversion of 8 to 9, will be transformed into isobutanol.

This *N*-Boc deprotection methodology may have limited applications in large scale organic processes due to the possible formation of the carbon monoxide side-product.

## Conclusion

In a nutshell, we have developed a simple method for the deprotection of *N*-Boc group under the mild conditions of oxalyl chloride and methanol. Our methodology should prove useful in total synthesis; for the deprotection of *N*-Boc of substrates in the presence of other functional groups. Also, we have the strongest conviction that this study will serve as a model for the subsequent method development for the oxalyl chloride-mediated direct transformation of *N*-Boc protected amines into amides.

## General information

Reagents for this study including oxalyl chloride, di-*tert*-butyl dicarbonate, triethylamine (TEA), and diethylisopropylamine (DIPEA) were purchased from VWR or Sigma Aldrich and used as is. All starting amines were purchased from VWR or Sigma Aldrich and used as is without further purification or drying. Solvents used for reactions were of ACS grade (Pharmco-Aaper) and used as is. Deuterated solvents were purchased from Cambridge Isotope Laboratories (Andover, MA).

### NMR spectra

All NMR spectra were recorded on a 400 MHz Bruker Avance NEO (equipped with a Smart Probe). Chemical shifts are reported as *δ*-values in ppm. The deuterated solvents, chloroform-d and acetone-d_6_ were used for all spectra with ppm values referenced to: CDCl_3_ (*δ*_H_: 7.26; *δ*_C_: 77.16); (CD_3_)_2_CO (*δ*_H_: 2.05 (p); *δ*_C_: 206.7 (m), 29.9 (sept)). Observed signal multiplicities are abbreviated as follows: s (singlet), d (doublet), t (triplet), q (quartet), quint (quintet), sext (sextet), sept (septet), m (multiplet), dd (doublet of doublet), ddd (doublet of doublet of doublets), dt (doublet of triplets). The coupling constants given are H–H coupling constants for proton signals.

Reaction monitoring *via* NMR was carried out using a 25 mg sample of EC1, dissolved in 0.75 mL of MeOD. A proton NMR spectrum was taken at 0, 2, 4, and 12 h (overnight). The sample was shaken on a mechanical shaking apparatus for the entire period of time between each spectra acquisition.

### Gas chromatographical analysis (GC)

GC-MS data were collected from an Agilent technologies 6890N GC with split injection and with 5973 MSD, both operated *via* a Chemstation software. Ultra-high purity helium was used as carrier gas at 0.5 mL min^−1^, after passing through an oxygen and moisture trap. Analytical samples for starting material analysis were prepared at a concentration of 1 g mL^−1^ in MeOH. To monitor representative reactions, samples were taken at 1 h intervals over a period of 5 h. Aliquots of reactions were sampled at respective time periods, dried over MgSO_4_, filtered, and then diluted to a concentration of 1 g mL^−1^ in MeOH. Following the automated injection of 0.1 mL, the oven was held at 70 °C for 3 minutes, then ramped at 10 degrees per minute to 280 degrees, where it was held for 22 minutes to complete the run.

### X-ray crystal structure determination

The X-ray crystal structure of EC1 was determined using a dual-microsource Bruker D8 Venture *κ*-axis diffractometer with CuKα (*λ* = 1.54178 Å) X-rays. Crystals were grown by slow vapor diffusion of hexane into a concentrated solution of compound EC1 dissolved in DCM. The crystal was placed directly into the cold gas stream of a liquid nitrogen cryostat.^1^ Raw data were integrated, scaled, merged, and corrected for Lorentz-polarization effects using the APEX3 package.^2^ Space group determination and structure solution and refinement were carried out with SHELXT^3^ and SHELXL^4^ respectively. All non-hydrogen atoms were refined with anisotropic displacement parameters. Hydrogen atoms were placed at calculated positions and refined using a riding model with their isotropic displacement parameters set to either 1.2 Uiso or 1.5 Uiso of the atom to which they were attached. Ellipsoid plots were drawn using SHELXTL-XP.^5^ The structures, deposited in the Cambridge Structural Database,^6^ were checked for missed symmetry, twinning, and overall quality with PLATON,^7^ an R-tensor,^8^ and finally validated using CheckCIF.

## Experimental procedures

### General procedure for *N*-Boc protection of amines

In a 100 mL round bottom flask equipped with a stirring bar, the starting amine (500 mg) and TEA (or DIPEA) (3 equiv.) was dissolved in 2 : 1 v/v mixture of H_2_O/THF (45 mL) and allowed to stir at room temperature for 5 min. Once all starting materials were completely dissolved, the reaction mixture was cooled to 0 °C before di-*tert*-butyl dicarbonate (1.5 equiv.) was added to the solution in one portion. The reaction mixture was stirred at 0 °C for at least 2 h and then allowed to warm to room temperature over 4 h. The reaction was monitored *via* TLC. Upon complete conversion to the *N*-Boc-protected amine, THF was removed *in vacuo* and the crude material was subsequently extracted with dichloromethane (20 mL), washed with deionized water twice (2 × 10 mL), and brine once (1 × 10 mL). The organic layer was dried over anhydrous MgSO_4_, filtered, and concentrated under low vacuum on a rotary evaporator. Most products did not require further purification to yield the pure protected amine.

### General procedure for the *N*-Boc deprotection

In a dry 25 mL round bottom flask equipped with a stirring bar, the starting material (50 mg) was dissolved in MeOH (3 mL) and allowed to stir at room temperature for 5 min. Oxalyl chloride (3 equiv.) was then added to the solution (*via* syringe or micropipette) directly into the reaction solvent mixture. Sputtering and an increase in temperature of the reaction mixture were observed immediately upon addition of the oxalyl chloride. The reaction mixture was allowed to stir for up to 4 h depending on the starting material. The reaction was monitored *via* TLC. Upon complete conversion of the *N*-Boc-protected amine, deionized water (5 mL) was added to the flask slowly. The crude material was subsequently extracted with dichloromethane (5 mL) and washed with deionized water twice (2 × 5 mL). The organic layer was dried over anhydrous MgSO_4_, filtered, and concentrated under low vacuum on a rotary evaporator. Some products required further purification *via* flash column chromatography to yield the pure deprotected amine.

#### *tert*-Butyl *N*-(2-isopropylphenyl)carbamate (entry 1a)

Prepared according to general *N*-Boc procedure. Product: red liquid (870 mg, 3.69 mmol, 100%).

^1^H NMR [CDCl_3_, 400 MHz]: *δ* 7.62 (d, 1H, CH, *J* = 7.6 Hz), *δ* 7.17 (dd, 1H, CH, *J* = 8, 1.6 Hz), *δ* 7.11 (td, 1H, CH, *J* = 7.6, 1.2 Hz) *δ* 7.03 (td, 1H, CH, *J* = 7.6, 1.2 Hz), *δ* 6.22 (s, 1H, NH), *δ* 2.91 (sep, 1H, CH, *J* = 6.8 Hz), *δ* 1.44 (s, 9H, (CH_3_)_3_), *δ* 1.17 (d, 6H, (CH_3_)_2_, *J* = 6.8 Hz).

^13^C NMR [CDCl_3_, 101 MHz]: *δ* 153.6, 139.1, 134.7, 126.3, 125.4, 124.7, 123.1, 80.3, 28.4, 27.7, 23.0.

The spectral data of the compound 1a was consistent with the values reported in the literature.^41^

#### 2-Isopropylaniline (entry 1b)

Prepared according to general deprotection procedure. Product: pale-yellow liquid (40 mg, 0.29 mmol, 70%).

^1^H NMR [CDCl_3_, 400 MHz]: *δ* 7.14 (dd, 1H, CH, *J* = 7.6, 1.6 Hz), *δ* 7.02 (td, 1H, CH, *J* = 7.6, 1.2 Hz), *δ* 6.80 (td, 1H, CH, *J* = 7.6, 1.2 Hz), *δ* 6.71 (dd, 1H, CH, *J* = 7.6, 1.6 Hz), *δ* 3.96 (s, 2H, NH_2_), *δ* 2.91 (sep, 1H, CH, *J* = 6.8 Hz), *δ* 1.25 (d, 6H, (CH_3_)_2_, *J* = 6.8 Hz).

^13^C NMR [CDCl_3_, 101 MHz]: *δ* 142.6, 133.2, 126.5, 125.5, 119.5, 116.3, 27.7, 22.3.

The spectral data of the compound 1b was consistent with the values reported in the literature.^42^

#### *tert*-Butyl *N*-(1-naphthyl)carbamate (entry 2a)

Prepared according to general *N*-Boc procedure. Product: off-white solid (849 mg, 3.49 mmol, 100%).

^1^H NMR [CDCl_3_, 400 MHz]: *δ* 7.89 (m, 3H, CH), *δ* 7.66 (d, 1H, CH, *J* = 8 Hz), *δ* 7.52 (m, 3H, CH), *δ* 6.93 (s, 1H, NH), *δ* 1.61 (s, 9H, (CH_3_)_3_).

^13^C NMR [CDCl_3_, 101 MHz]: *δ* 153.6, 134.1, 133.0, 128.7 (2C), 126.0 (2C), 125.9, 125.8, 124.5, 120.5, 118.8 (2C), 80.7, 28.4.

Melting point: 95–97 °C.

The spectral data of the compound 2a was consistent with the values reported in the literature.^43^

#### Naphthylamine (entry 2b)

Prepared according to general deprotection procedure. Product: brown solid (51 mg, 0.35 mmol, 87%).

^1^H NMR [CDCl_3_, 400 MHz]: *δ* 7.92 (m, 1H, CH), *δ* 7.69 (m, 1H, CH), *δ* 7.36 (m, 2H, CH), *δ* 7.19 (d, 2H, CH, *J* = 4.4 Hz), *δ* 6.77 (pen, 1H, CH, *J* = 4.4 Hz), *δ* 4.81 (s, 2H, NH_2_).

^13^C NMR [CD_3_OD, 101 MHz]: *δ* 145.4, 137.0, 130.3 (2C), 128.4, 127.7, 123.6, 120.4, 111.8 (2C).

Melting point: 49–52 °C.

The spectral data of the compound 2b was consistent with the values reported in the literature.^44^

#### *tert*-Butyl *N*-(3-chlorophenyl)carbamate (entry 3a)

Prepared according to general *N*-Boc procedure. Product: white solid (892 mg, 3.9 mmol, 100%).

^1^H NMR [CDCl_3_, 400 MHz]: *δ* 7.44 (singlet, 1H, CH), *δ* 7.09 (m, 2H, CH), *δ* 6.91 (dt, 1H, CH, *J* = 7.2, 1.6 Hz), *δ* 6.48 (s, 1H, NH), *δ* 1.44 (s, 9H, (CH_3_)_3_).

^13^C NMR [CDCl_3_, 101 MHz]: *δ* 152.5, 139.6, 134.7, 129.9, 123.0, 118.6, 116.5, 28.3.

Melting point: 69–70 °C.

The spectral data of the compound 3a was consistent with the values reported in the literature.^45^

#### 3-Chloroaniline (entry 3b)

Prepared according to general deprotection procedure. Product: yellow liquid (46 mg, 0.36 mmol, 83%).

^1^H NMR [CDCl_3_, 400 MHz]: *δ* 7.03 (t, 1H, CH, *J* = 8 Hz), *δ* 7.70 (ddd, 1H, CH, *J* = 8, 2, 0.8 Hz), *δ* 6.91 (t, 1H, CH, *J* = 2 Hz), *δ* 6.50 (ddd, 1H, CH, *J* = 8, 2, 0.8 Hz), *δ* 3.648 (s, 2H, NH_2_).

^13^C NMR [CDCl_3_, 101 MHz]: *δ* 147.7, 134.9, 130.3, 118.5, 115.0, 113.2.

The spectral data of the compound 3b was consistent with the values reported in the literature.^46^

#### *tert*-Butyl *N*-(3-chloro-4-fluorophenyl)carbamate (entry 4a)

Prepared according to general *N*-Boc procedure. Product: solid (843 mg, 3.4 mmol, 100%).

^1^H NMR [CDCl_3_, 400 MHz]: *δ* 7.575 (dd, 1H, CH, *J* = 6.2, 2 Hz), *δ* 7.138 (ddd, 1H, CH, *J* = 8.8, 4, 2.8 Hz), *δ* 7.047 (t, 1H, CH, *J* = 8.8 Hz), *δ* 6.554 (s, 1H, NH), *δ* 1.53 (s, 9H, (CH_3_)_3_).

^13^C NMR [CDCl_3_, 101 MHz]: *δ* 153.9, 136.4, 135.6, 131.5, 128.8, 79.7, 28.3, 20.9, 18.2.

Melting point: 130–132 °C.

The spectral data of the compound 4a was consistent with the values reported in the literature.^47^

#### 3-Chloro-4-fluoroaniline (entry 4b)

Prepared according to general deprotection procedure. Product: solid (47 mg, 0.32 mmol, 80%).

^1^H NMR [CDCl_3_, 400 MHz]: *δ* 6.91 (t, 1H, CH, *J* = 8.8 Hz), *δ* 6.68 (dd, 1H, CH, *J* = 6.4, 2.8 Hz) *δ* 6.50 (ddd, 1H, CH, *J* = 8.8, 4, 2.8 Hz), *δ* 3.48 (s, 2H, NH_2_).

^13^C NMR [CDCl_3_, 101 MHz]: *δ* 152.8, 150.5, 143.2, 143.2, 121.1, 120.9, 116.9, 116.7, 116.4, 114.3, 114.2.

Melting point: 46–48 °C.

The spectral data of the compound 4b was consistent with the values reported in the literature.^48^

#### *tert*-Butyl *N*-(2,4,6-trimethylphenyl)carbamate (entry 5a)

Prepared according to general *N*-Boc procedure. Product: off-white solid (870 mg, 3.69 mmol, 100%).

^1^H NMR [CDCl_3_, 400 MHz]: *δ* 6.91 (t, 1H, CH, *J* = 8.8 Hz), *δ* 6.68 (dd, 1H, CH, *J* = 6.4, 2.8 Hz) *δ* 6.50 (ddd, 1H, CH, *J* = 8.8, 4, 2.8 Hz), *δ* 3.48 (s, 2H, NH_2_).

^13^C NMR [CDCl_3_, 101 MHz]: *δ* 153.9, 136.4, 135.6 (2C), 128.8, 79.7, 28.3, 20.9, 18.2.

The spectral data of the compound 5a was consistent with the values reported in the literature.^49^

#### 2,4,6-Trimethylaniline (entry 5b)

Prepared according to general deprotection procedure. Product: pale yellow liquid (47 mg, 0.35 mmol, 83%).

^1^H NMR [CDCl_3_, 400 MHz]: *δ* 6.63 (s, 2H, CH), *δ* 2.10 (s, 3H, CH_3_) *δ* 2.07 (s, 6H, CH_3_).

^13^C NMR [CD_3_OD, 101 MHz]: *δ* 142.3, 130.8, 129.5, 124.7, 21.7, 18.9.

The spectral data of the compound 5b was consistent with the values reported in the literature.^50^

#### *tert*-Butyl *N*-(2,6-diisopropylphenyl)carbamate (entry 6a)

Prepared according to general *N*-Boc procedure. Product: pale red solid (782 mg, 2.82 mmol, 100%).

^1^H NMR [CDCl_3_, 400 MHz]: *δ* 7.24 (t, 1H, CH, *J* = 8 Hz), *δ* 7.107 (d, 1H, CH, *J* = 8 Hz), *δ* 5.82 (s, 1H, NH), *δ* 3.16 (s, 2H, 2(CH)), *δ* 1.505 (s, 9H, (CH_3_)_3_), *δ* 1.17 (d, 12H, 2(CH_3_)_2_, *J* = 6.8 Hz).

^13^C NMR [CDCl_3_, 101 MHz]: *δ* 153.9, 136.4, 135.6, 131.5, 128.8, 79.7, 28.3, 20.9, 18.2.

Melting point: 145–148 °C.

The spectral data of the compound 6a was consistent with the values reported in the literature.^51^

#### 2,6-Diisopropylaniline (entry 6b)

Prepared according to general deprotection procedure. Product: red liquid (49 mg, 0.28 mmol, 78%).

^1^H NMR [CDCl_3_, 400 MHz]: *δ* 7.06 (d, 2H, CH, *J* = 8 Hz), *δ* 6.83 (t, 1H, CH, *J* = 7.6 Hz), *δ* 3.78 (s, 2H, NH_2_), *δ* 2.97 (sep, 2H, 2(CH), *J* = 6.8 Hz), *δ* 1.30 (d, 12H, 2(CH_3_)_2_, *J* = 6.8 Hz).

^13^C NMR [CDCl_3_, 101 MHz]: *δ* 140.2, 132.5, 122.8, 118.6, 28.0, 22.5, 21.9.

The spectral data of the compound 6b was consistent with the values reported in the literature.^52^

#### *tert*-Butyl *N*-(4-nitrophenyl)carbamate (entry 7a)

Prepared according to general *N*-Boc procedure. Product: pale yellow solid (743.4 mg, 3.12 mmol, 86.2%).

^1^H NMR [CDCl_3_, 400 MHz]: *δ* 7.15 (d, 2H, CH, *J* = 8.6 Hz), 6.71 (d, 2H, CH, *J* = 8.9 Hz), 1.48 (s, 9H, (CH_3_)_3_).

^13^C NMR [CDCl_3_, 101 MHz]: *δ* 153.4, 151.9, 131.2, 121.7, 115.7, 80.4, 28.3.

Melting point: 112–113 °C.

The spectral data of the compound 7a was consistent with the values reported in the literature.^53^

#### 3-Nitroaniline (entry 7b)

Prepared according to general deprotection procedure. Product: yellow solid (22 mg, 0.16 mmol, 76%).

^1^H NMR [CD_3_OD, 400 MHz]: *δ* 7.92 (d, 2H, CH, *J* = 9.1 Hz), 6.71 (d, 2H, CH, *J* = 9.1 Hz).

Melting point: 146–147 °C.

The spectral data of the compound 7b was consistent with the values reported in the literature.^54^

#### *tert*-Butyl *N*-(3-bromo-4-fluorophenyl)carbamate (entry 8a)

Prepared according to general *N*-Boc procedure. Product: pale yellow solid (763 mg, 2.63 mmol, 100%).

^1^H NMR [CDCl_3_, 400 MHz]: *δ* 7.72 (dd, 1H, CH, *J* = 5.8, 2.8 Hz), *δ* 7.202 (ddd, 1H, CH, *J* = 8.8, 4, 2.8 Hz), *δ* 7.045 (t, 1H, CH, *J* = 8.8 Hz), *δ* 6.456 (s, 1H, CH), *δ* 1.534 (s, 9H, (CH_3_)_3_).

^13^C NMR [CDCl_3_, 101 MHz]: *δ* 156.3, 152.5, 135.3, 123.4, 118.9, 116.4, 109.1, 81.1, 28.3.

#### 3-Bromo-4-fluoroaniline (entry 8b)

Prepared according to general deprotection procedure. Product: brown liquid (57 mg, 0.30 mmol, 88%).

^1^H NMR [CDCl_3_, 400 MHz]: *δ* 6.89 (t, 1H, CH, *J* = 8.8 Hz), *δ* 6.84 (dd, 1H, CH, *J* = 5.6, 2.8 Hz) *δ* 6.54 (ddd, 1H, CH, *J* = 8.8, 4, 2.8 Hz), *δ* 3.57 (s, 2H, NH_2_).

^13^C NMR [CDCl_3_, 101 MHz]: *δ*^13^C NMR (101 MHz, CDCl_3_) *δ* 154.2, 151.8, 143.8, 119.5, 117.1, 115.4, 109.5.

The spectral data of the compound 8b was consistent with the values reported in the literature.^55^

#### *tert*-Butyl (4-iodophenyl)carbamate (entry 9a)

Prepared according to general *N*-Boc procedure. Product: off-white solid (710.2 mg, 2.22 mmol, 97.5%).

^1^H NMR [CDCl_3_, 400 MHz]: *δ* 7.56 (s, 1H), 7.54 (s, 1H), 7.12 (d, *J* = 8.7 Hz, 2H), 6.44 (s, 1H), 1.49 (d, *J* = 0.6 Hz, 9H).

^13^C NMR [CDCl_3_, 101 MHz]: *δ* 152.5, 138.2, 120.4, 85.7, 80.9, 30.9, 28.3.

The spectral data of the compound 9a was consistent with the values reported in the literature.^56^

Melting point: 143.7–147 °C.

#### 4-Iodoaniline (entry 9b)

Prepared according to general deprotection procedure. Product brown solid: (29.5 mg, 0.13 mmol, 86%).

^1^H NMR [CDCl_3_, 400 MHz]: *δ* 7.63 (d, *J* = 7.3 Hz, 2H), 6.71 (d, *J* = 7.4 Hz, 2H), 4.21 (s, 2H).

Melting point: 67–68 °C.

The spectral data of the compound 9b was consistent with the values reported in the literature.^57^

#### *tert*-Butyl *N*-(*N*-(2-methoxyphenyl)piperazine)carbamate (entry 10a)

Prepared according to general *N*-Boc protection procedure. Product: white solid (760 mg, 2.6 mmol, 100%).

^1^H NMR [CDCl_3_, 400 MHz]: *δ* 7.11 (t, 1H, CH, *J* = 7.4 Hz), *δ* 6.93 (m, 2H, CH), *δ* 3.90 (s, 3H, CH_3_), *δ* 3.72 (s, 4H, (CH_2_)_2_), *δ* 3.15 (s, 4H, (CH_2_)_2_), *δ* 1.50 (s, 9H, (CH_3_)_3_).

^13^C NMR [CDCl_3_, 400 MHz]: *δ* 154.7 (2C), 152.3, 121.3 (2C), 111.8 (2C), 79.9, 55.5, 50.9, 28.4.

Melting point: 68–69 °C.

The spectral data of the compound 10b was consistent with the values reported in the literature.^58^

#### *N*-(2-Methoxyphenyl)piperazine (entry 10b)

Prepared according to general deprotection procedure. Product: (42 mg, 0.21 mmol, 64%).

^1^H NMR [CDCl_3_, 400 MHz]: *δ* 6.89 (m, 1H, CH), *δ* 6.83 (m, 2H, CH) *δ* 6.77 (m, 1H, CH), *δ* 3.57 (s, 3H, CH_3_), *δ* 2.94 (m, 8H, 4(CH_2_)).

^13^C NMR [CDCl_3_, 101 MHz]: *δ* 152.3, 141.8, 122.9, 121.0, 118.2, 111.4, 55.3, 52.0, 46.3.

Melting point: 37–40 °C.

The spectral data of the compound 10b was consistent with the values reported in the literature.^59^

#### *tert*-Butyl *N*-(*N*-(4-thiophene-phenyl)piperazine)carbamate (entry 11a)

Prepared according to general *N*-Boc procedure. Yield: (704 mg, 2.04 mmol, 100%).

^1^H NMR [CDCl_3_, 400 MHz]: *δ* 7.52 (d, 2H, CH, *J* = 8.8 Hz), *δ* 7.18 (d, 2H, CH, *J* = 4.4 Hz), *δ* 7.04 (m, 1H, CH), *δ* 6.92 (d, 2H, CH, *J* = 9.2 Hz), *δ* 3.58 (t, 4H, CH_2_, *J* = 5.2 Hz), *δ* 3.16 (t, 4H, CH_2_, *J* = 5.2 Hz), *δ* 1.49 (s, 9H, (CH_3_)_3_).

^13^C NMR [CD_3_OD, 101 MHz]: 154.7, 150.5, 144.5, 127.9, 126.9, 126.4, 123.6, 121.8, 116.5, 79.9, 49.1, 28.4.

#### *N*-(4-thiophene-phenyl)piperazine (entry 11b)

Prepared according to general deprotection procedure. Product: off-white solid (61 mg, 0.25 mmol, 87%).

^1^H NMR [CDCl_3_, 400 MHz]: *δ* 7.54 (d, 2H, CH, *J* = 9.2 Hz), *δ* 7.27 (m, 2H, CH) *δ* 7.02 (m, 3H, CH), *δ* 3.44 (m, 4H, CH_2_), *δ* 3.38 (m, 4H, CH_2_).

^13^C NMR [CD_3_OD, 101 MHz]: *δ* 149.7, 143.9, 128.8, 126.7, 124.6, 122.6, 116.6, 45.7, 43.0.

#### *tert*-Butyl *N*-(cyclohexyl)carbamate (entry 12a)

Prepared according to general procedure. Product: colourless solid (1.0 g, 5.04 mmol, 100%).

^1^H NMR [CDCl_3_, 400 MHz]: *δ* 4.41 (s, 1H, CH), *δ* 3.43 (s, 1H, NH), *δ* 1.92 (m, 2H, CH), *δ* 1.69 (m, 3H, CH), *δ* 1.46 (s, 9H, (CH_3_)_3_)*δ* 1.36 (m, 2H, CH_2_), *δ* 1.12 (m, 3H, CH).

Melting point: 79–80 °C.

The spectral data of the compound 12a was consistent with the values reported in the literature.^60^

#### Cyclohexylamine (entry 12b)

Prepared according to general deprotection procedure. Product: colourless liquid (27 mg, 0.28 mmol, 55%).

^1^H NMR [CDCl_3_, 400 MHz]: *δ* 2.63 (s, 1H, CH), *δ* 1.93 (m, 2H, CH_2_), *δ* 1.70 (m, 3H, CH), *δ* 1.58 (d, 1H, CH, *J* = 12.4 Hz), *δ* 1.26 (q, 2H, CH_2_, *J* = 12.4 Hz), *δ* 1.11 (p, 3H, CH, *J* = 12.4 Hz).

The spectral data of the compound 12b was consistent with the values reported in the literature.^61^

#### *tert*-Butyl *N*-(2-[2-(2-aminoethoxy)ethoxy]ethanamine)carbamate (entry 13a)

Prepared according to general procedure. Product: pale yellow liquid (318 mg, 1.2 mmol, 38%).

^1^H NMR [CDCl_3_, 400 MHz]: *δ* 4.13 (s, 1H, CH), *δ* 3.48 (s, 3H, CH), *δ* 3.41 (s, 3H, CH), *δ* 3.18 (s, 2H, CH_2_), *δ* 2.74 (s, 1H, CH), *δ* 1.78 (s, 1H, CH_2_), *δ* 1.31 (s, 9H, (CH_3_)_3_).

The spectral data of the compound 13a was consistent with the values reported in the literature.^62^

#### 2-[2-(2-Aminoethoxy)ethoxy]ethanamine (entry 13b)

Prepared according to general procedure. Product: colourless liquid (49 mg, 0.4 mmol, 90%).

^1^H NMR [CDCl_3_, 400 MHz]: *δ* 3.84 (m, 4H, CH_2_), *δ* 3.73 (t, 2H, CH_2_, *J* = 5.0 Hz), *δ* 3.70 (d, 2H, CH_2_, *J* = 1.2 Hz), *δ* 3.64 (s, 1H, CH), *δ* 3.32 (d, 1H, CH, *J* = 1.6 Hz), *δ* 3.14 (t, 2H, CH_2_, *J* = 4.6 Hz).

The spectral data of the compound 13b was consistent with the values reported in the literature.^63^

#### EC1

1-Methyl tryptophan was protected prior to synthesis of the conjugate molecule. Prepared according to general procedure.

^1^H NMR [(CD_3_)_2_CO, 400 MHz]: *δ* 8.06 (d, 1H, CH, *J* = 8.4 Hz), *δ* 7.65 (d, 1H, CH, *J* = 8 Hz), *δ* 7.55 (d, 1H, CH_2_, *J* = 2.4 Hz), *δ* 7.38 (m, 2H, CH), *δ* 7.20 (m, 2H, CH), *δ* 7.07 (m, 1H, CH), *δ* 6.91 (s, 1H, CH), *δ* 4.73 (m, 1H, CH), *δ* 3.83 (s, 3H, CH_3_), *δ* 3.77 (s, 1H, NH), *δ* 3.42 (m, 2H, CH_2_), *δ* 2.16 (s, 3H, CH_3_), *δ* 1.43 (s, 9H, (CH_3_)_3_).

^13^C NMR [(CD_3_)_2_CO, 101 MHz]: *δ* 184.1, 183.6, 170.7, 155.6, 155.0, 148.4, 137.3, 135.3, 133.9, 129.9, 128.1, 126.8, 121.5, 118.9, 118.6, 118.6, 109.5, 108.9, 78.9, 55.3, 31.9, 27.7, 27.1, 15.4.

### Determination of HCl effectiveness in deprotection

The study by Nudelman *et al.*^64^ supported the idea that equal-molar ratios of HCl in solution could deprotect Boc-protected carbamate in the presence of methanol or other alcohols. Excess HCl in solution has already been shown to deprotect *N*-Boc groups by forming the amino-chloride salt. In order to determine if the deprotection of *N*-Boc groups was carried out by *in situ* production of HCl, or by a more complex mechanism directly utilizing the chlorine source molecule, concurrent reactions were set in which one reaction utilized the proposed oxalyl chloride deprotection set up, and the other was run with solely HCl in MeOH of the Boc-protected amine. The HCl reaction was carefully set up to only contain the exact molar amount of HCl that would be produced if all chlorine atoms dissociated from oxalyl chloride in the oxalyl chloride reaction. Analysis of each reaction after 6 h showed no removal of the *N*-BOC group when only HCl was used as the deprotecting agent, but complete removal of the *N*-BOC group when oxalyl chloride was used as the deprotecting agent.

## Conflicts of interest

There are no conflicts to declare.

## Supplementary Material

RA-010-D0RA04110F-s001

RA-010-D0RA04110F-s002
