# Correction: Mild deprotection of the *N-tert*-butyloxycarbonyl (*N*-Boc) group using oxalyl chloride

**DOI:** 10.1039/d0ra90135k

**Published:** 2021-01-04

**Authors:** Nathaniel George, Samuel Ofori, Sean Parkin, Samuel G. Awuah

**Affiliations:** Department of Chemistry, University of Kentucky Lexington Kentucky 40506 USA awuah@uky.edu

## Abstract

Correction for ‘Mild deprotection of the *N-tert*-butyloxycarbonyl (*N*-Boc) group using oxalyl chloride’ by Nathaniel George *et al.*, *RSC Adv.*, 2020, **10**, 24017–24026, DOI: 10.1039/D0RA04110F.

The authors regret the omission of a funding acknowledgement in the original article. This acknowledgement is given below.

The authors acknowledge support of the Center for Pharmaceutical Research and Innovation (NIH P20 GM130456).

The Royal Society of Chemistry apologises for these errors and any consequent inconvenience to authors and readers.

## Supplementary Material

