# Supplementary material for: Mild deprotection of the N-tert-butyloxycarbonyl (N-Boc) group using oxalyl chloride
Source: RSC Adv. 2020 Jun 23;10(40):24017–26. doi: 10.1039/d0ra04110f (PMC7810210; doi:10.1039/d0ra04110f)
Supplement: RA-010-D0RA04110F-s001 [file RA-010-D0RA04110F-s001.pdf]

Electronic Supplementary Material (ESI) for RSC Advances.
This journal is © The Royal Society of Chemistry 2020

Mild Deprotection of *N-tert*-butyloxycarbonyl (*N*-Boc) Group Using Oxalyl Chloride

Nathaniel George‡, Samuel Ofori‡ and Samuel G. Awuah*
Department of Chemistry, University of Kentucky, Lexington Kentucky 40506, United States
Correspondence to: awuah@uky.edu

Table of Contents

^{1}H NMR spectrum of *tert*-butyl *N*-(2-isopropylphenyl)carbamate in $CDCl_3$ S3

^{13}C NMR spectrum of *tert*-butyl *N*-(2-isopropylphenyl)carbamate in $CDCl_3$ S3

^{1}H NMR spectrum of *tert*-butyl *N*-(1-naphthyl)carbamate in $CDCl_3$ S4

^{13}C NMR spectrum of *tert*-butyl *N*-(1-naphthyl)carbamate in $CDCl_3$ S4

^{1}H NMR spectrum of *tert*-butyl *N*-(3-chlorophenyl)carbamate in $CDCl_3$ S5

^{13}C NMR spectrum of *tert*-butyl *N*-(3-chlorophenyl)carbamate in $CDCl_3$ S5

^{1}H NMR spectrum of *tert*-butyl *N*-(3-chloro-4-fluorophenyl)carbamate in $CDCl_3$ S6

^{13}C NMR spectrum of *tert*-butyl *N*-(3-chloro-4-fluorophenyl)carbamate in $CDCl_3$ S6

^{1}H NMR spectrum of *tert*-butyl *N*-(2,4,6-trimethylphenyl)carbamate in $CDCl_3$ S7

^{13}C NMR spectrum of *tert*-butyl *N*-(2,4,6-trimethylphenyl)carbamate in $CDCl_3$ S7

^{1}H NMR spectrum of *tert*-butyl *N*-(2,6-diisopropylphenyl)carbamate in $CDCl_3$ S8

^{13}C NMR spectrum of *tert*-butyl *N*-(2,6-diisopropylphenyl)carbamate in $CDCl_3$ S8

^{1}H NMR spectrum of *tert*-butyl *N*-(4-nitrophenyl)carbamate in $CDCl_3$ S9

^{13}C NMR spectrum of *tert*-butyl *N*-(4-nitrophenyl)carbamate in $CDCl_3$ S9

^{1}H NMR spectrum of *tert*-butyl *N*-(3-bromo-4-fluorophenyl)carbamate in $CDCl_3$ S10

^{13}C NMR spectrum of *tert*-butyl *N*-(3-bromo-4-fluorophenyl)carbamate in $CDCl_3$ S10

^{1}H NMR spectrum of *tert*-Butyl (4-iodophenyl)carbamate in $CDCl_3$ S11

^{13}C NMR spectrum of *tert*-Butyl (4-iodophenyl)carbamate in $CDCl_3$ S11

^{1}H NMR spectrum of *tert*-butyl *N*-(N-(2-Methoxyphenyl)piperazine)carbamate in $CDCl_3$ S12

^{13}C NMR spectrum of *tert*-butyl *N*-(N-(2-Methoxyphenyl)piperazine)carbamate in $CDCl_3$........................S12

^{1}H NMR spectrum of *tert*-butyl *N*-(N-(4-thiophene-phenyl)piperazine)carbamate in $CDCl_3$. S13

^{13}C NMR spectrum of *tert*-butyl *N*-(N-(4-thiophene-phenyl)piperazine)carbamate in $CDCl_3$..................S13

^{1}H NMR spectrum of *tert*-butyl *N*-(cyclohexyl)carbamate in $CDCl_3$ S14

^{1}H NMR spectrum of *tert*-butyl *N*-(2-[2-(2-aminoethoxy)ethoxy]ethanamine)carbamate S14

^{13}C NMR spectrum of *tert*-butyl *N*-(2-[2-(2-aminoethoxy)ethoxy]ethanamine)carbamate S15

^{1}H NMR spectrum of 2-isopropylaniline in $CDCl_3$ S16

^{13}C NMR spectrum of 2-isopropylaniline in $CDCl_3$ S16

^{1}H NMR spectrum of Naphthylamine in $CDCl_3$ S17

^{13}C NMR spectrum of Naphthylamine in MeOD S17

^{1}H NMR spectrum of 3-chloroaniline in $CDCl_3$ S18

^{13}C NMR spectrum of 3-chloroaniline in $CDCl_3$ S18

^{1}H NMR spectrum of 3-chloro-4-flouroaniline in $CDCl_3$ S19

^{13}C NMR spectrum of 3-chloro-4-flouroaniline in $CDCl_3$ S19

^{1}H NMR spectrum of 2,4,6-trimethylaniline in $CDCl_3$ S20

^{13}C NMR spectrum of 2,4,6-trimethylaniline in MeOD S20

^{1}H NMR spectrum of 2,6-diisopropylaniline in $CDCl_3$ S21

^{13}C NMR spectrum of 2,6-diisopropylaniline in $CDCl_3$ S21

^{1}H NMR spectrum of 4-Nitroaniline in $CDCl_3$ S22

^{1}H NMR spectrum of 3-bromo-4-fluoroaniline in $CDCl_3$ S22

^{13}C NMR spectrum of 3-bromo-4-fluoroaniline in $CDCl_3$ S23

^{1}H NMR spectrum of 4-iodoaniline in $CDCl_3$ S23

^{1}H NMR spectrum of N-(2-Methoxyphenyl)piperazine in $CDCl_3$ S24

^{13}C NMR spectrum of N-(2-Methoxyphenyl)piperazine in $CDCl_3$ S24

^{1}H NMR spectrum of N-(4-thiophene-phenyl)piperazine in $CDCl_3$ S25

^{13}C NMR spectrum of N-(4-thiophene-phenyl)piperazine in $CDCl_3$ S25

^{1}H NMR spectrum of cyclohexylamine in $CDCl_3$ S26

^{1}H NMR spectrum of 2-[2-(2-aminoethoxy)ethoxy]ethanamine in $CDCl_3$ S26

EC1 Deprotection Utilizing Oxalyl Chloride... S24

Determination of HCl effectiveness in Deprotection.. S31

X-Ray Crystal Compound 4 (EC1) S33

Real time GC-MS spectra of oxalyl chloride deprotection S34

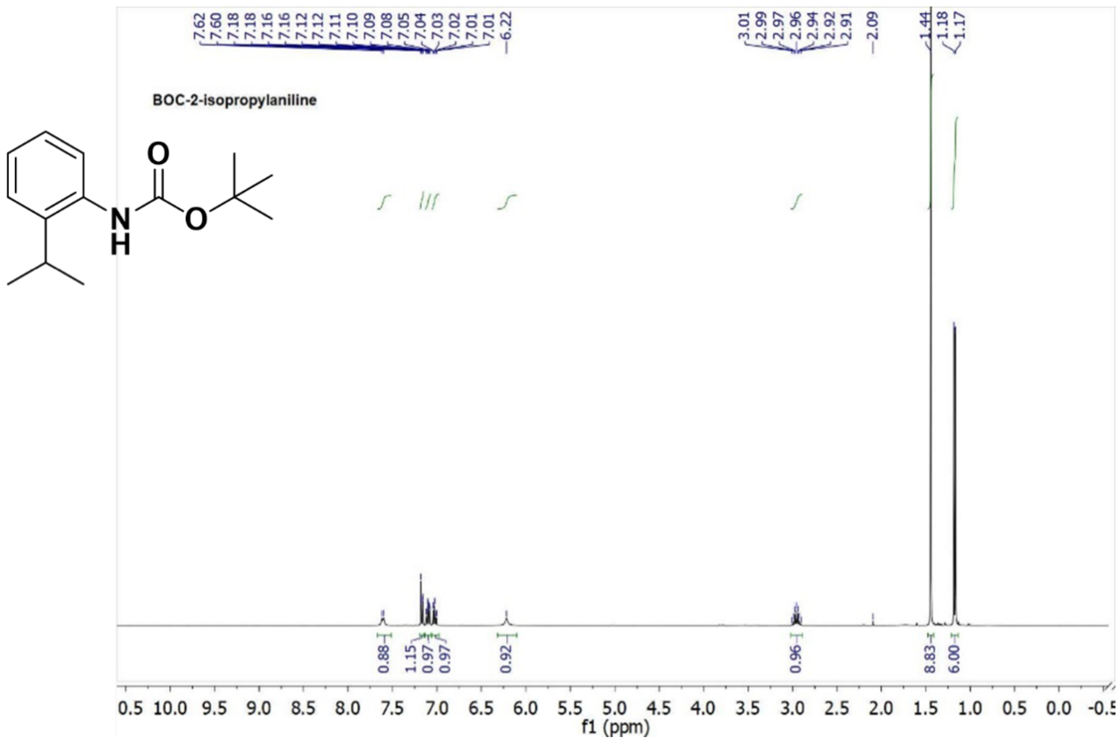

Figure S1: ^{1}H NMR spectrum of *tert*-butyl N-(2-isopropylphenyl)carbamate (Entry 1a) in $CDCl_3$

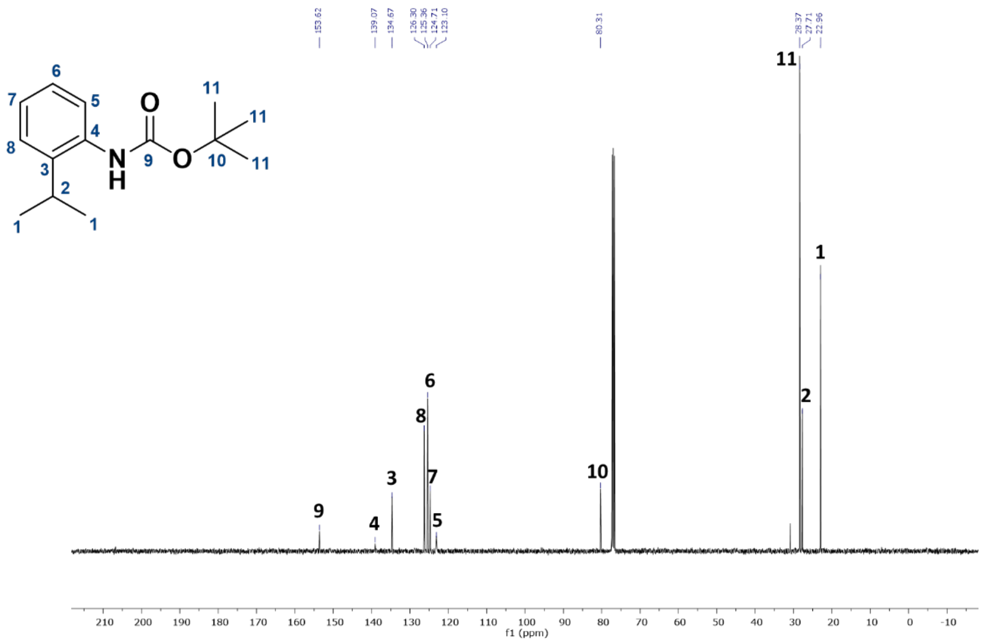

Figure S2: ^{13}C NMR spectrum of *tert*-butyl *N*-(2-isopropylphenyl)carbamate (Entry 1a) in $CDCl_3$

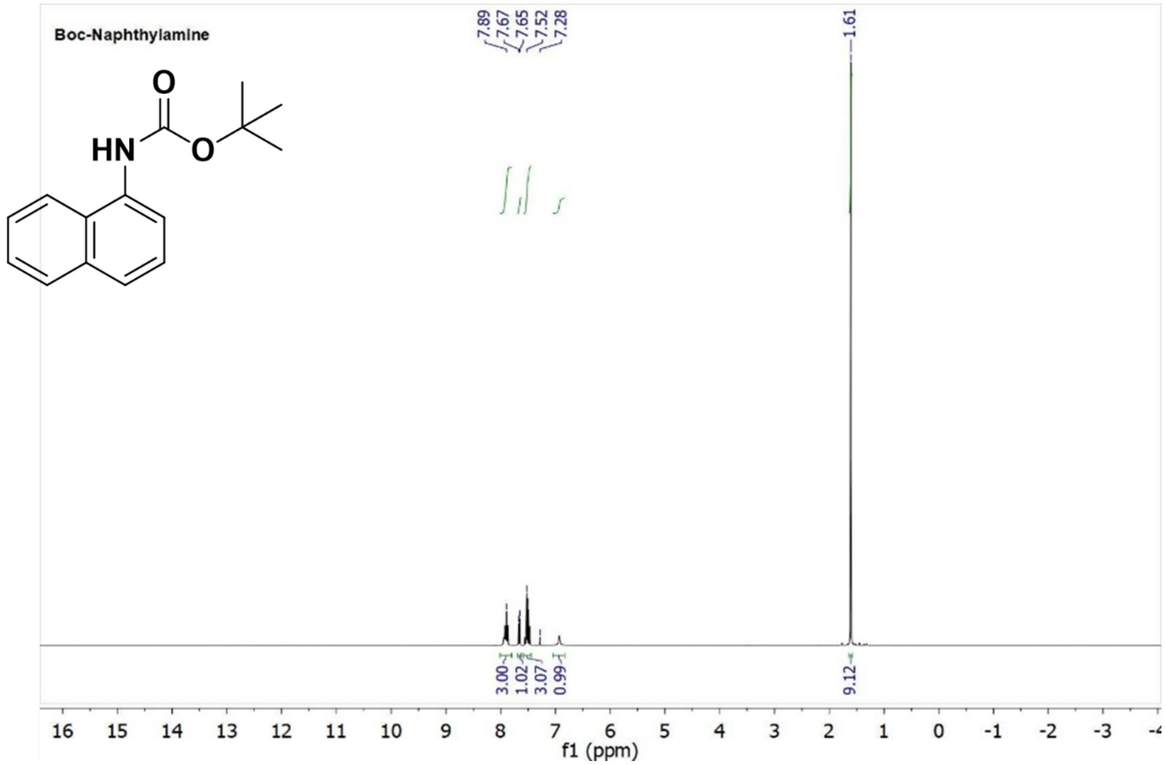

Figure S3: ^{1}H NMR spectrum of *tert*-butyl *N*-(1-naphthyl)carbamate (Entry 2a) in $CDCl_3$

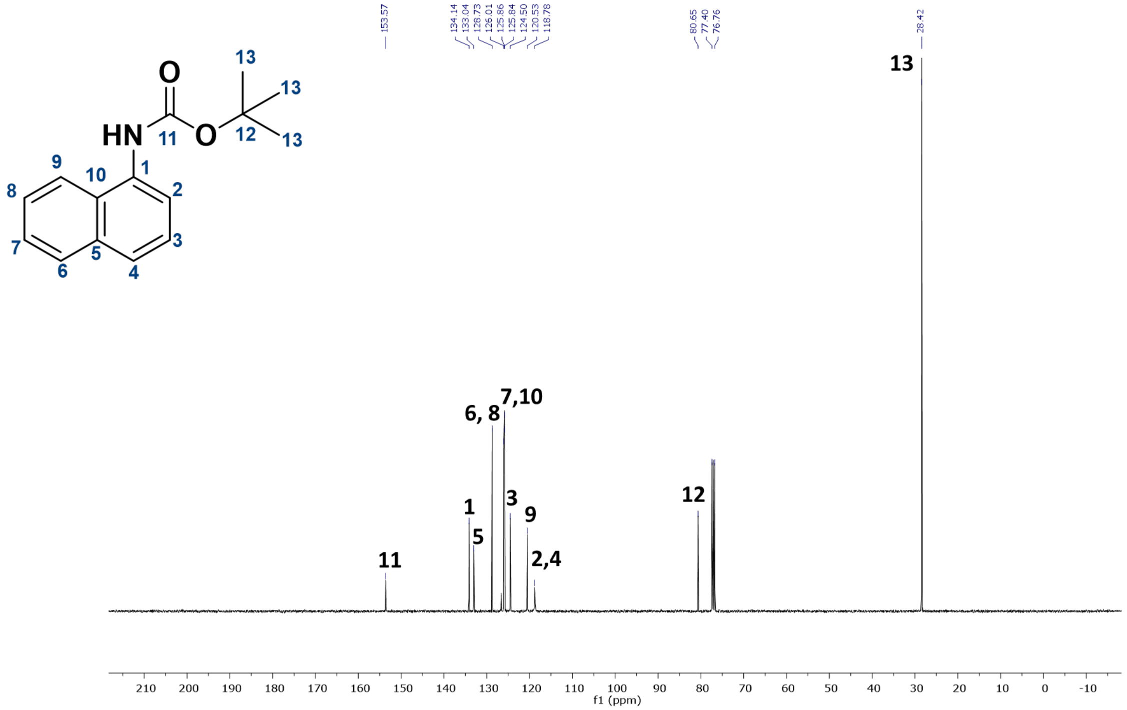

Figure S4: ^{13}C NMR spectrum of *tert*-butyl *N*-(1-naphthyl)carbamate (Entry 2a) in $CDCl_3$

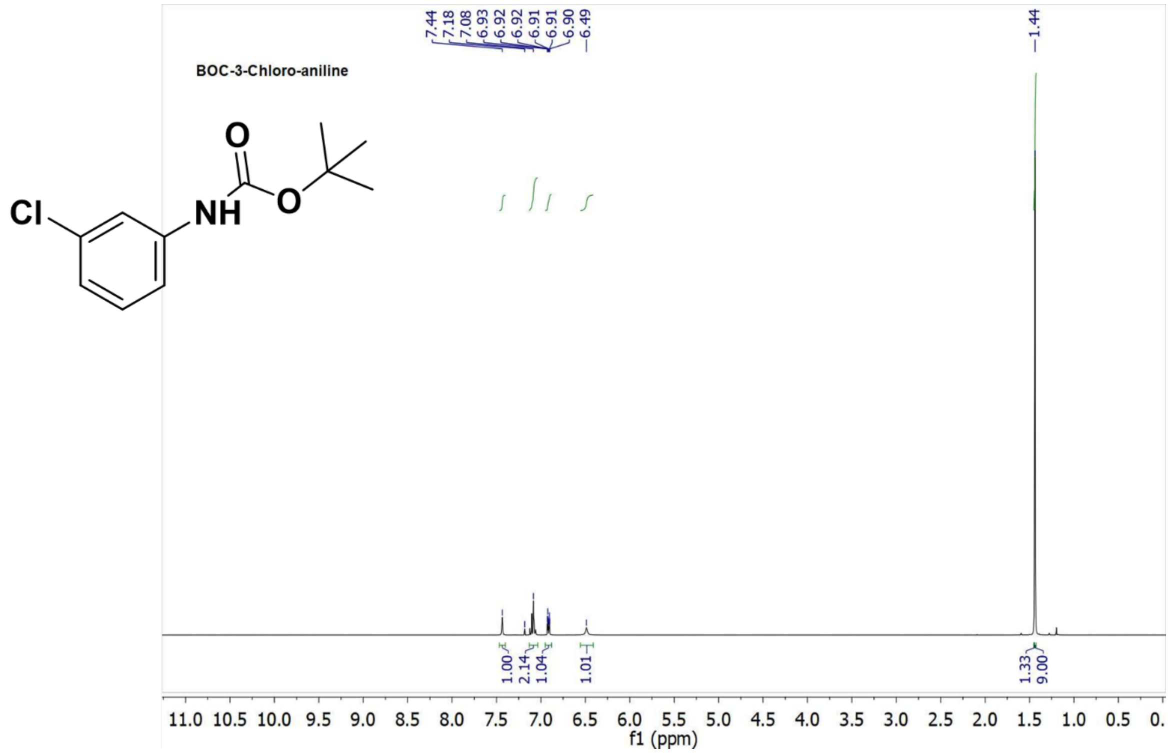

Figure S5: ^{1}H NMR spectrum of *tert*-butyl *N*-(3-chlorophenyl)carbamate (Entry 3a) in $CDCl_3$

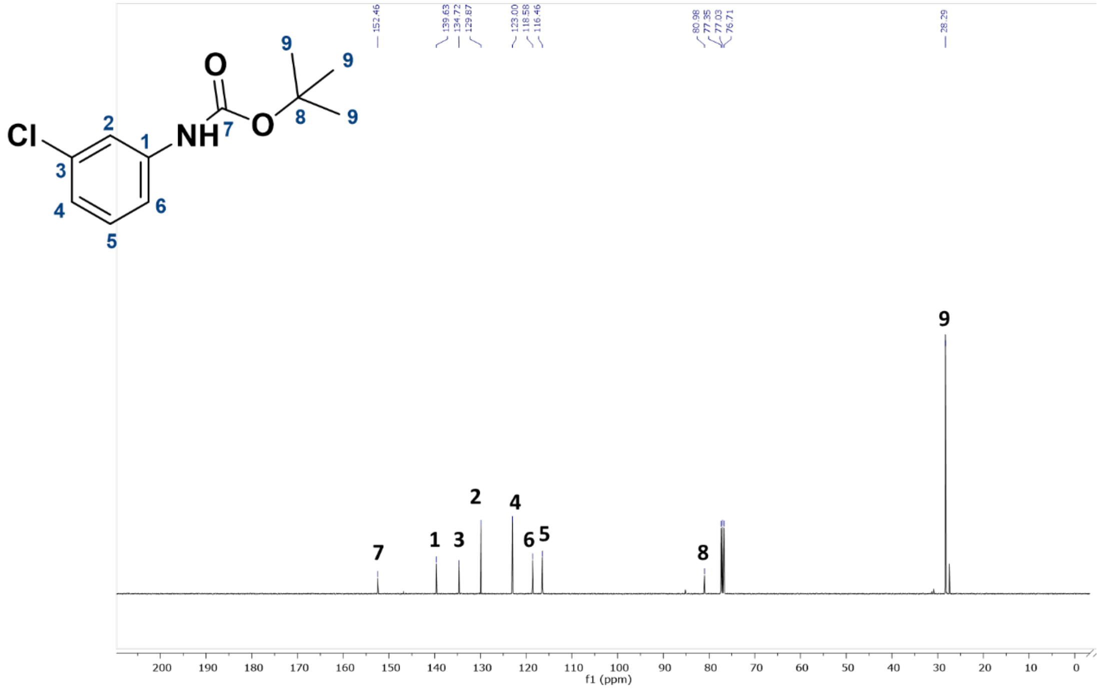

Figure S6: ^{13}C NMR spectrum of *tert*-butyl *N*-(3-chlorophenyl)carbamate (Entry 3a) in $CDCl_3$

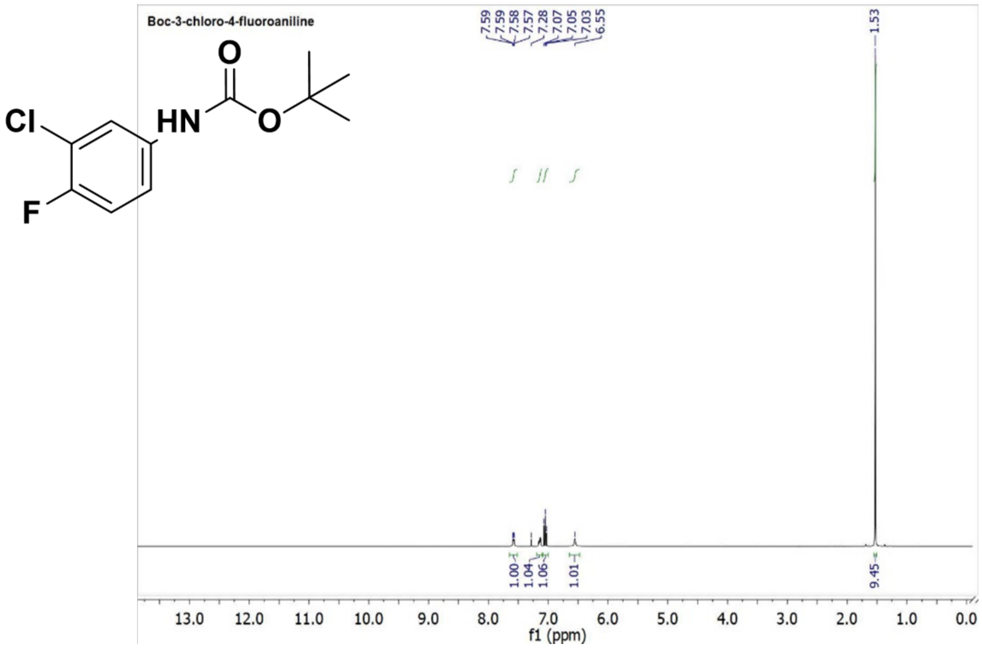

Figure S7: ^{1}H NMR spectrum of *tert*-butyl *N*-(3-chloro-4-fluorophenyl)carbamate (Entry 4a) in $CDCl_3$

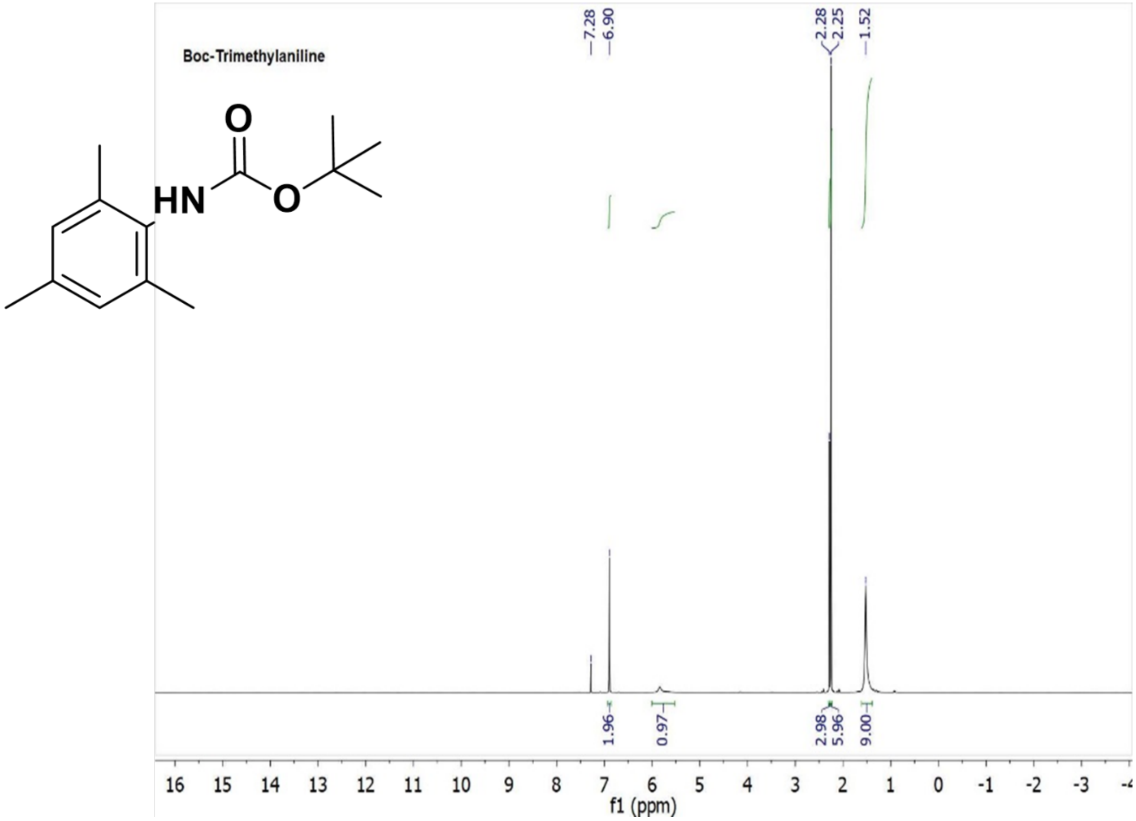

Figure S8: ^{1}H NMR spectrum of *tert*-butyl *N*-(2,4,6-trimethylphenyl)carbamate (Entry 5a) in $CDCl_3$

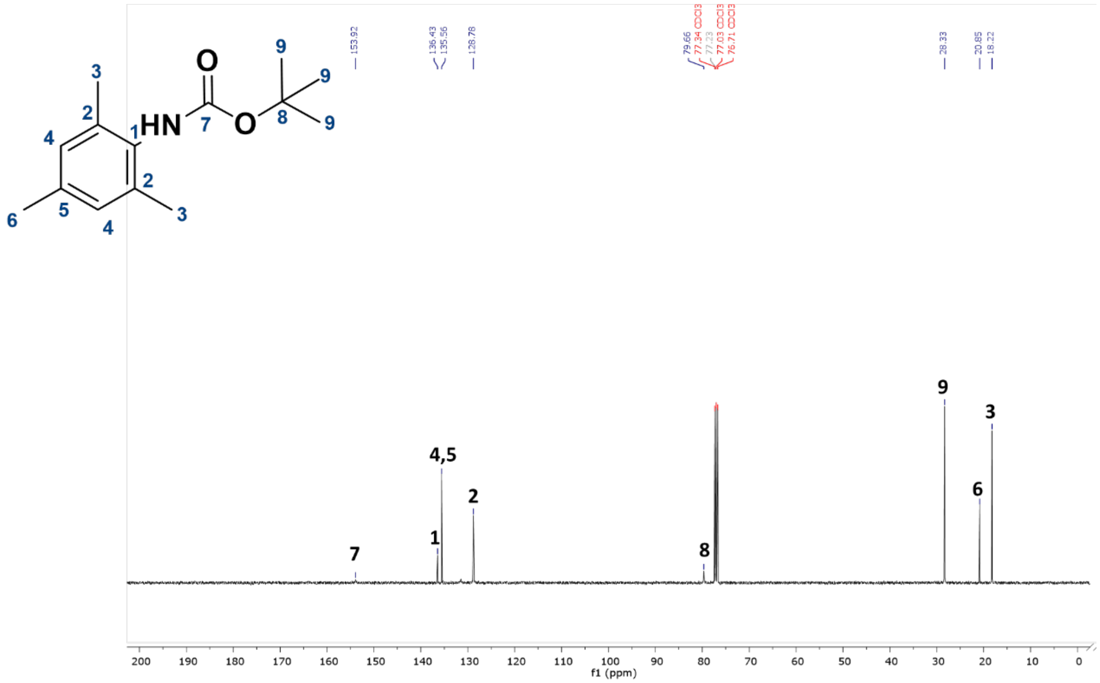

Figure S9: ^{13}C NMR spectrum of *tert*-butyl *N*-(2,4,6-trimethylphenyl)carbamate (Entry 5a) in $CDCl_3$

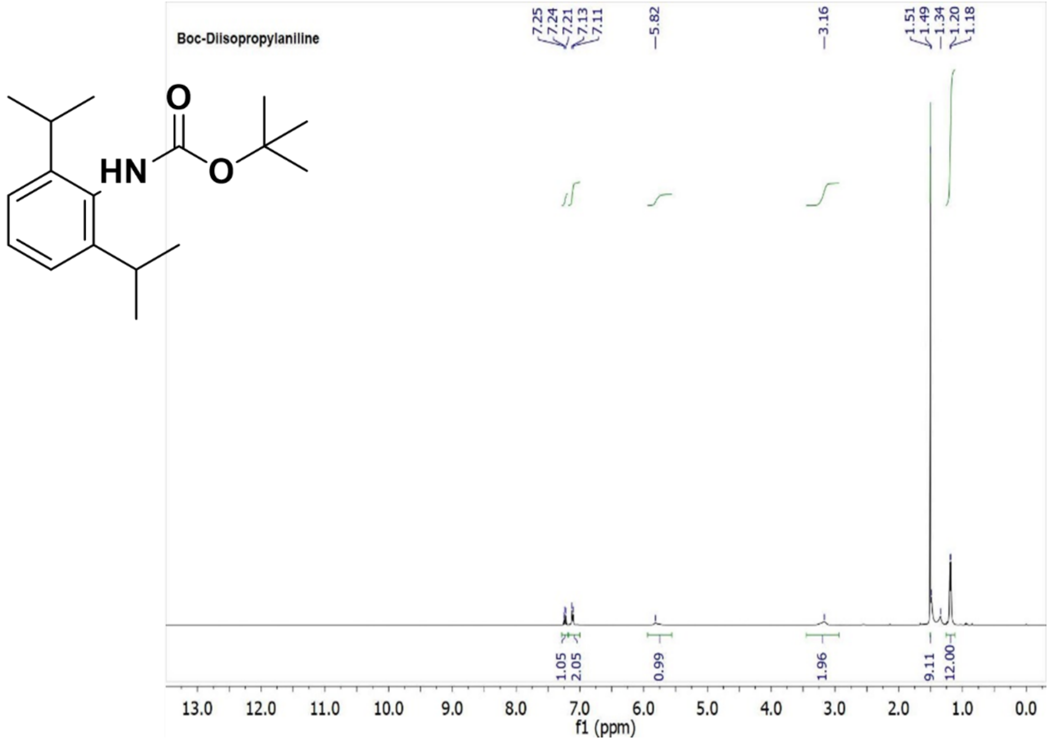

Figure S10: ^{1}H NMR spectrum of *tert*-butyl *N*-(2,6-diisopropylphenyl)carbamate (Entry 6a) in $CDCl_3$

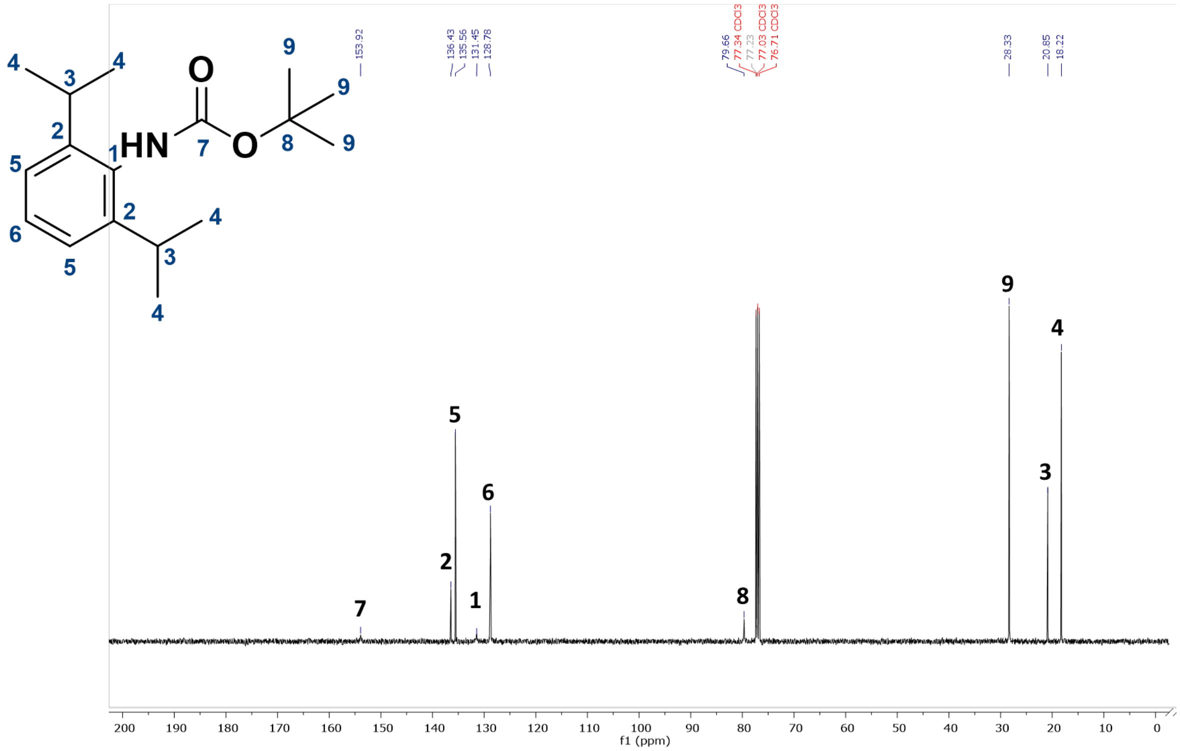

Figure S11: ^{13}C NMR spectrum of *tert*-butyl *N*-(2,6-diisopropylphenyl)carbamate (Entry 6a) in $CDCl_3$

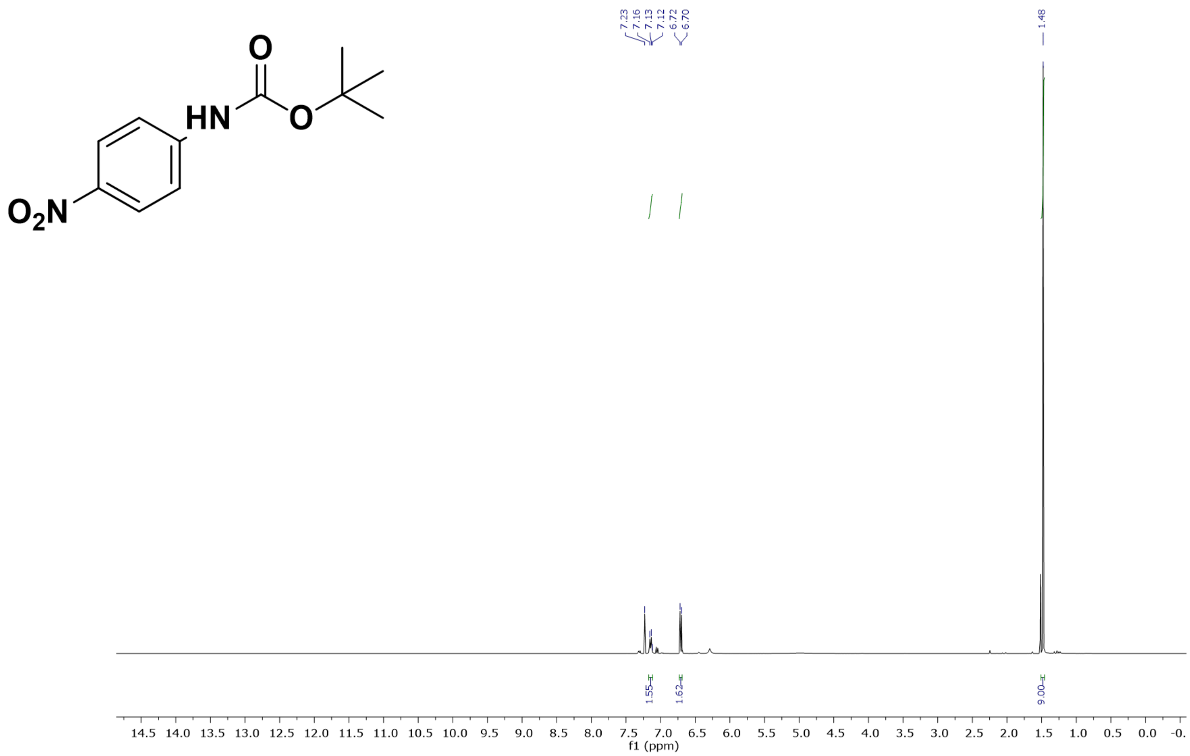

Figure S12: ^{1}H NMR spectrum of *tert*-butyl *N*-(4-nitrophenyl)carbamate (Entry 7a) in $CDCl_3$

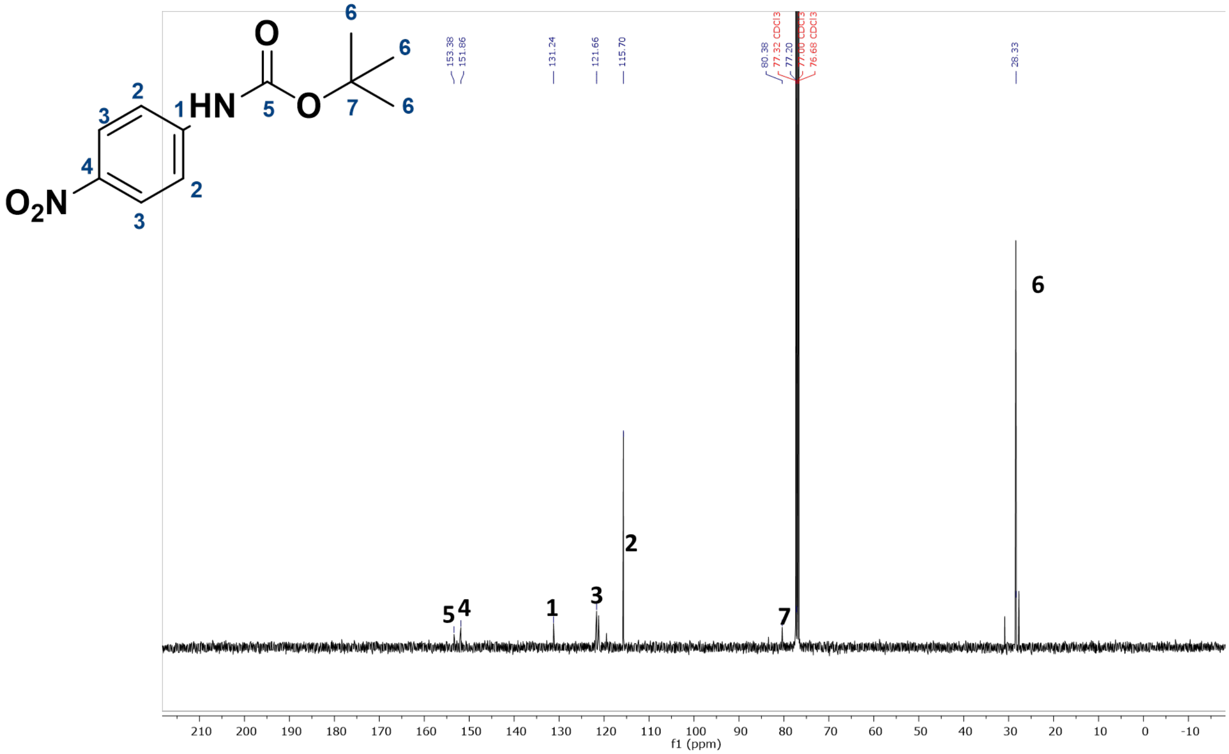

Figure S13: ^{13}C NMR spectrum of *tert*-butyl *N*-(4-nitrophenyl)carbamate (Entry 7a) in $CDCl_3$

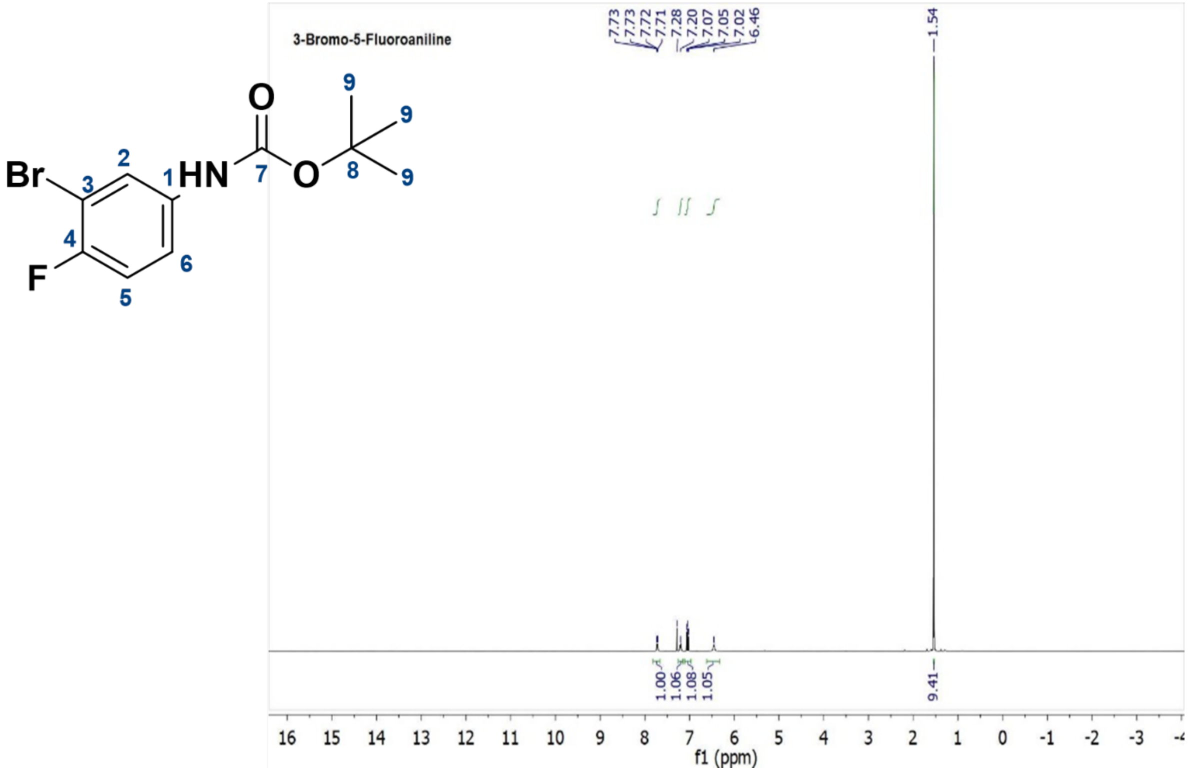

Figure S14: ^{1}H NMR spectrum of *tert*-butyl *N*-(3-bromo-4-fluorophenyl)carbamate (Entry 8a) in $CDCl_3$

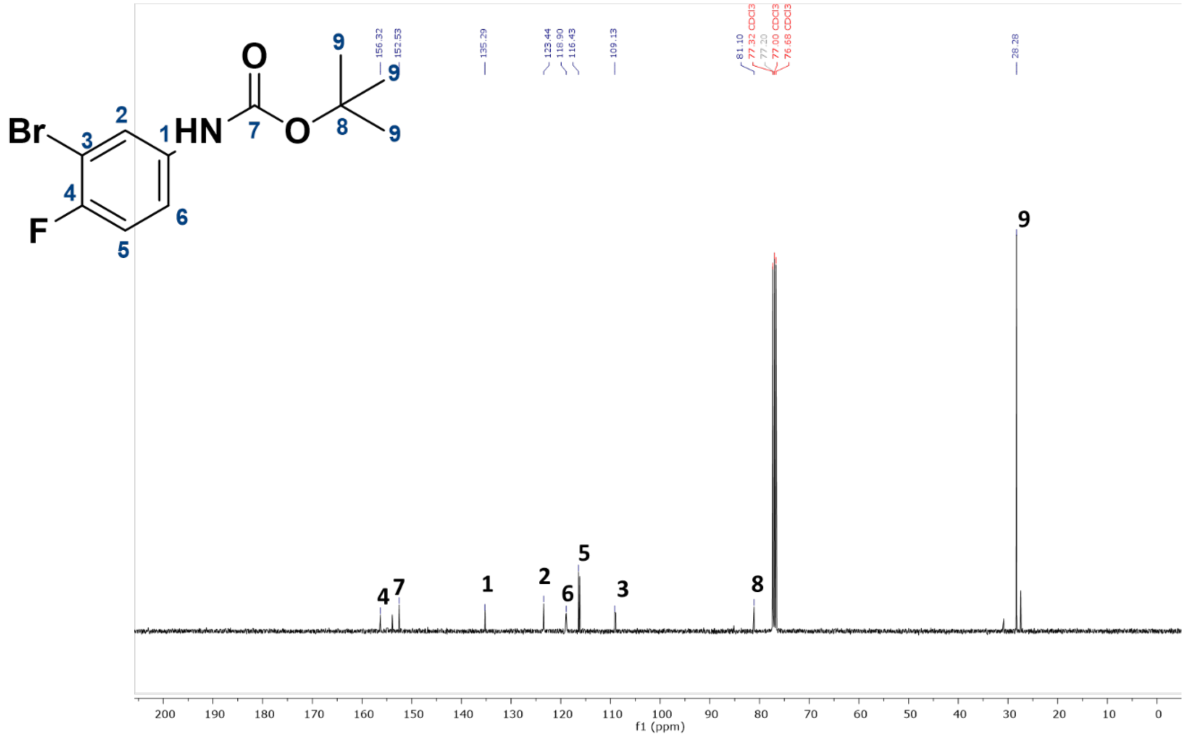

Figure S15: ^{13}C NMR spectrum of *tert*-butyl *N*-(3-bromo-4-fluorophenyl)carbamate (Entry 8a) in $CDCl_3$

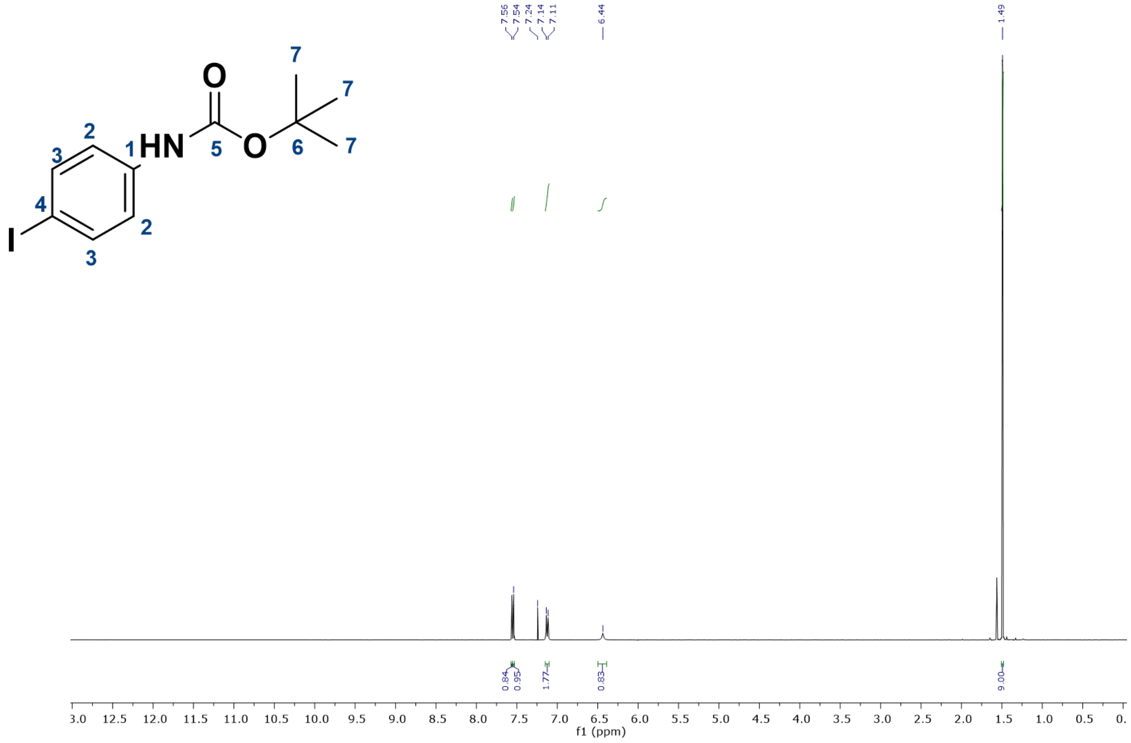

Figure S16: ^{1}H NMR spectrum of *tert*-Butyl *N*-(4-iodophenyl)carbamate (Entry 9a) in $CDCl_3$

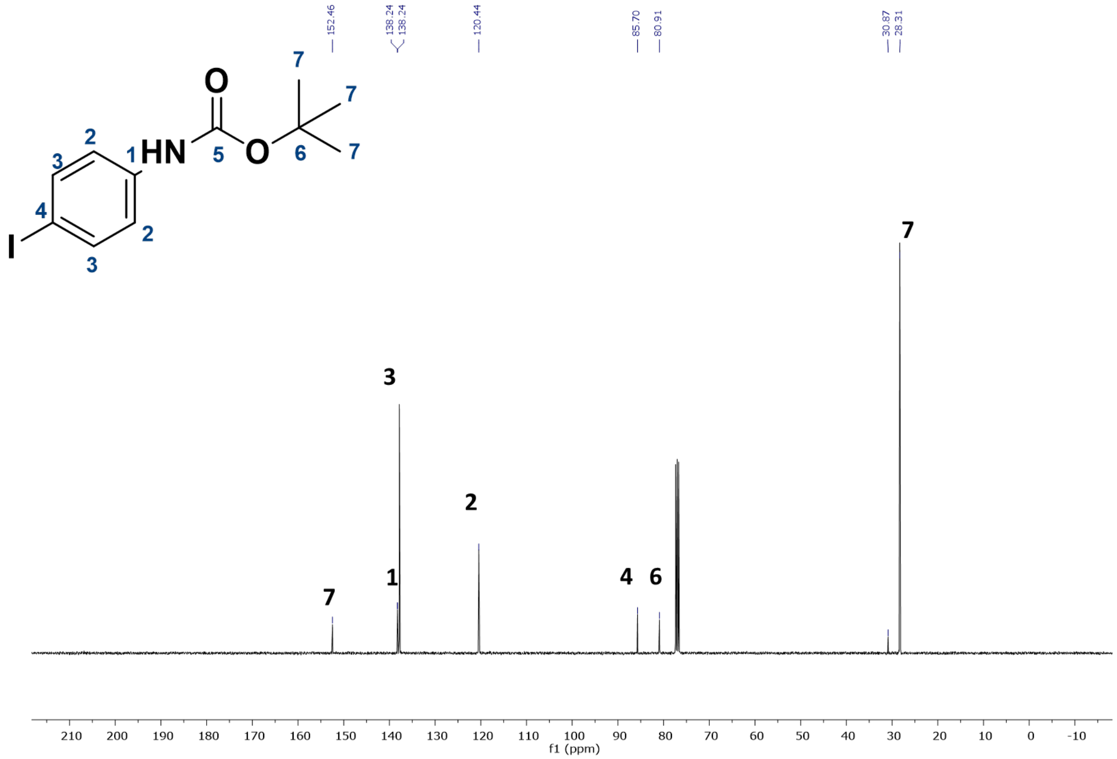

Figure S17: ^{13}C NMR spectrum of *tert*-Butyl *N*- (4-iodophenyl)carbamate (Entry 9a) in CDCl*3*

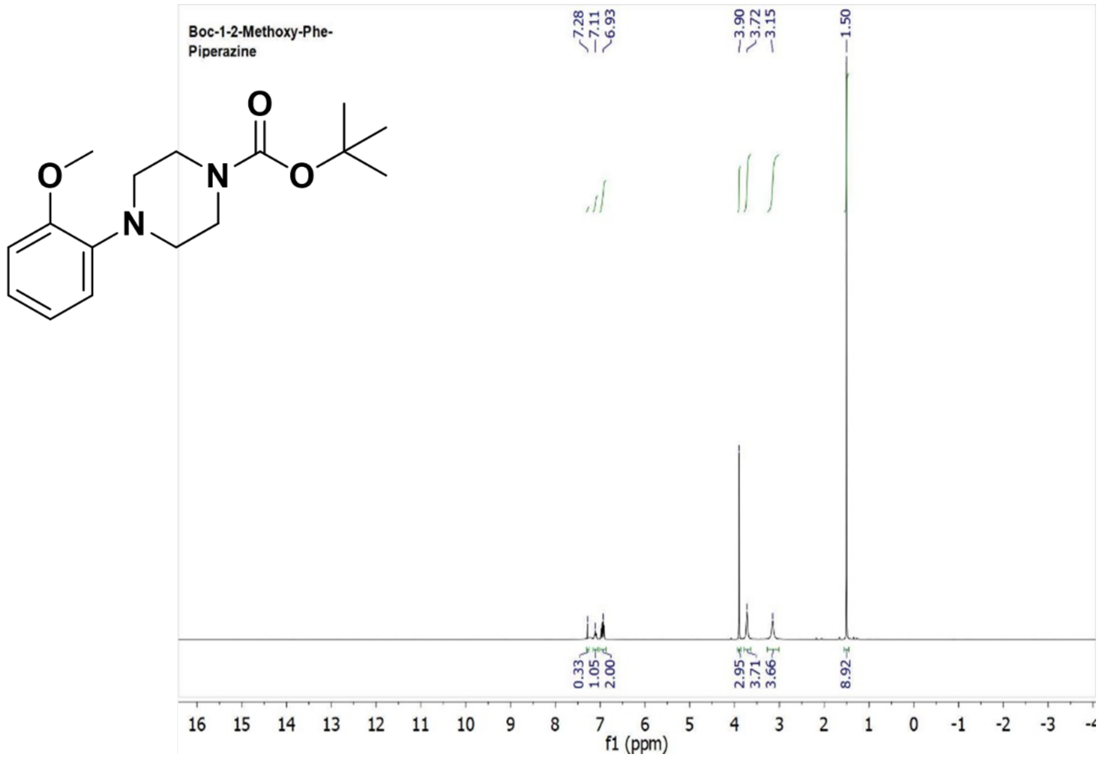

Figure S18: ^{1}H NMR spectrum of *tert*-butyl *N*-(N-(2-Methoxyphenyl)piperazine)carbamate(Entry 10a) in CDCl*3*

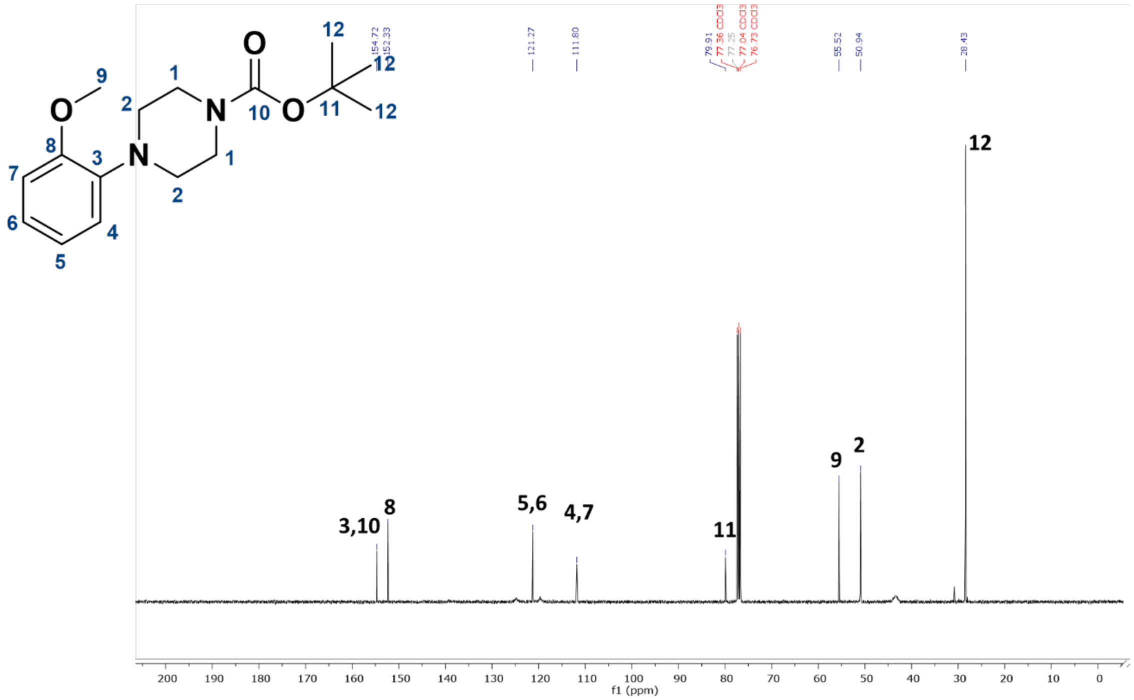

Figure S19: ^{13}C NMR spectrum of *tert*-butyl *N*-(N-(2-Methoxyphenyl)piperazine)carbamate (Entry 10a) in $CDCl_3$

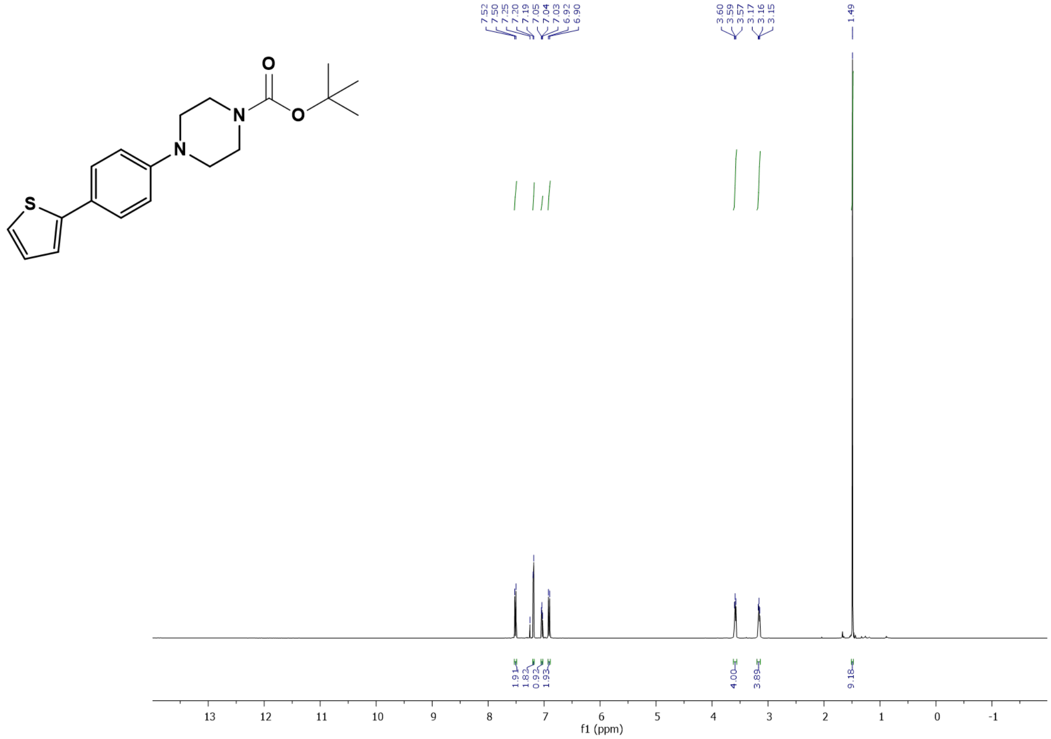

Figure S20: ^{1}H NMR spectrum of *tert*-butyl *N*-(N-(4-thiophene-phenyl)piperazine)carbamate (Entry 11a) in $CDCl_3$

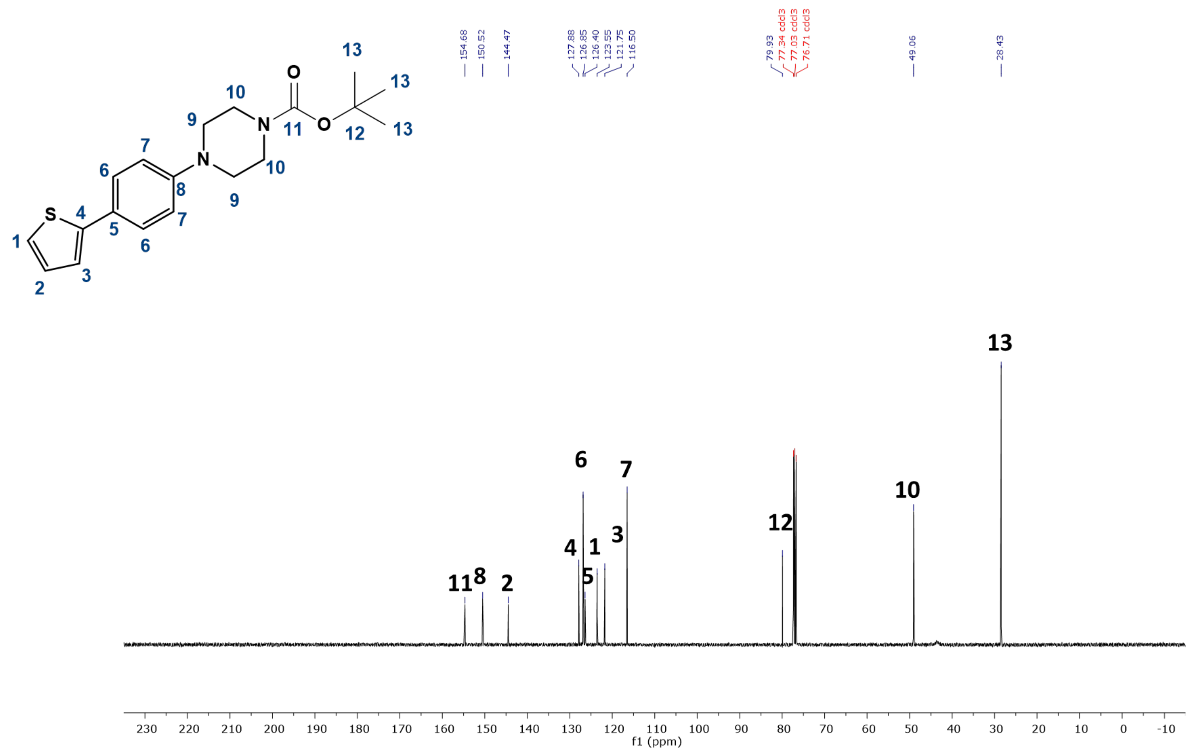

Figure S21: ^{13}C NMR spectrum of *tert*-butyl *N*-(N-(4-thiophene-phenyl)piperazine)carbamate (Entry 11a) in $CDCl_3$

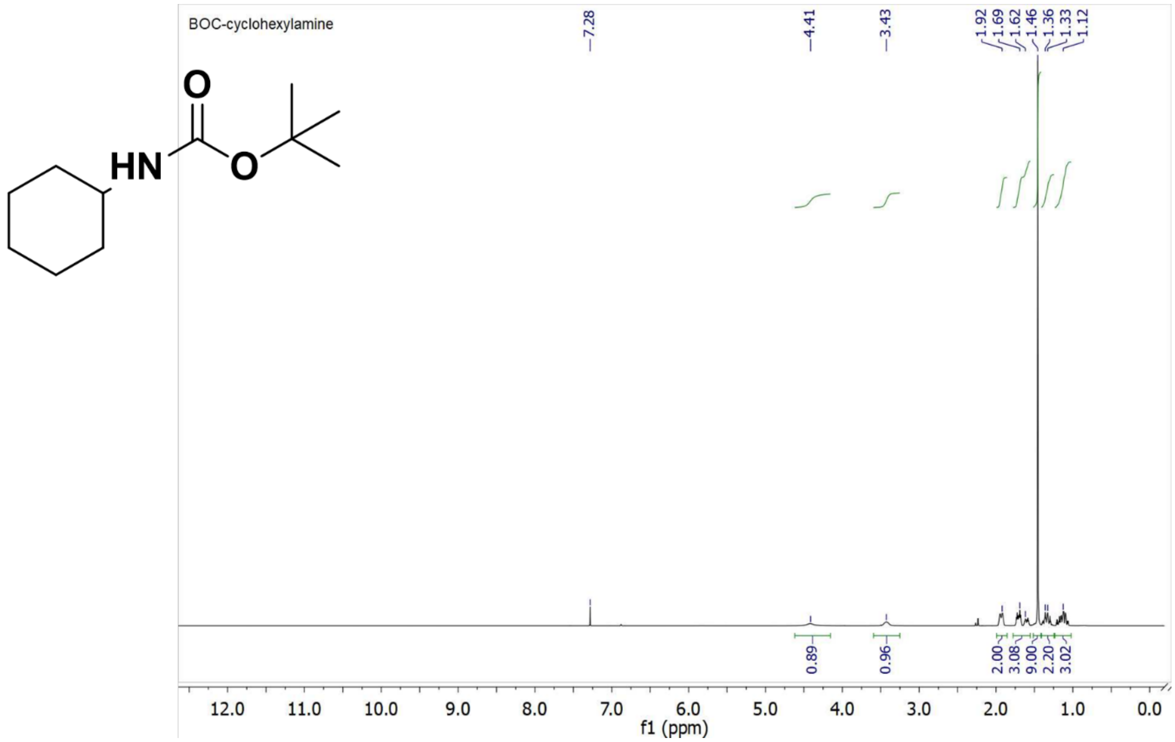

Figure S22: ^{1}H NMR spectrum of *tert*-butyl *N*-(cyclohexyl)carbamate (Entry 12a) in $CDCl_3$

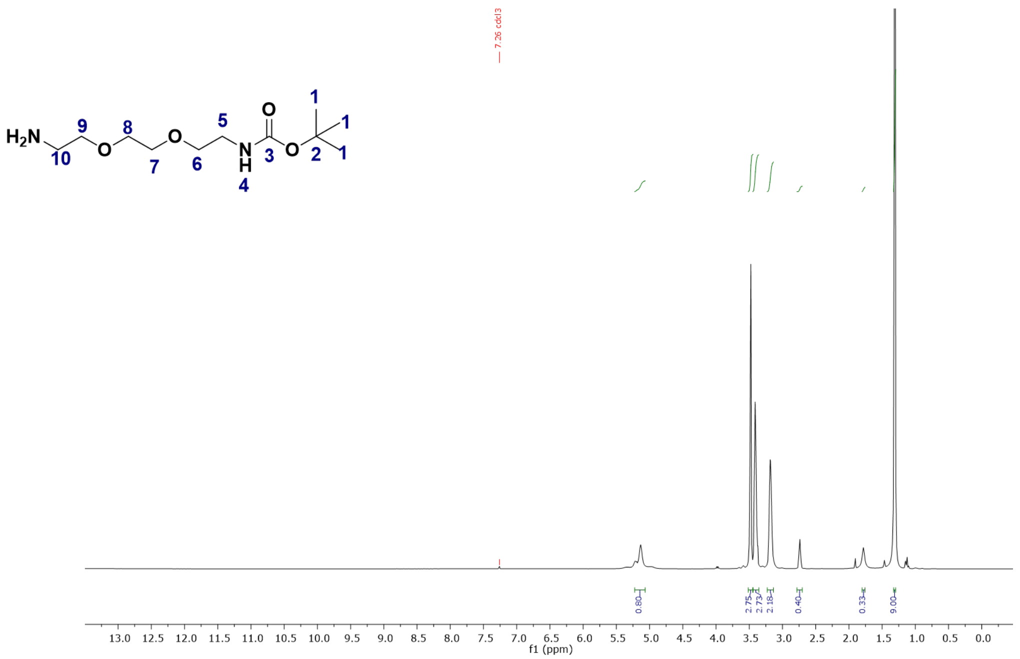

Figure S23: ^{1}H NMR spectrum of *tert*-butyl *N*-(2-[2-(2-aminoethoxy)ethoxy]ethanamine)carbamate (Entry 13a) in $CDCl_3$

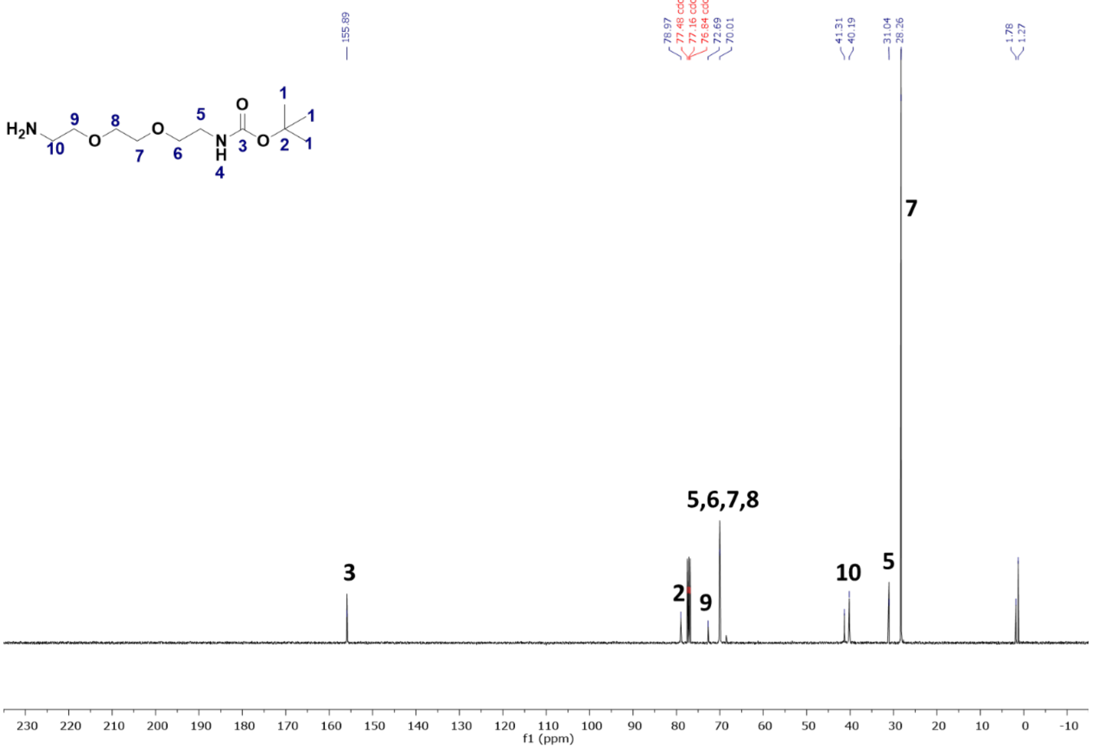

Figure S24: ^{13}C NMR spectrum of *tert*-butyl *N*-(2-[2-(2-aminoethoxy)ethoxy]ethanamine)carbamate (Entry 13a) in $CDCl_3$

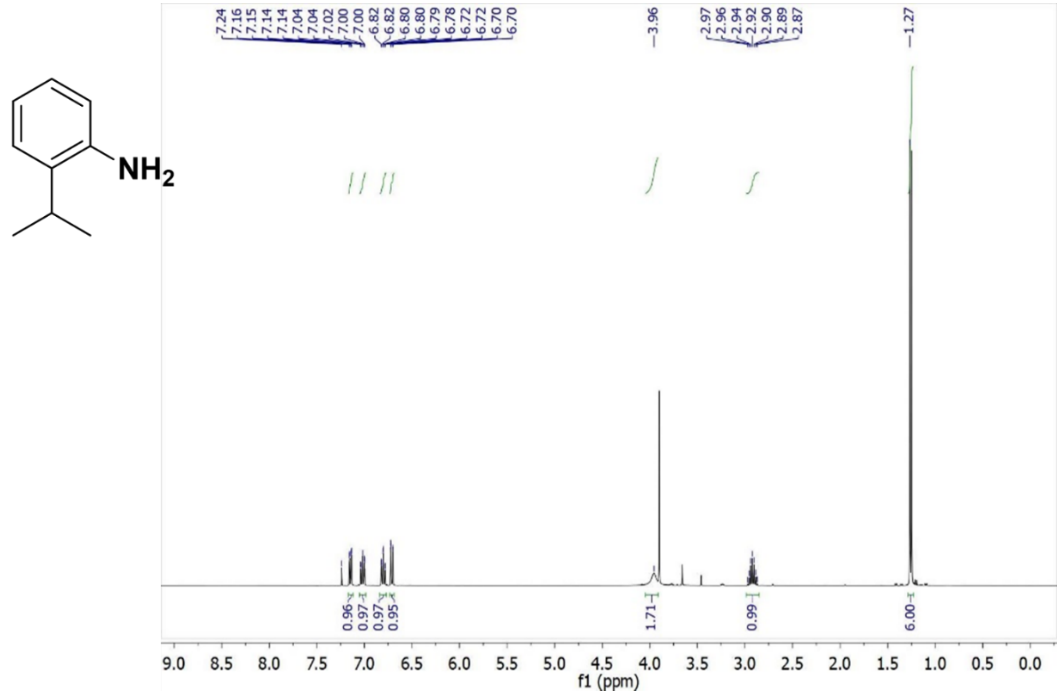

Figure S25: ^{1}H NMR spectrum of 2-isopropylaniline (Entry 1b) in $CDCl_3$

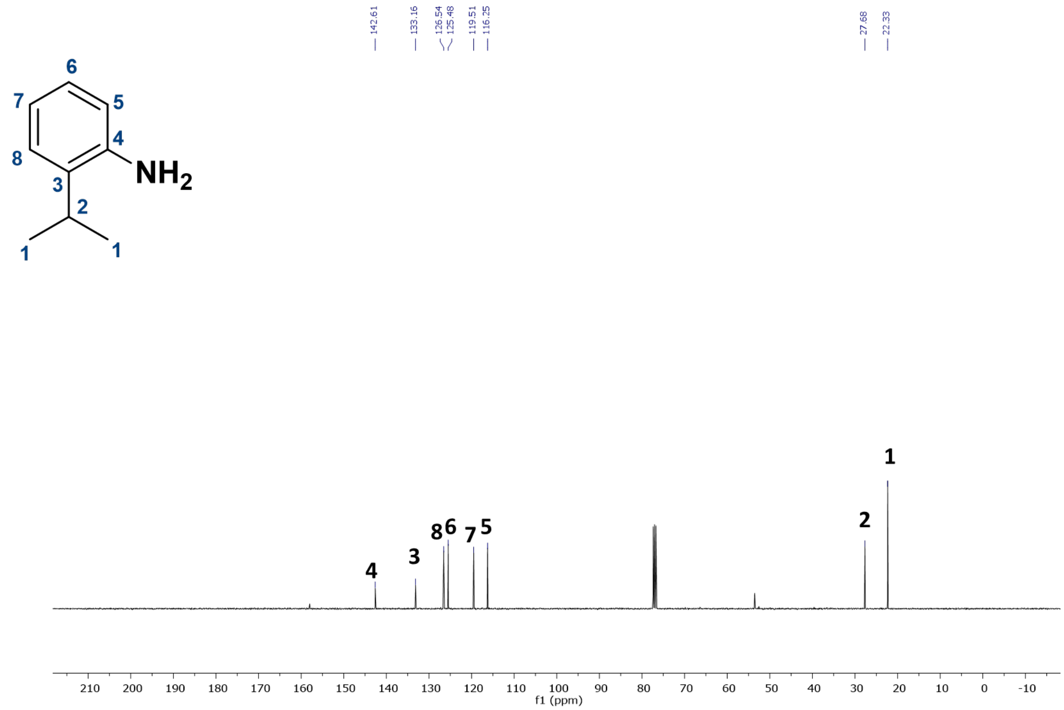

Figure S26: ^{13}C NMR spectrum of 2-isopropylaniline (Entry 1b) in $CDCl_3$

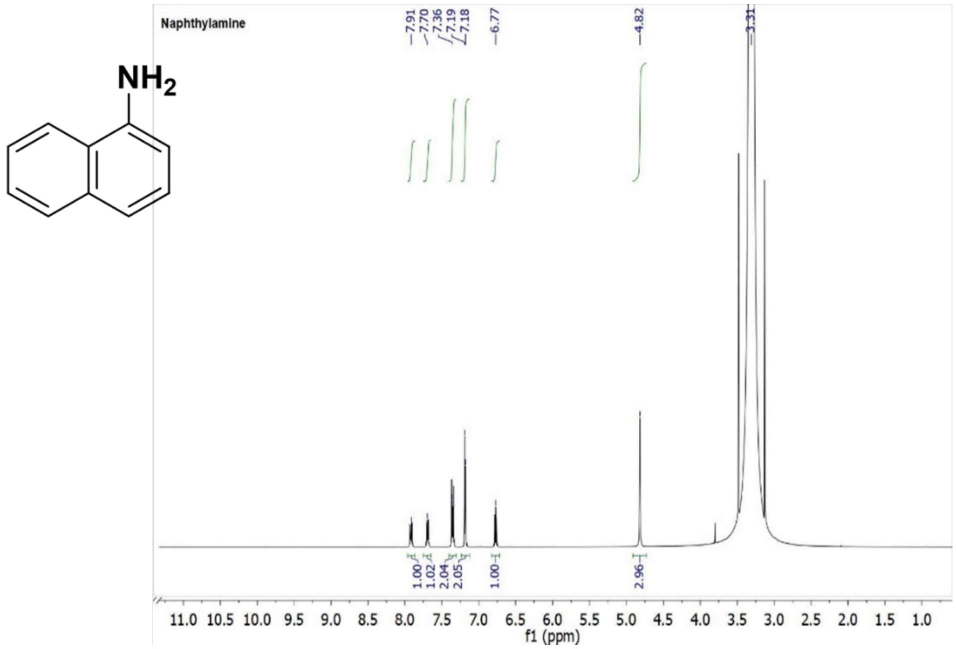

Figure S27: ^{1}H NMR spectrum of Naphthylamine (Entry 2b) in MeOD

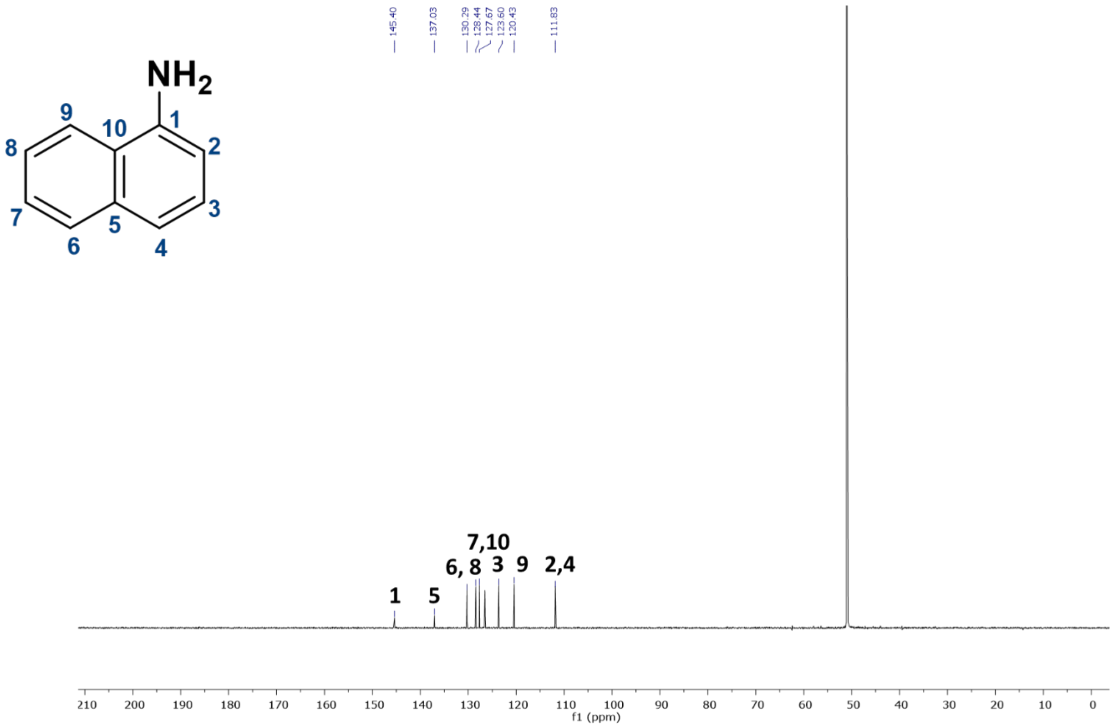

Figure S28: ^{13}C NMR spectrum of Naphthylamine (Entry 2b) in MeOD

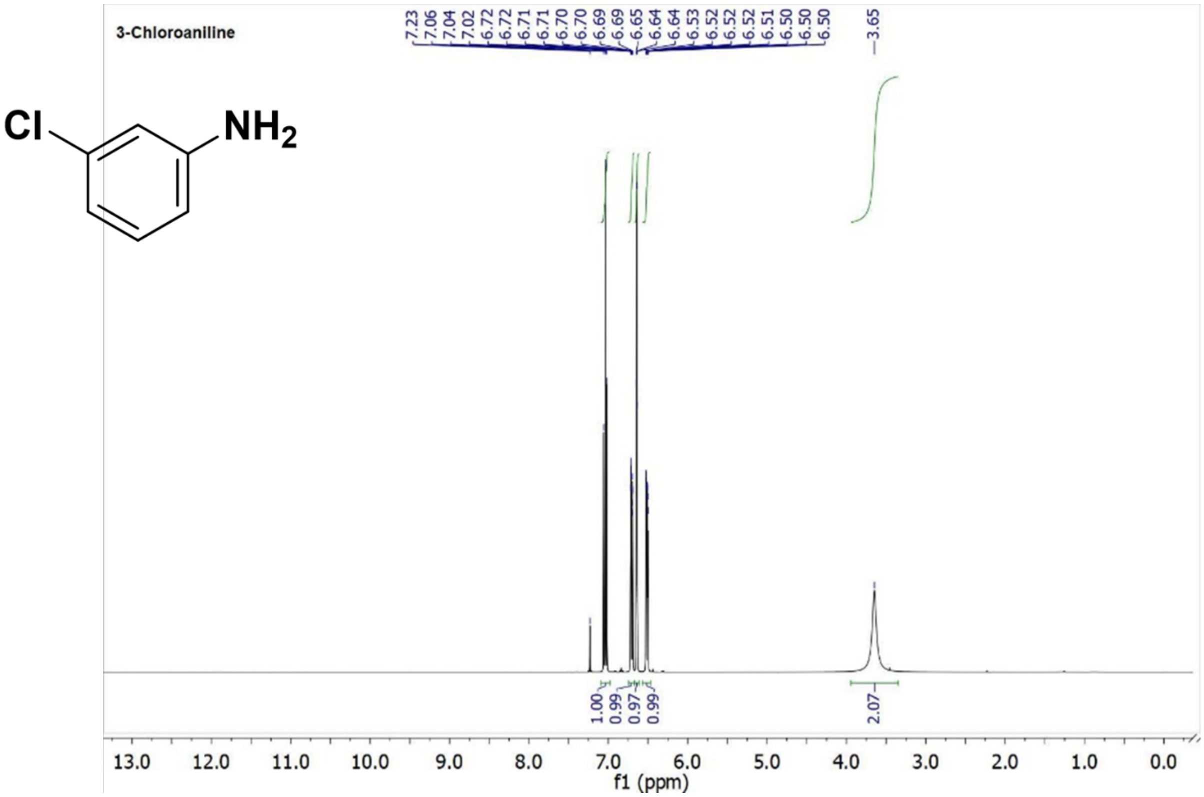

Figure S29: ^{1}H NMR spectrum of 3-chloroaniline (Entry 3b) in $CDCl_3$

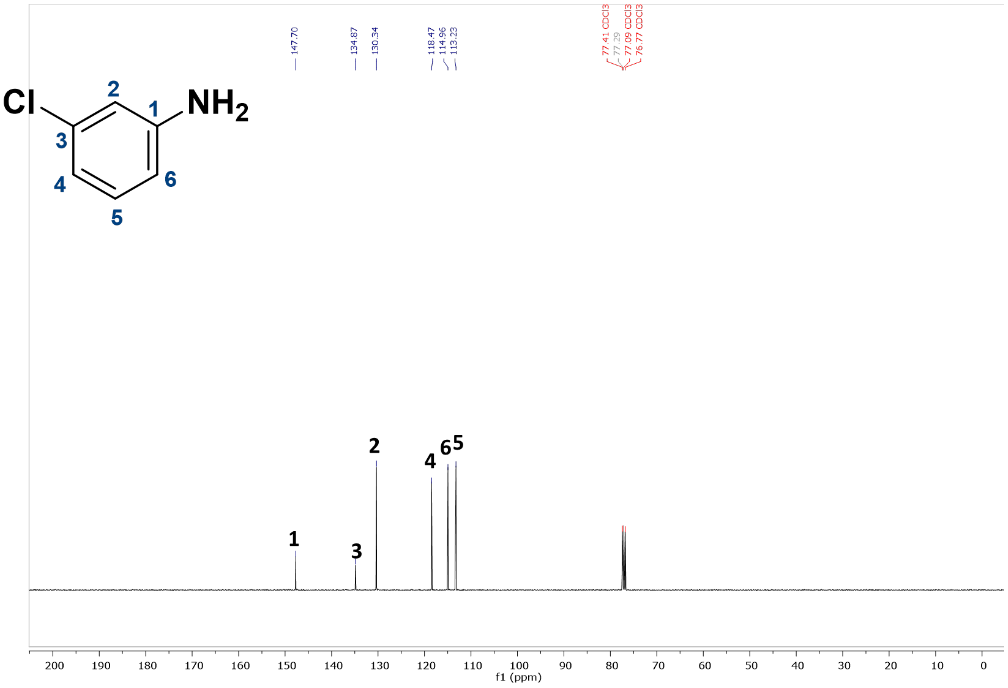

Figure S30: ^{13}C NMR spectrum of 3-chloroaniline (Entry 3b) in $CDCl_3$

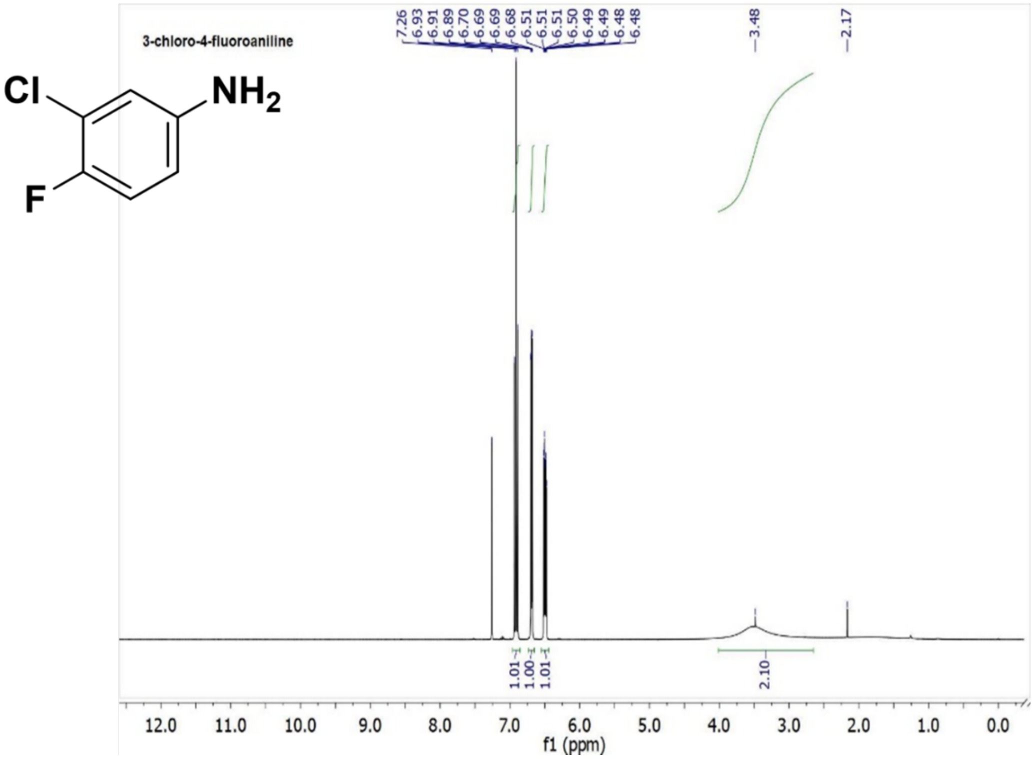

Figure S31: ^{1}H NMR spectrum of 3-chloro-4-flouroaniline (Entry 4b) in $CDCl_3$

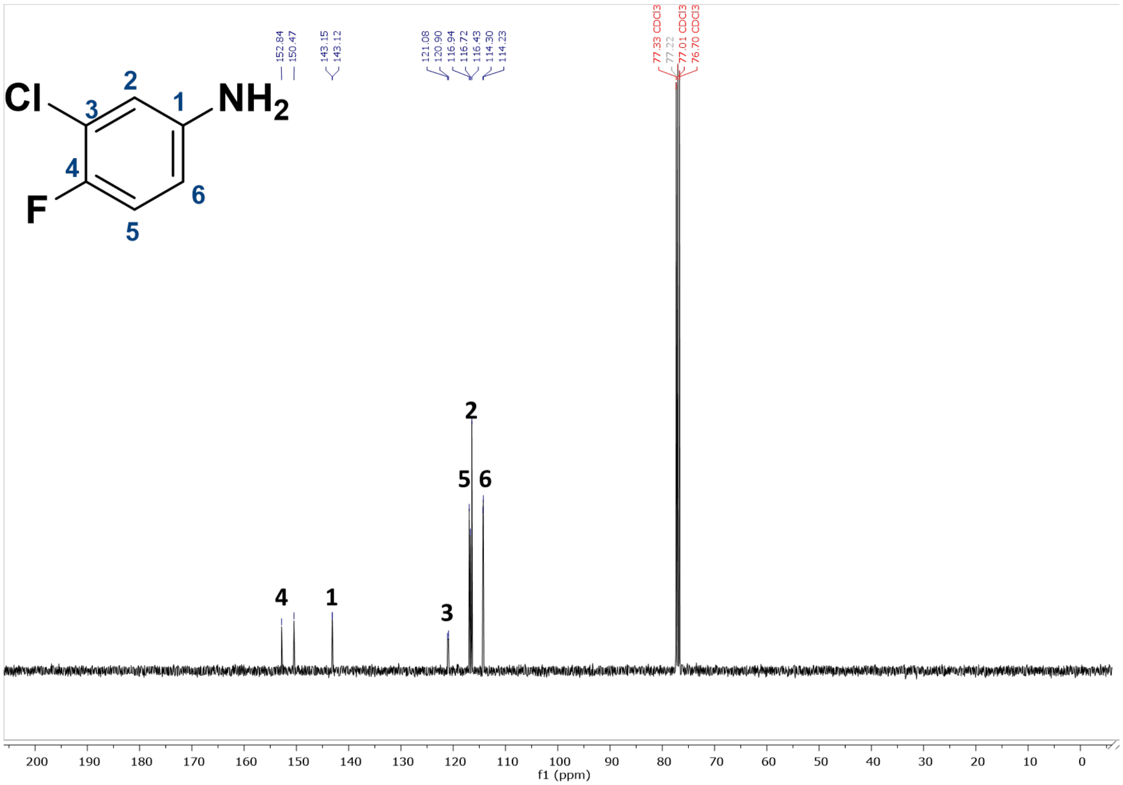

Figure S32: ^{13}C NMR spectrum of 3-chloro-4-flouroaniline (Entry 4b) in $CDCl_3$

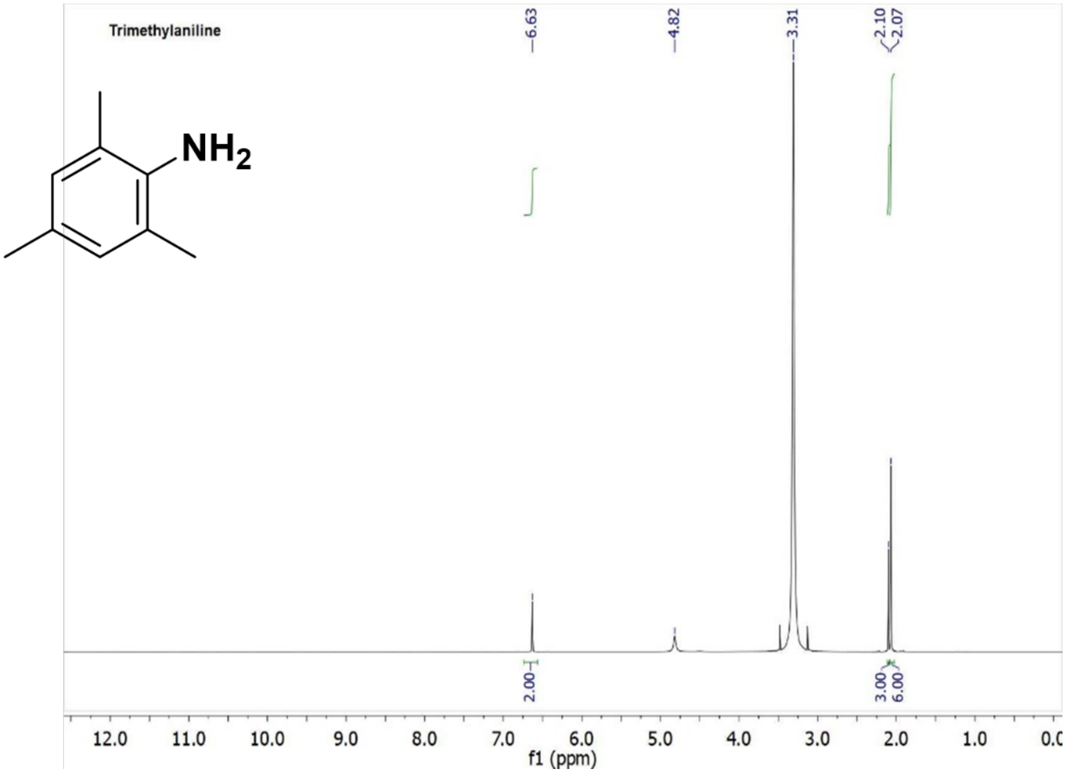

Figure S33: ^{1}H NMR spectrum of 2,4,6-trimethylaniline (Entry 5b) in $CDCl_3$

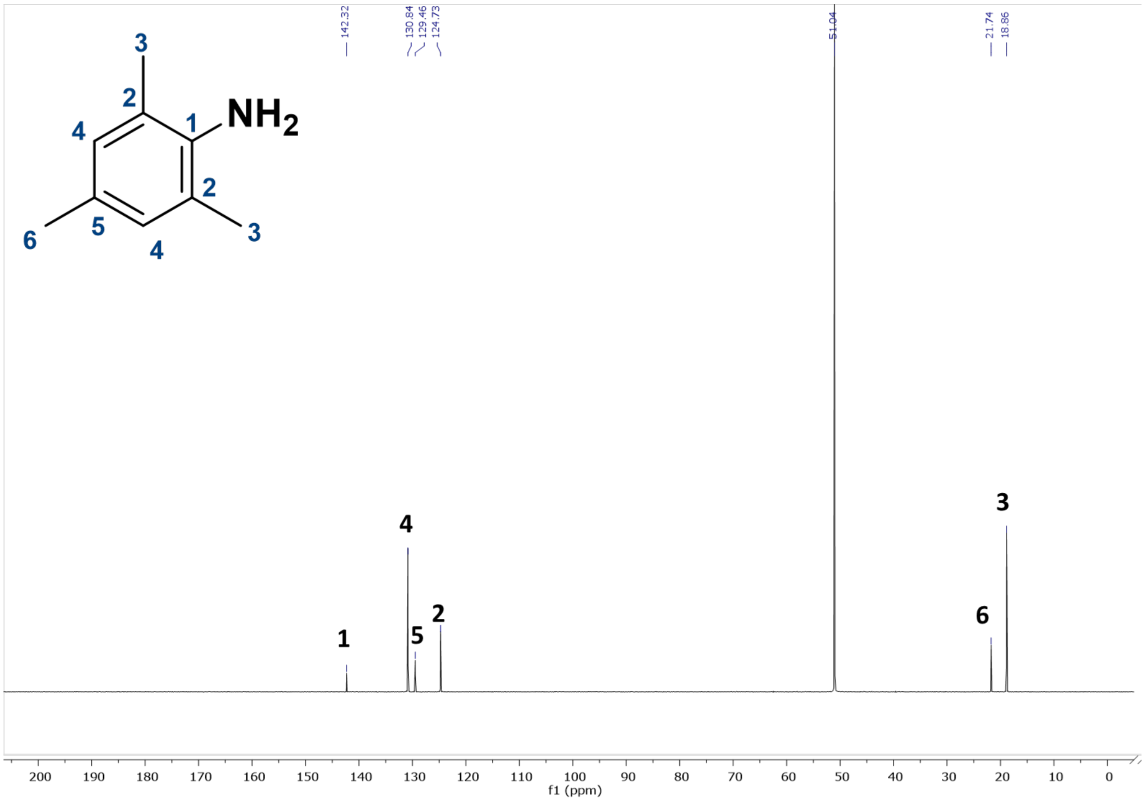

Figure S34: ^{13}C NMR spectrum of 2,4,6-trimethylaniline (Entry 5b) in $CDCl_3$

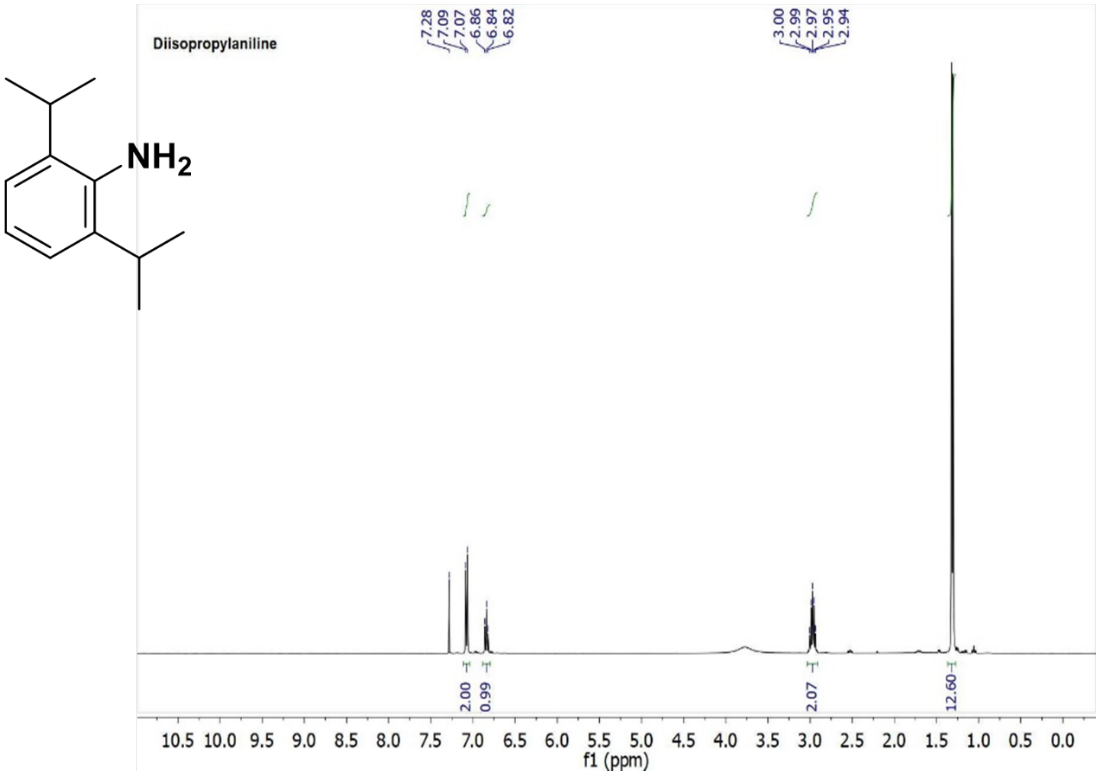

Figure S35: ^{1}H NMR spectrum of 2,6-diisopropylaniline (Entry 6b) in CDCl$_{3}$

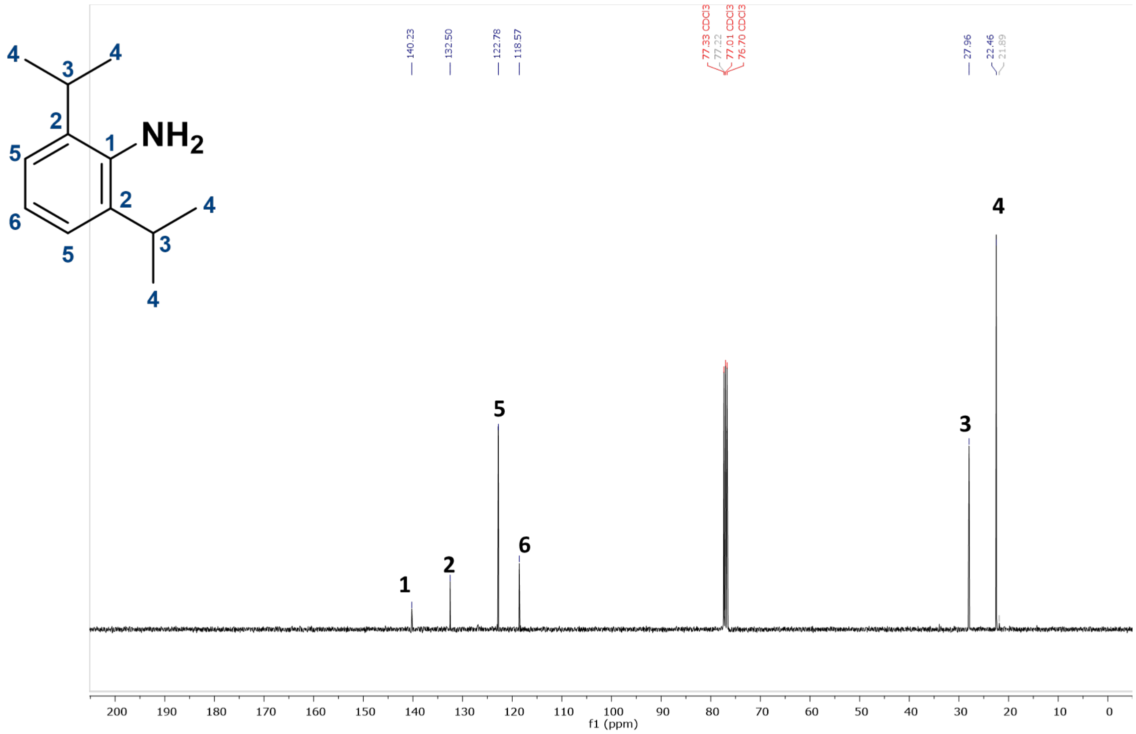

Figure S36: ^{13}C NMR spectrum of 2,6-diisopropylaniline (Entry 6b) in CDCl$_{3}$

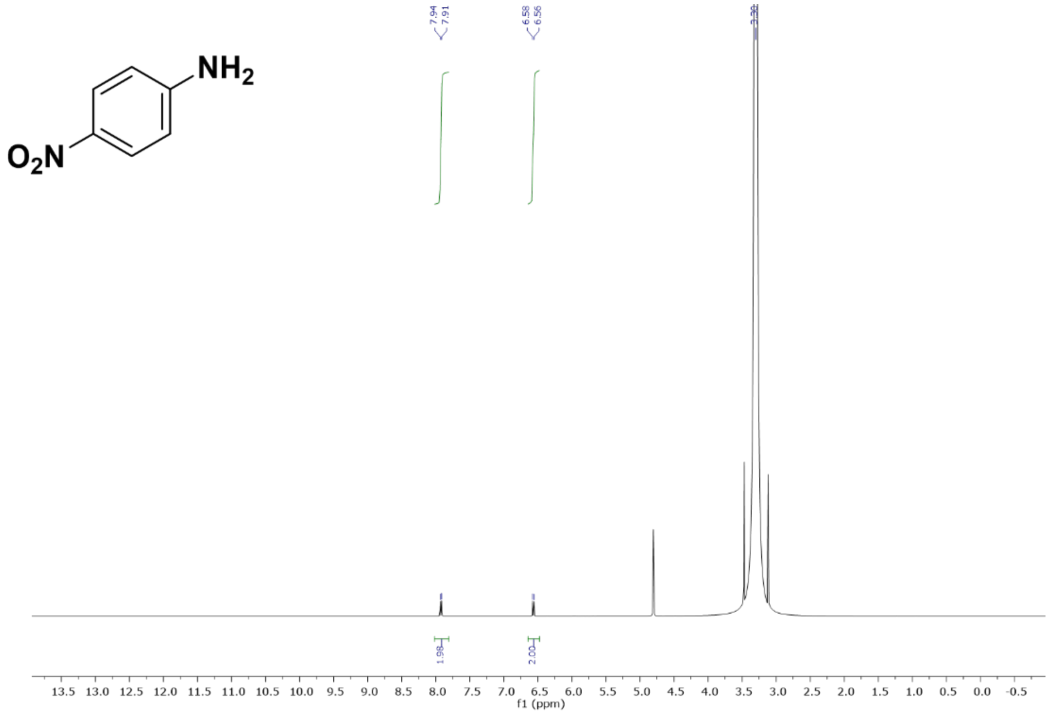

Figure S37: ^{1}H NMR spectrum of 4-Nitroaniline in $CDCl_3$ (Entry 7b)

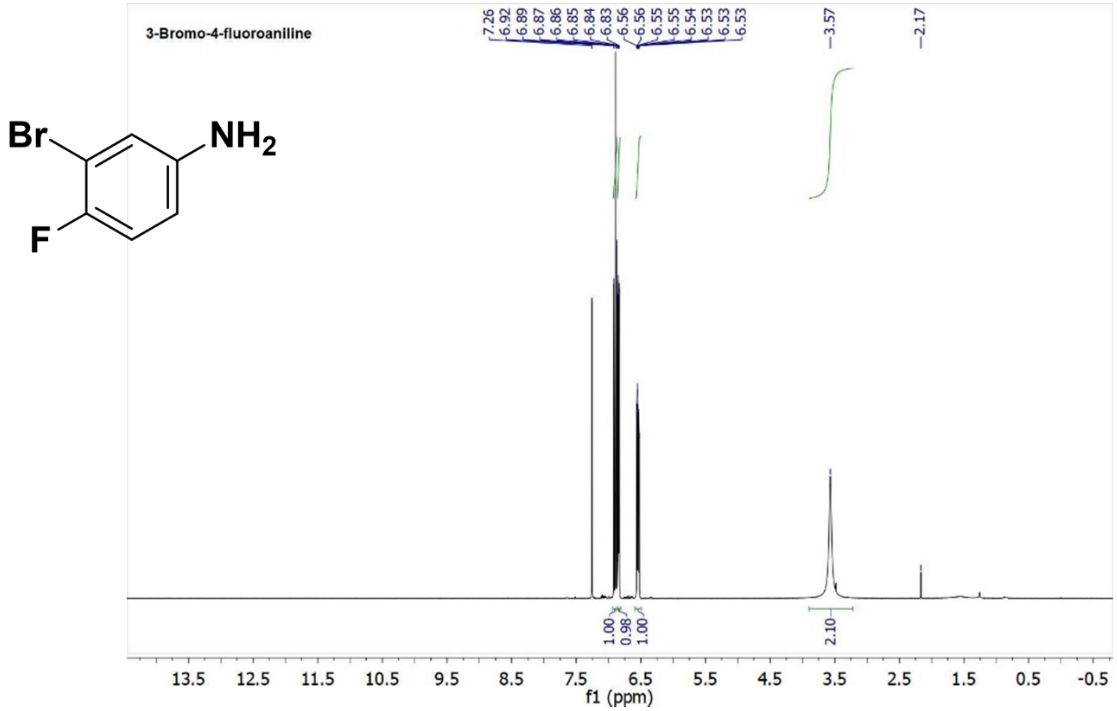

Figure S38: ^{1}H NMR spectrum of 3-bromo-4-fluoroaniline (Entry 8b) in $CDCl_3$

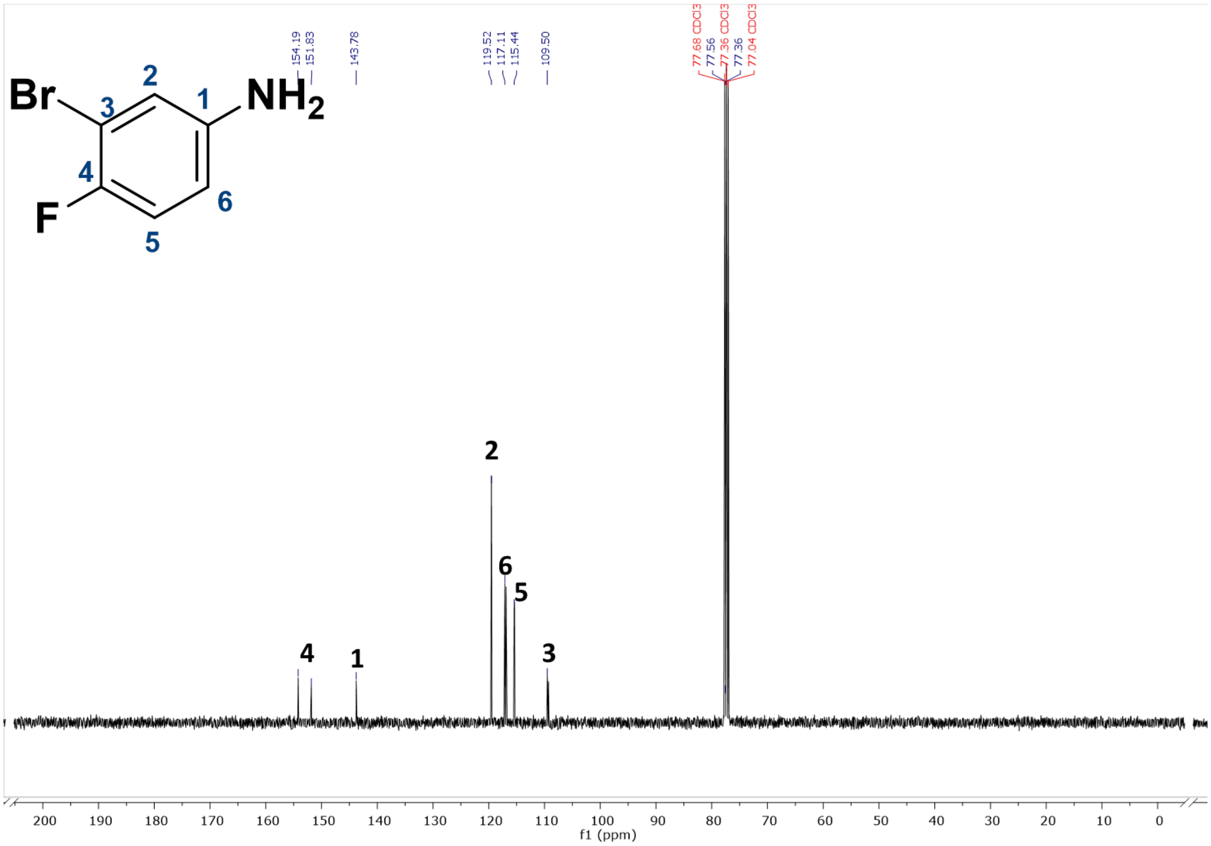

Figure S39: ^{13}C NMR spectrum of 3-bromo-4-fluoroaniline (Entry 8b) in $CDCl_3$

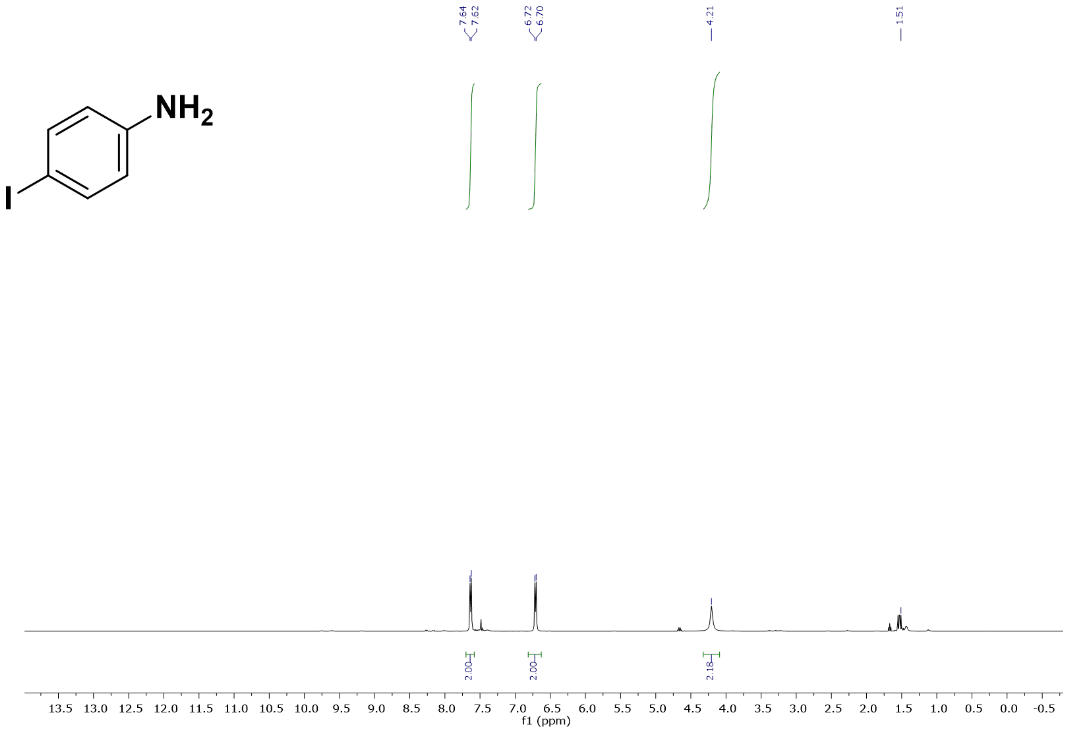

Figure S40: ^{1}H NMR spectrum of 4-iodoaniline (Entry 9b) in $CDCl_3$

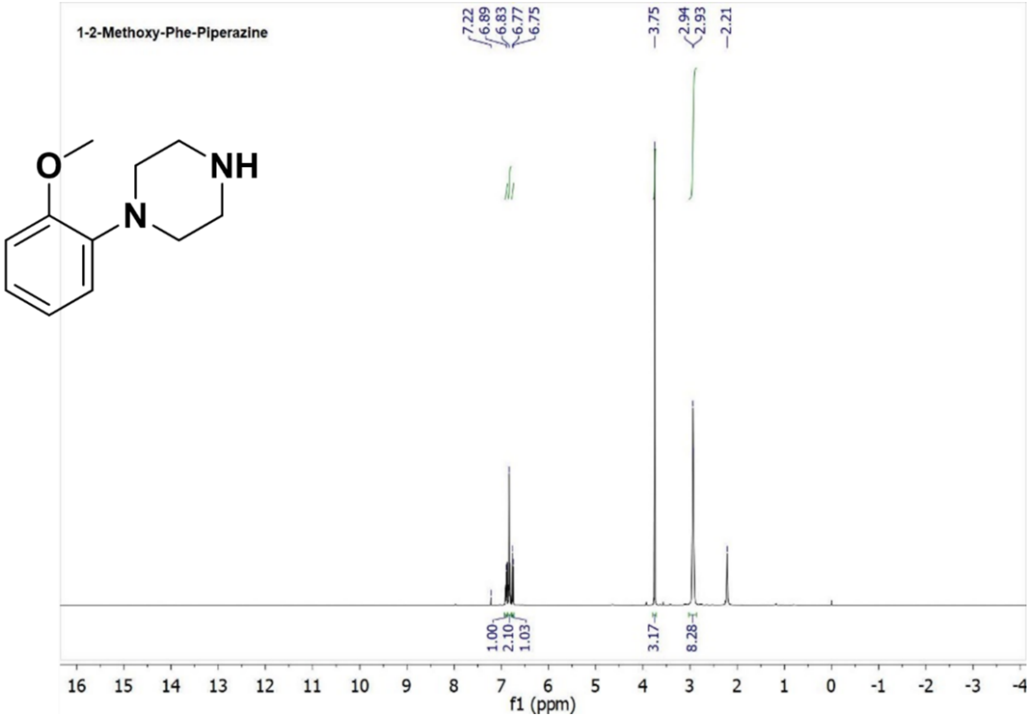

Figure S41: ^{1}H NMR spectrum of N-(2-Methoxyphenyl)piperazine (Entry 10b) in $CDCl_3$

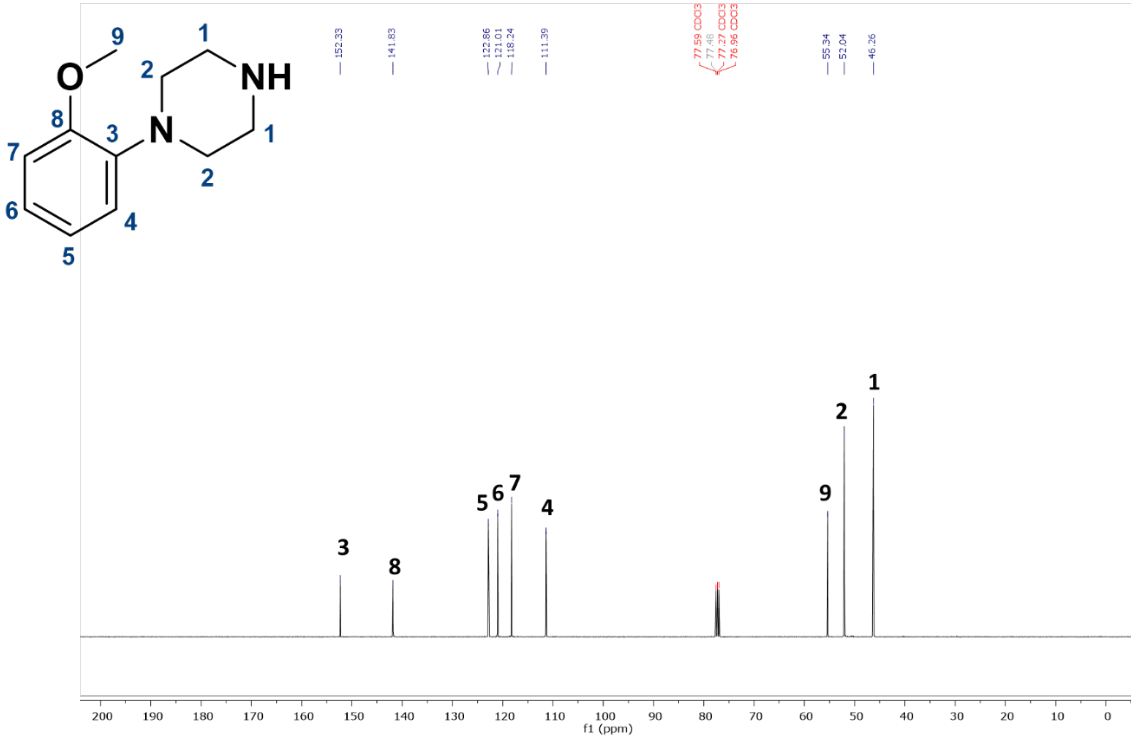

Figure S42: ^{13}C NMR spectrum of N-(2-Methoxyphenyl)piperazine (Entry 10b) in $CDCl_3$

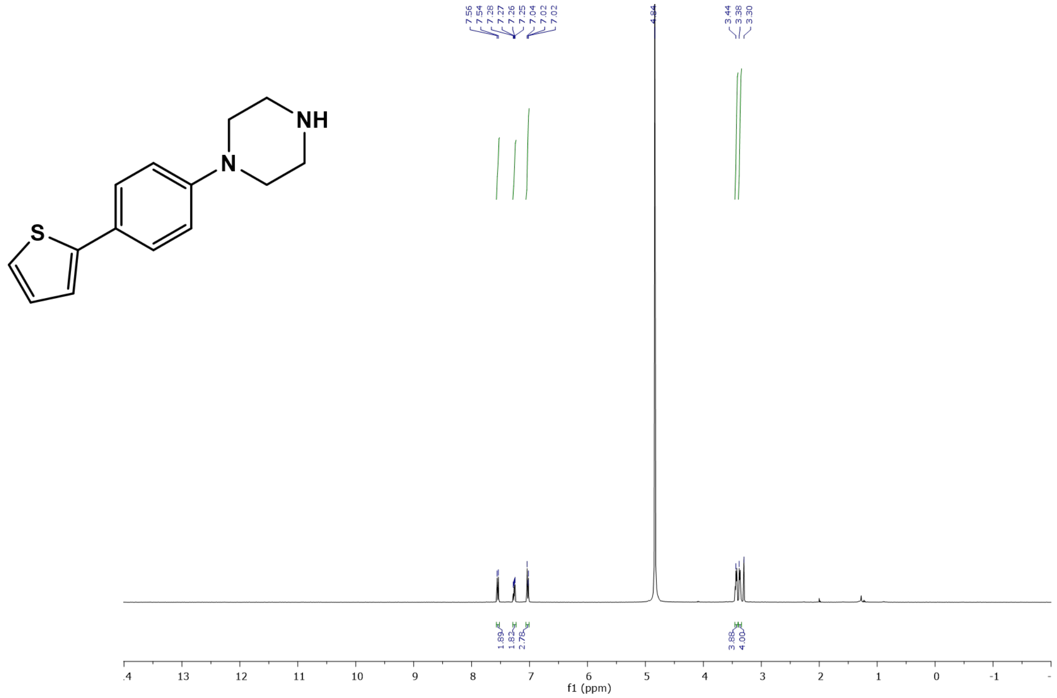

Figure S43: ^{1}H NMR spectrum of N-(4-thiophene-phenyl)piperazine (Entry 11b) in $CDCl_3$

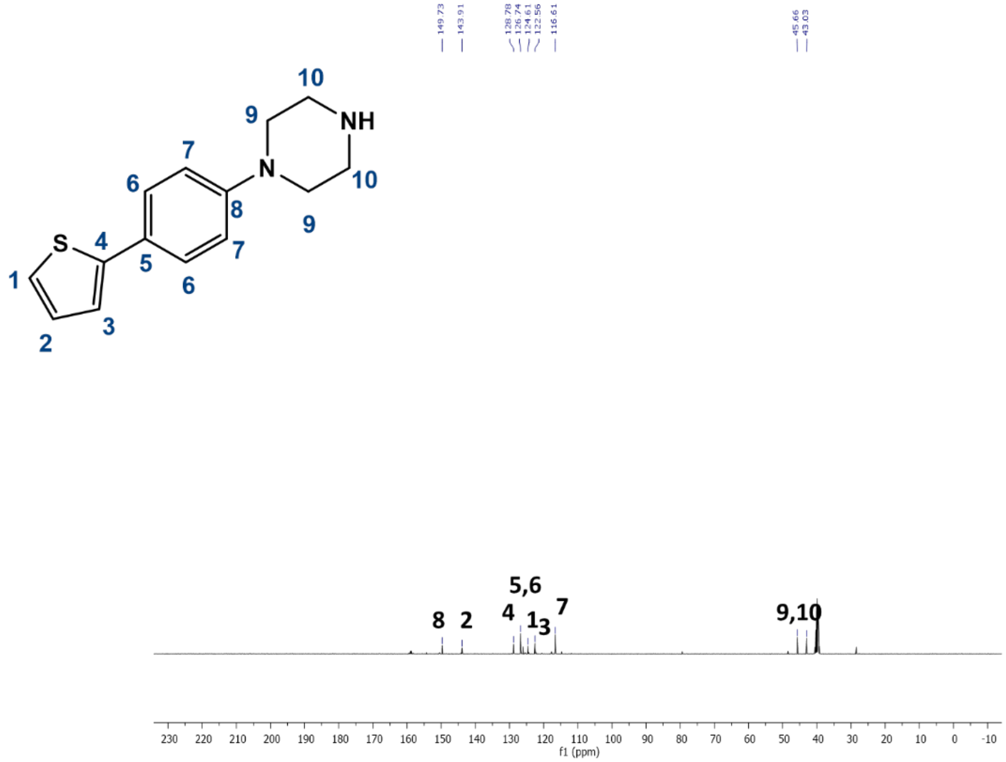

Figure S44: ^{13}C NMR spectrum of N-(4-thiophene-phenyl)piperazine (Entry 11b) in $CDCl_3$

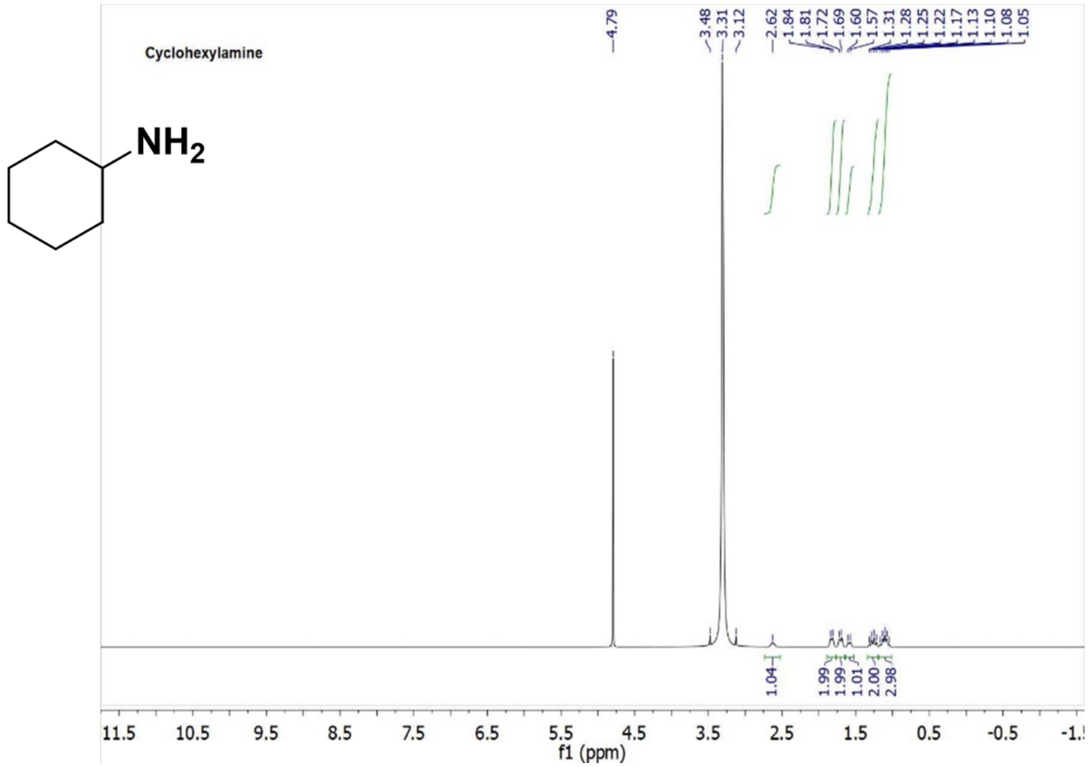

Figure S45: ^{1}H NMR spectrum of cyclohexylamine (Entry 12b) in $CDCl_3$

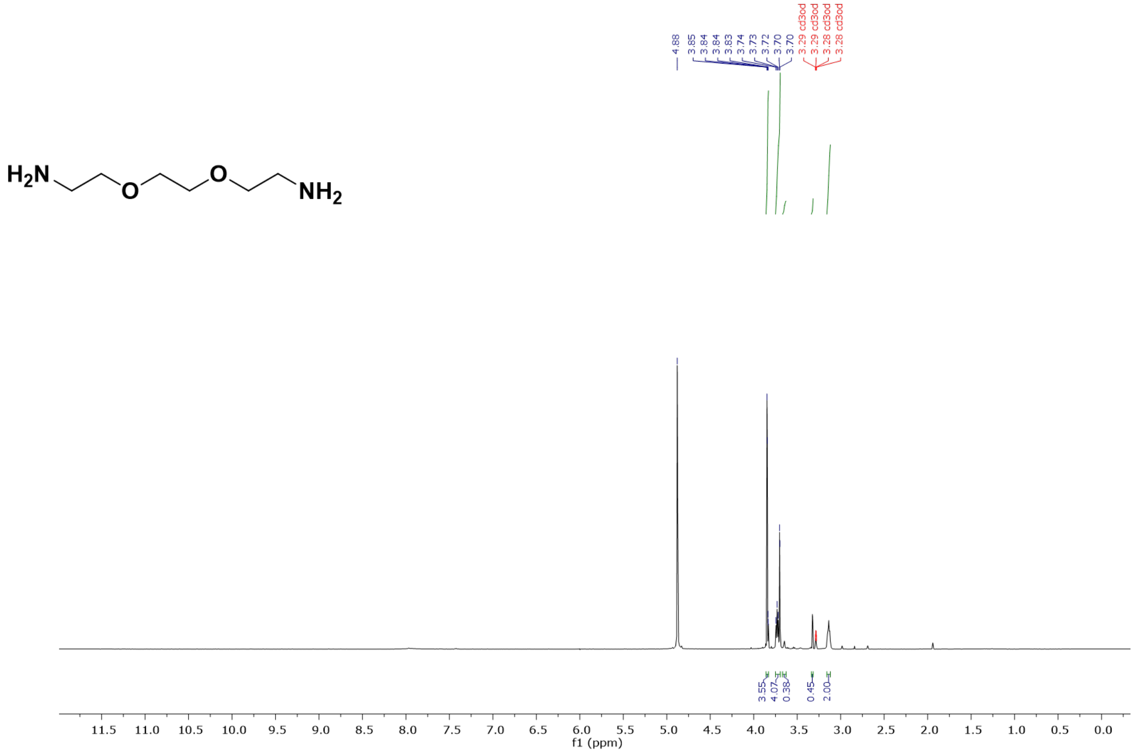

Figure S46: ^{1}H NMR spectrum of 2-[2-(2-aminoethoxy)ethoxy]ethanamine (Entry 13b) in $CDCl_3$

EC1 Deprotection Utilizing Oxalyl Chloride

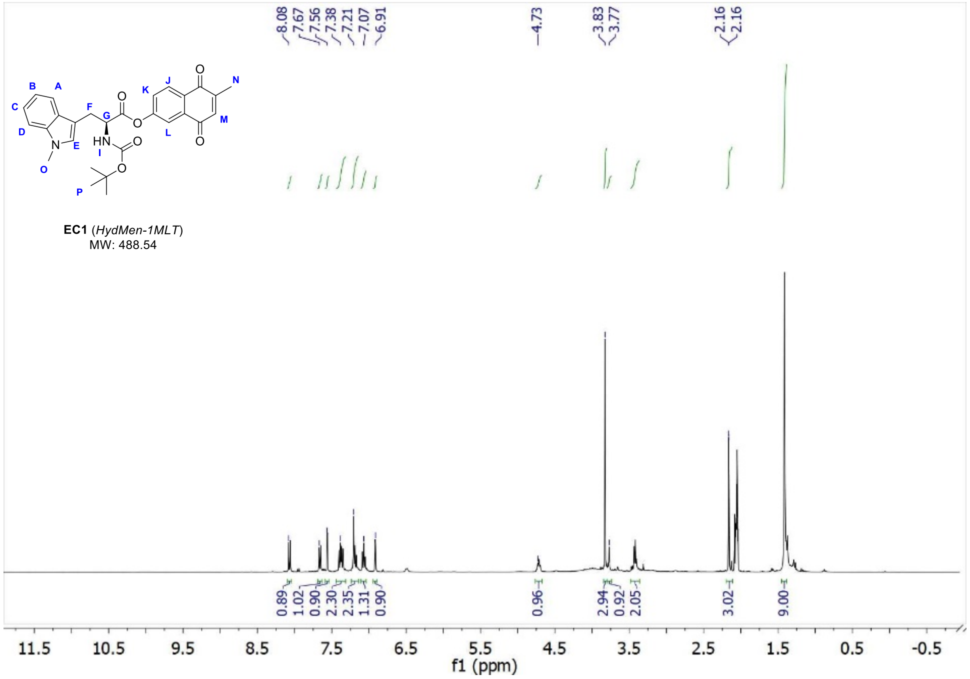

Figure S47: ^{1}H NMR spectrum of compound **4**

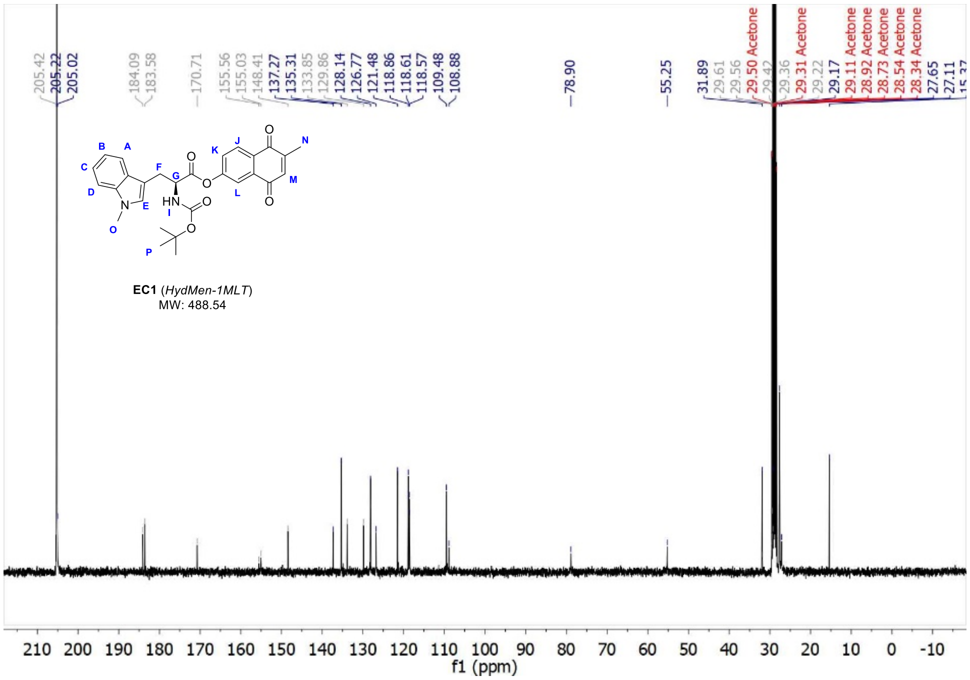

Figure S48: ^{13}C NMR spectrum of compound **4**

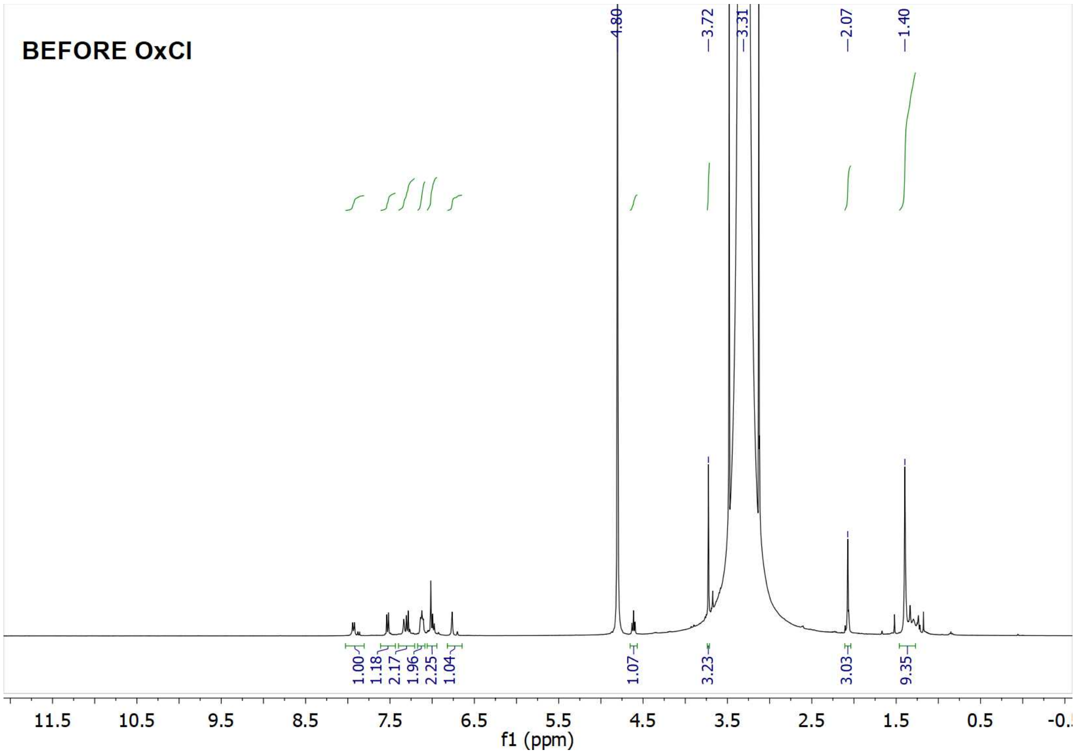

Figure S48: Reaction Monitoring via NMR (Before OxCl)

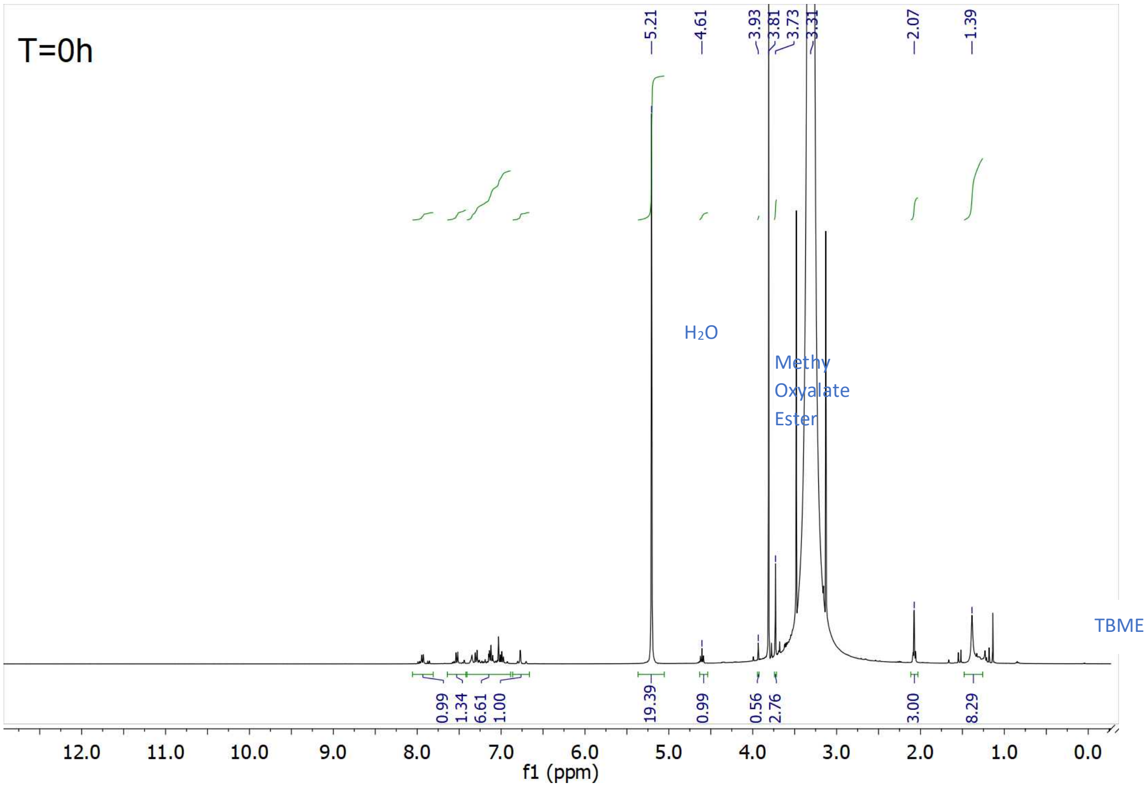

Figure S49: Reaction Monitoring via NMR (T = 0)

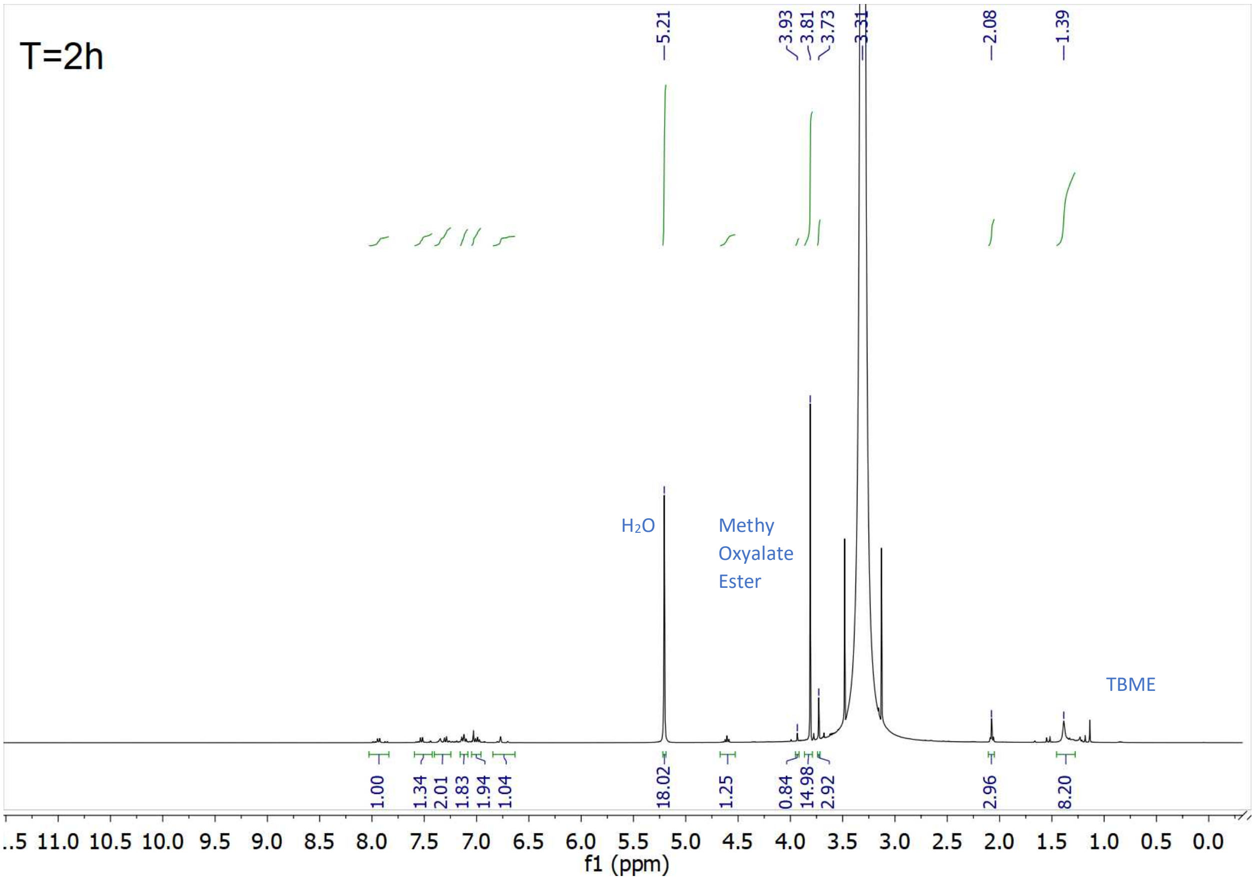

Figure S50: Reaction Monitoring via NMR (T = 2h)

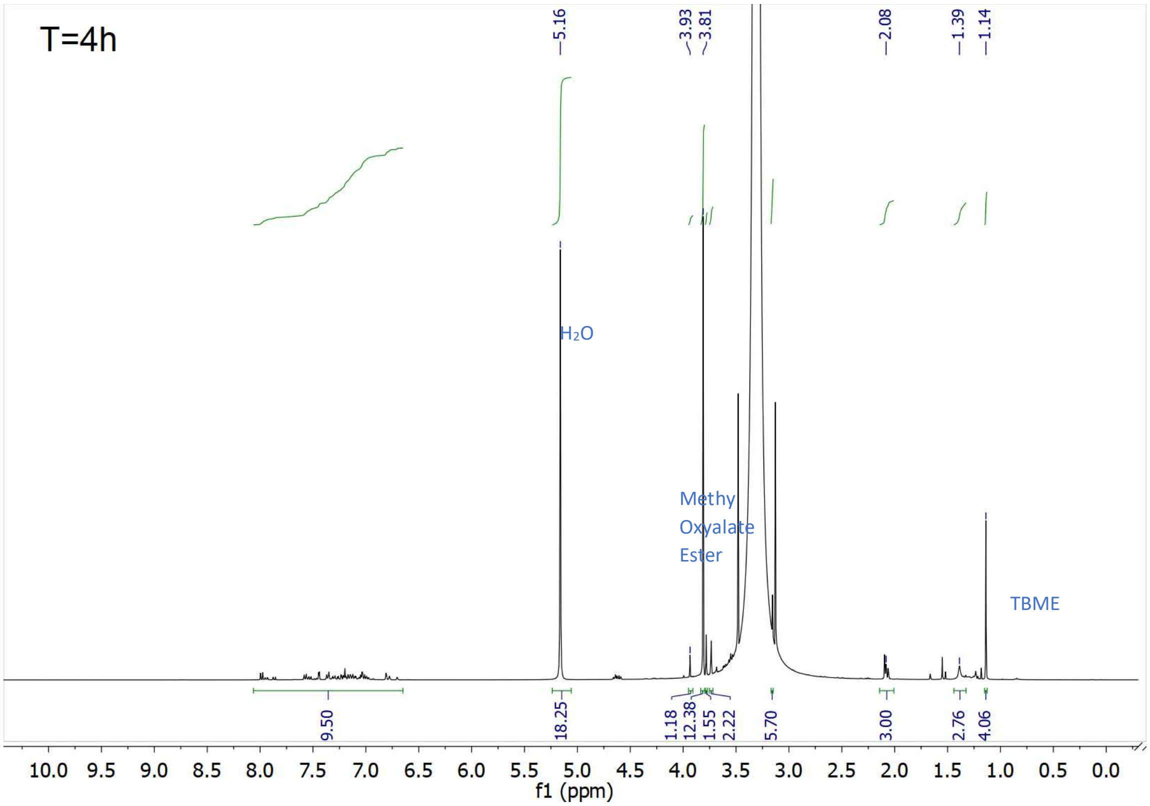

Figure S51: Reaction Monitoring via NMR (T = 4h)

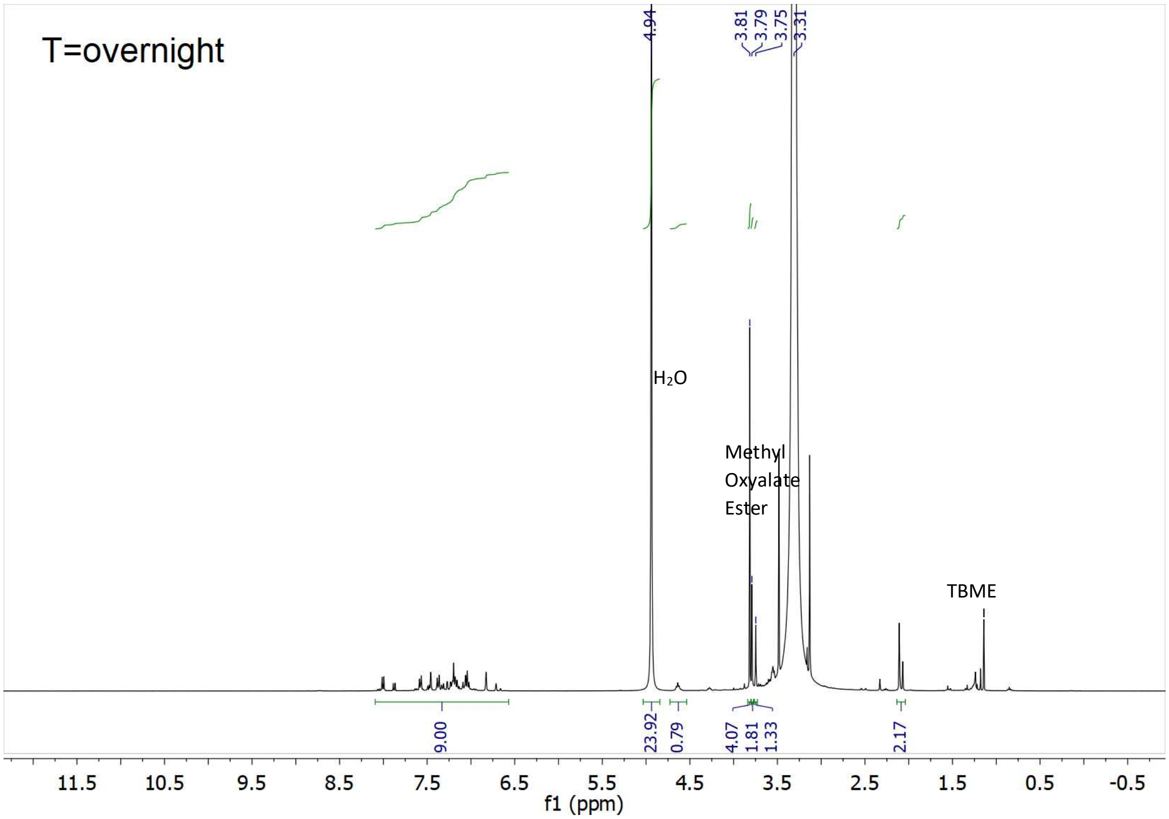

Figure S52: Reaction Monitoring via NMR (T = Overnight)

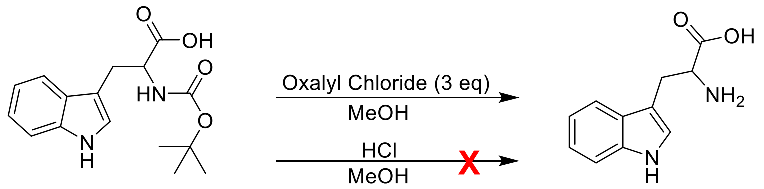

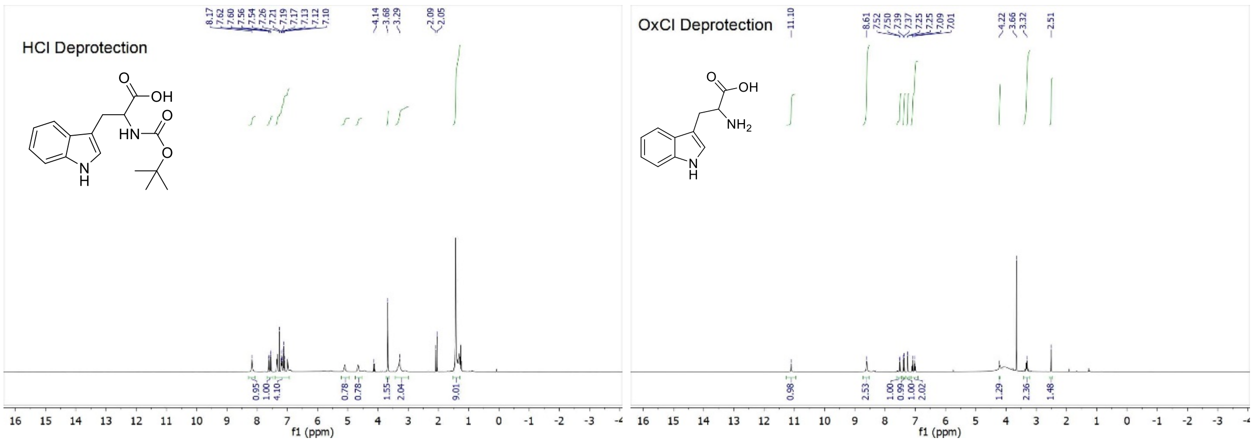

Figure S53: Determination of HCl effectiveness in Deprotection

X-ray Crystal Structure Data

Table S1: X-ray Parameters

X-ray Structural Data and Crystal Refinement	
	**EC1**
**Empirical Formula**	$C_{28}H_{28}N_2O_6$
**Molecular Weight (g/mol)**	458.54
**Temperature (K)**	90.0(2)
**X-ray Radiation (Å)**	CuKα (1.54178 Å)
**Crystal System, Space Group**	Monoclinic, C2
**Unit Cell Dimensions (Å, °)**	a = 23.791(2) Å alpha = 90<br>b = 6.7169 (6) Å beta =103.374(4)<br>c = 17.0032(16) Å gamma = 90
**Volume**	2643.5(4) $Å^3$
**Z**	2
**Absorption Coefficient**	1.663 mm^{-1}
**F(000)**	1116.0
**Crystal Size (mm)**	0.300 x 0.030 x 0.020
**Theta Range**	2.671 to 74.520

**Completeness to Theta = 67.679**	97.6 %
**F^2**	1.033
**Final R indices [I>2sigma(I)]**	R1 = 0.0380, wR2 = 0.0975

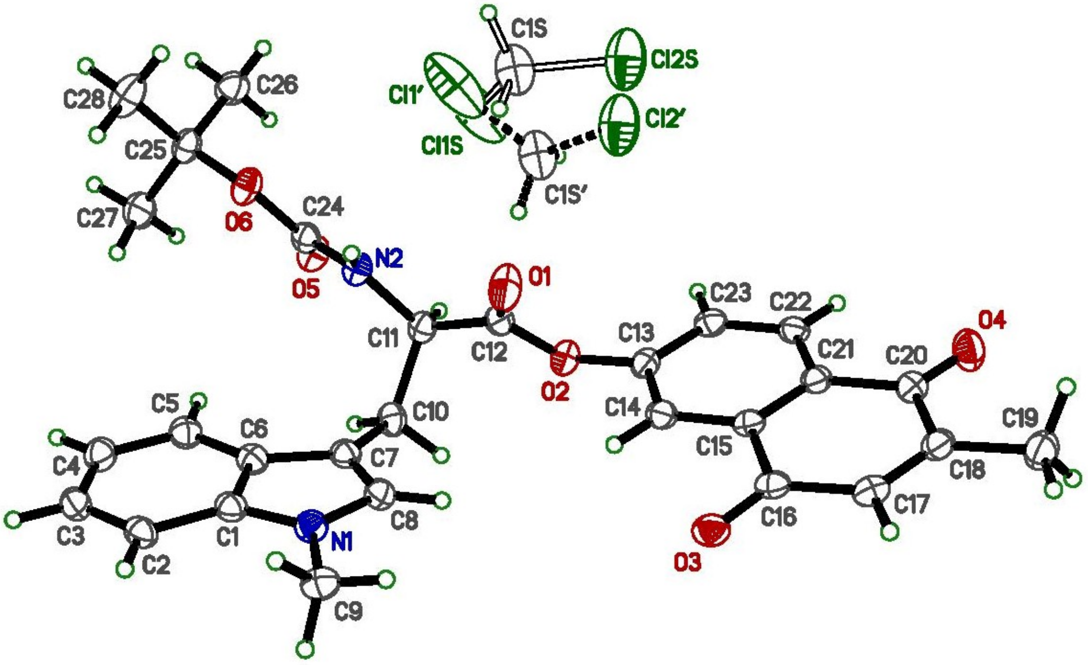

Figure S54: X-ray crystal structure of Compound **4** (EC1). X-ray structure crystalized with 2 molecules of dichloromethane per unit cell

Real time GC-MS spectra of oxalyl chloride deprotection

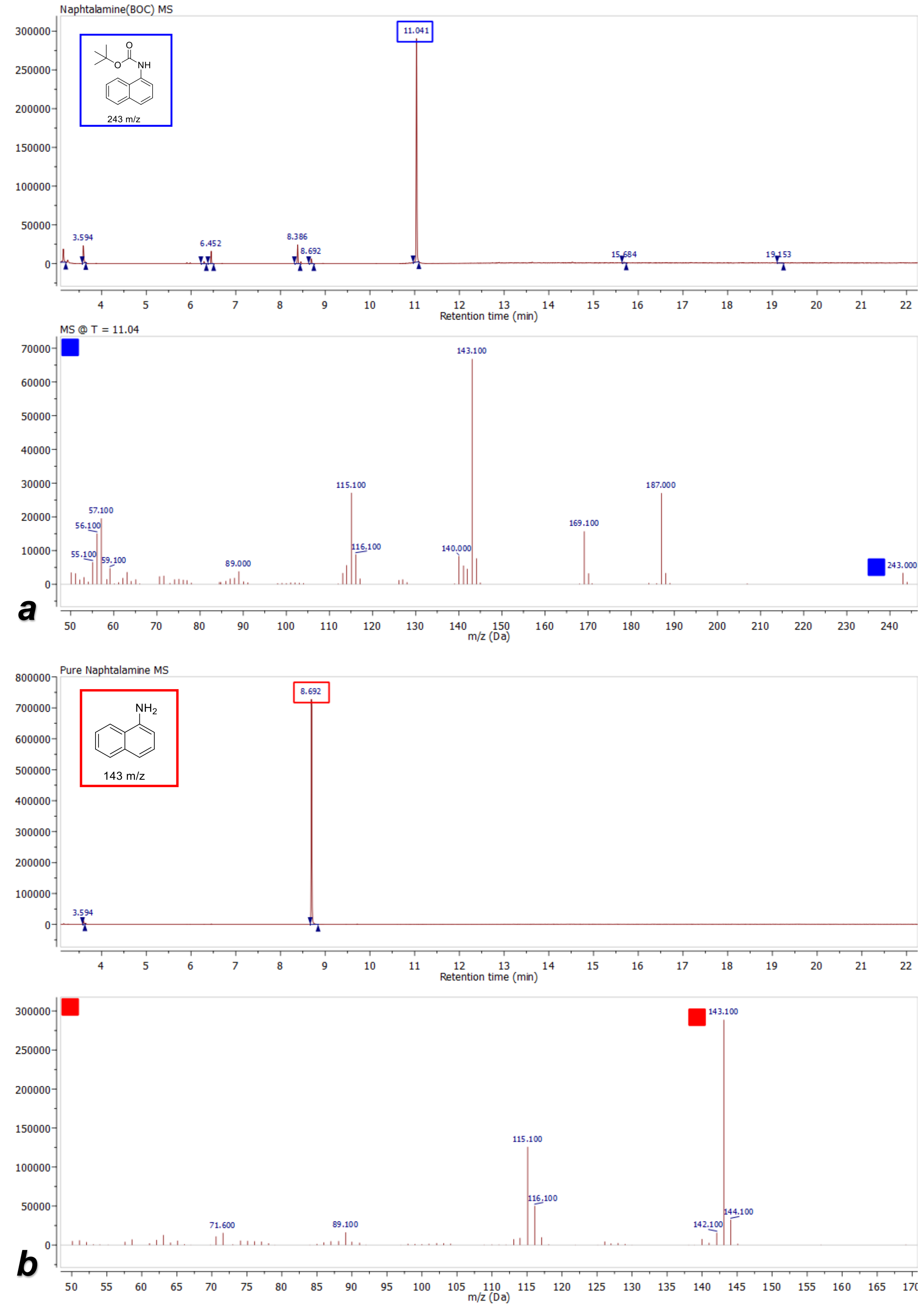

Figure S55*:* (***a***) GCMS of (N-BOC) Naphtalamine with rt = 11.041. (***b***) GCMS of Naphtalamine with rt = 8.69

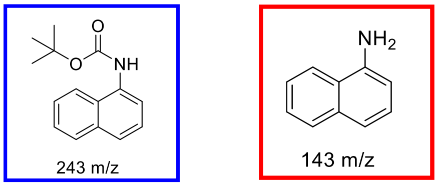

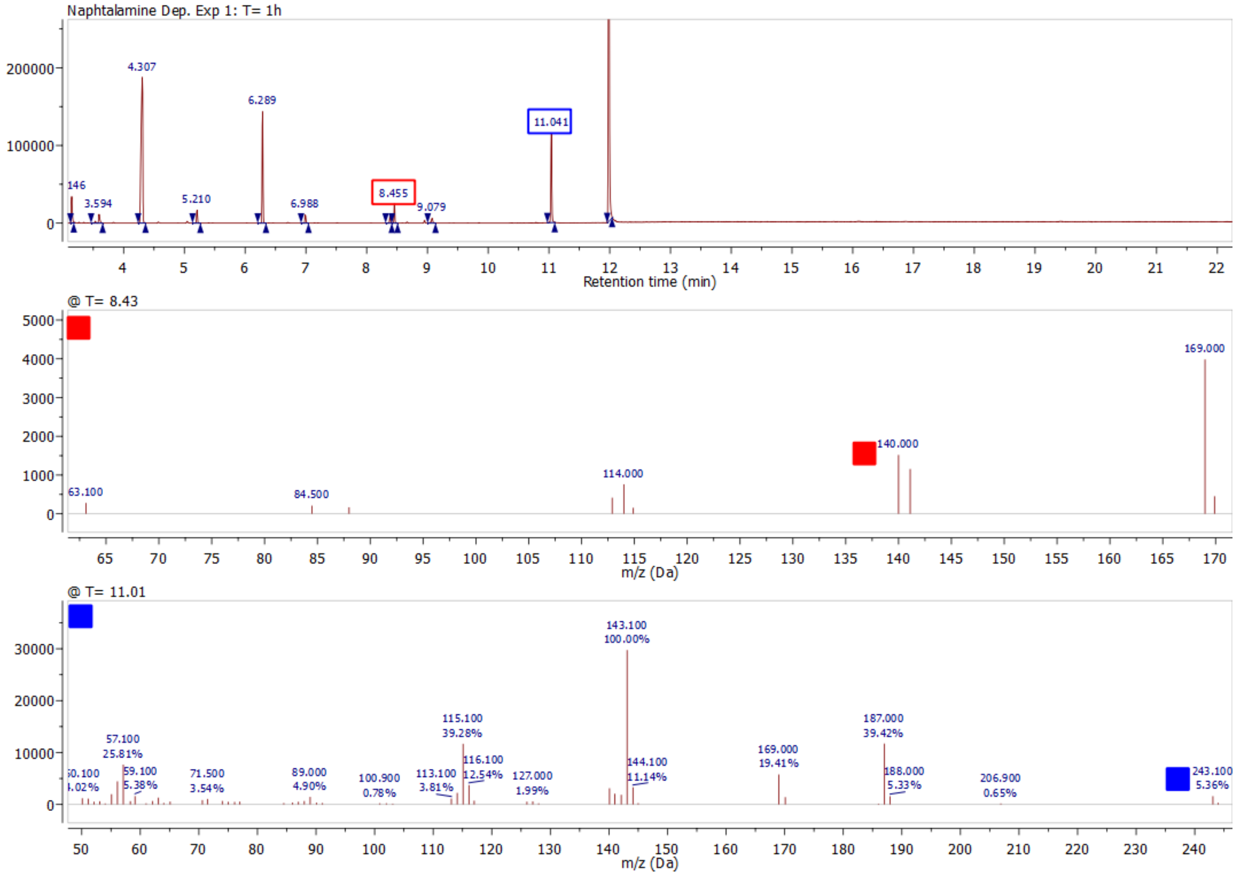

Figure S56*:* GCMS of (N-BOC) Naphtalamine deprotection reaction at time = 1 h. Boc protected starting material rt = 11.04. Intermediate rt = 8.455

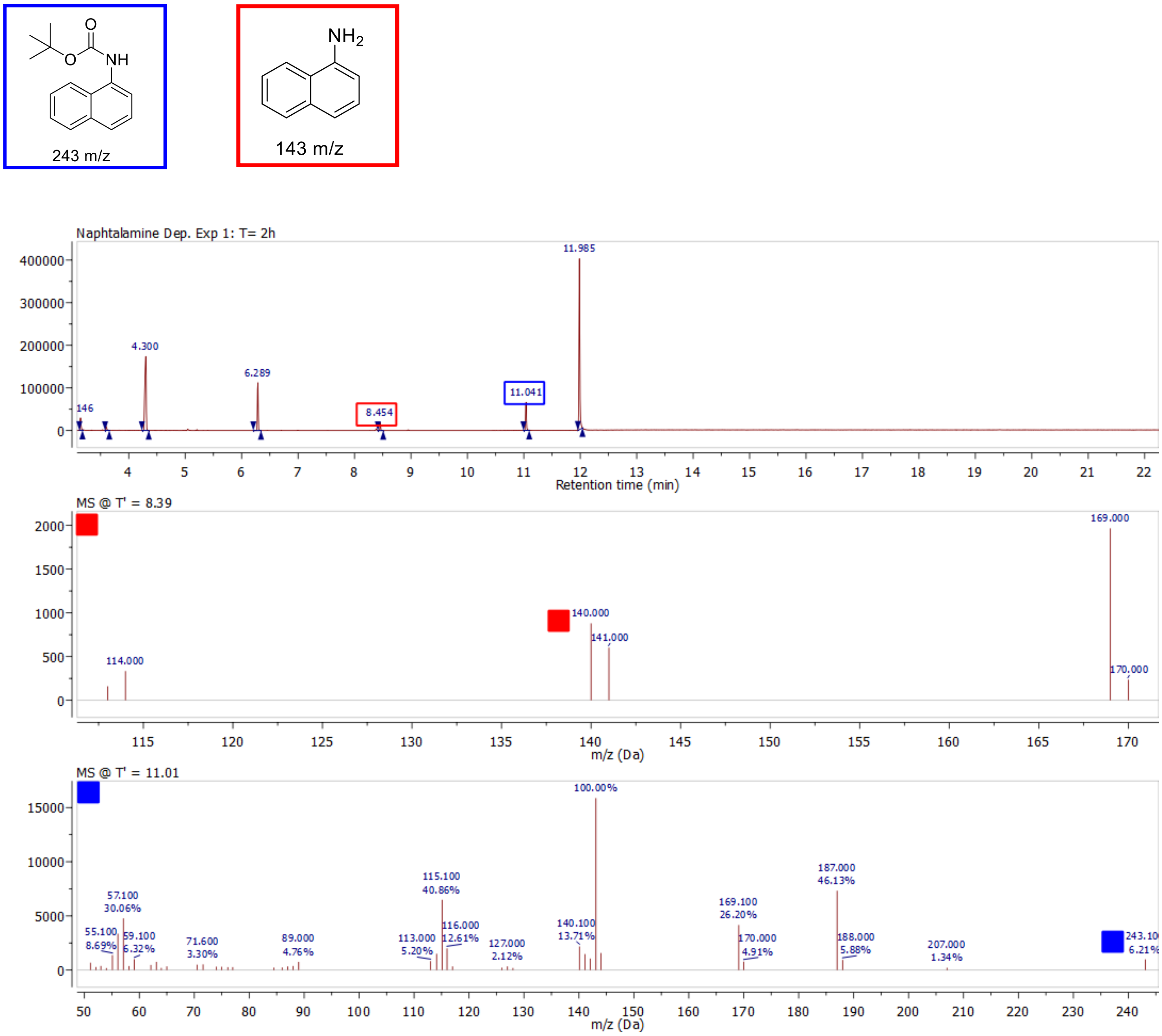

Figure S57: GCMS of (N-BOC) Naphtalamine deprotection reaction at time = 2 h. Boc protected starting material rt = 11.04. Intermediate rt = 8.455

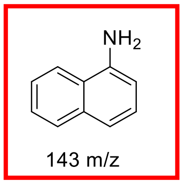

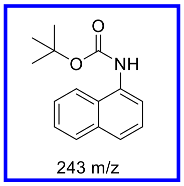

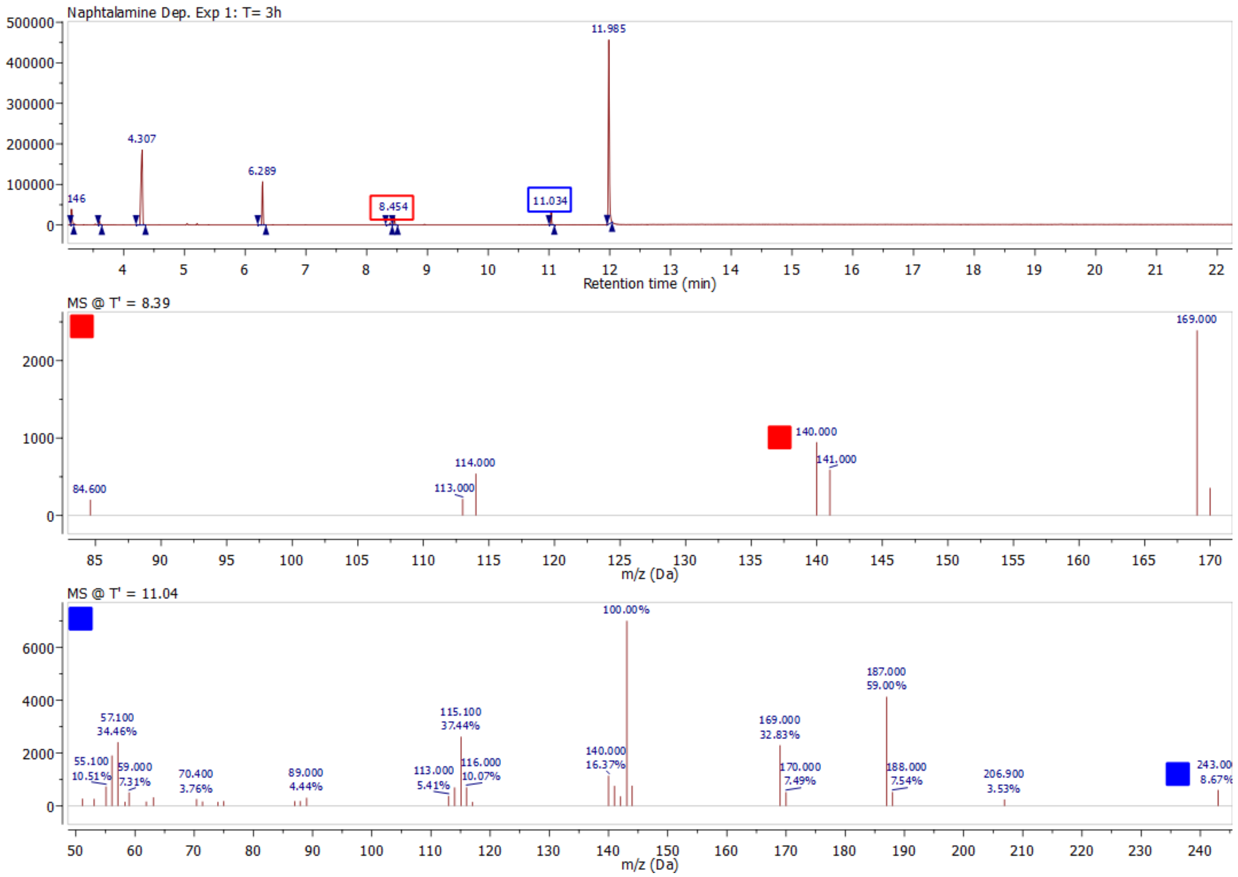

Figure S58: GCMS of (N-BOC) Naphtalamine deprotection reaction at time = 3 h. Boc protected starting material rt = 11.04. Intermediate rt = 8.455

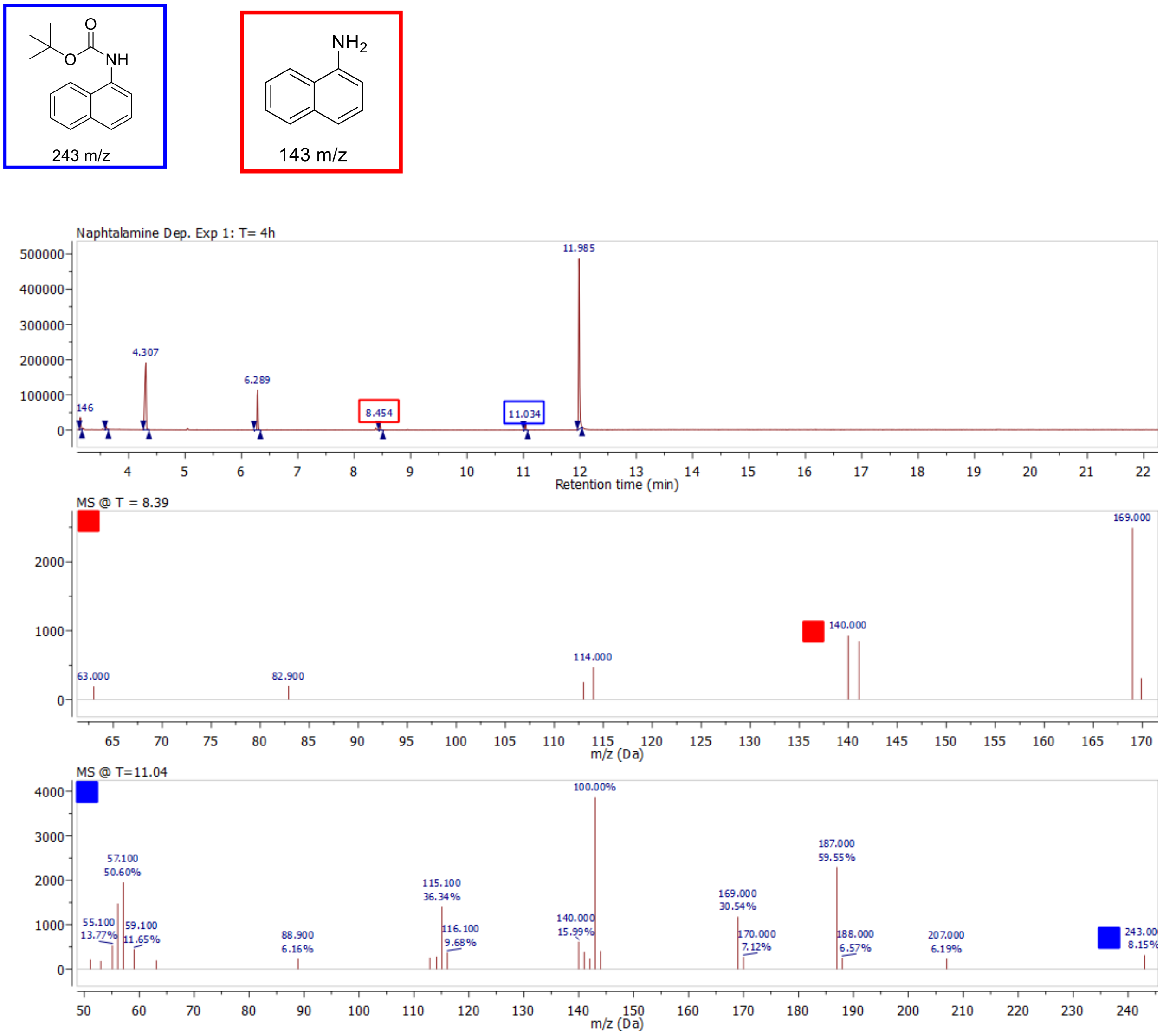

Figure S59: GCMS of (N-BOC) Naphtalamine deprotection reaction at time = 4 h. Boc protected starting material rt = 11.04. Intermediate rt = 8.455.

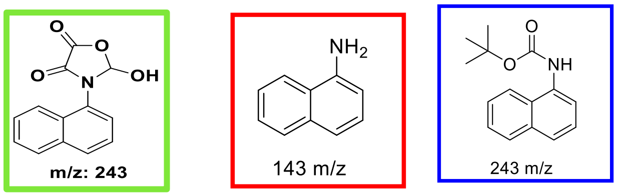

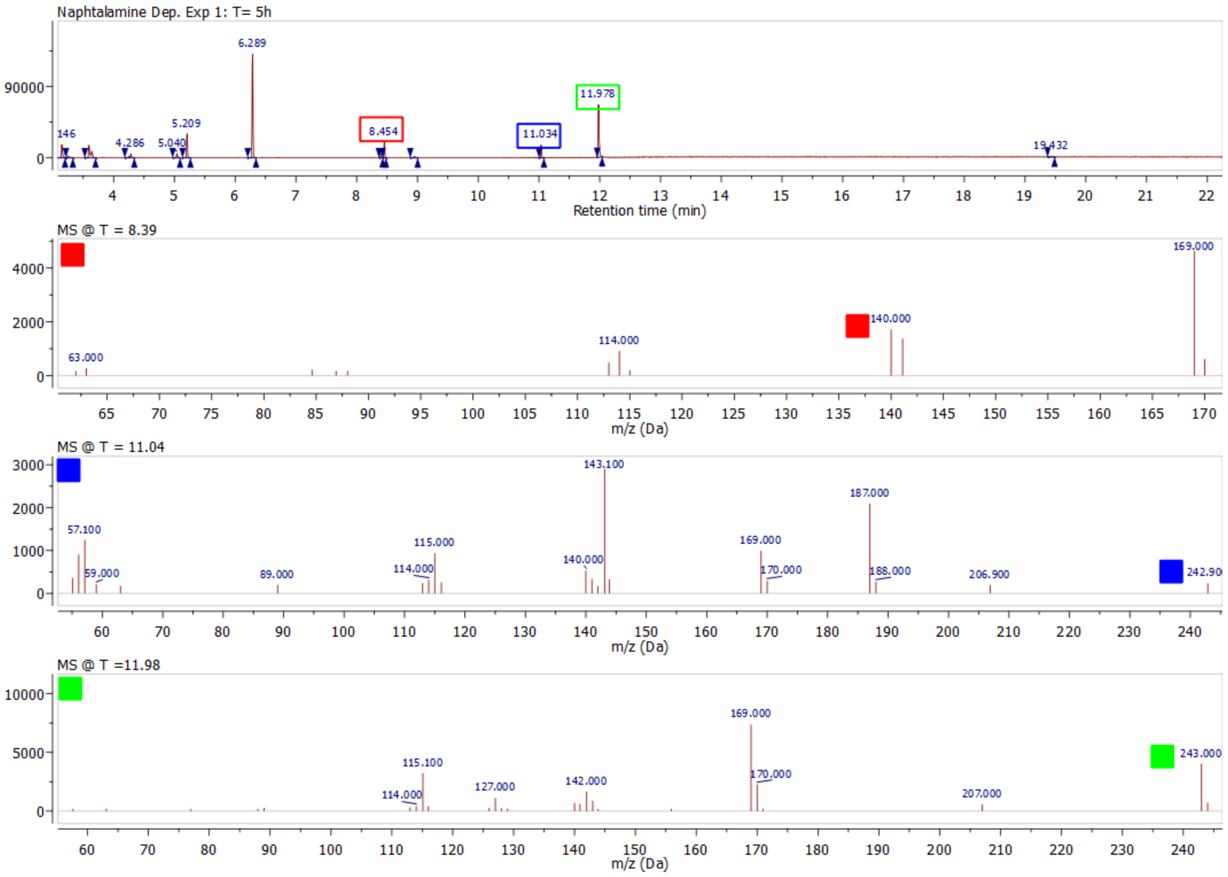

Figure S60: GCMS of (N-BOC) Naphtalamine deprotection reaction at time = 5 h. Boc protected starting material rt = 11.04. Intermediate rt = 8.455

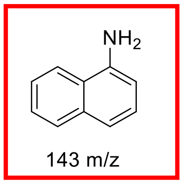

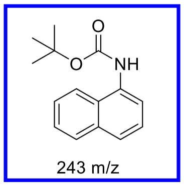

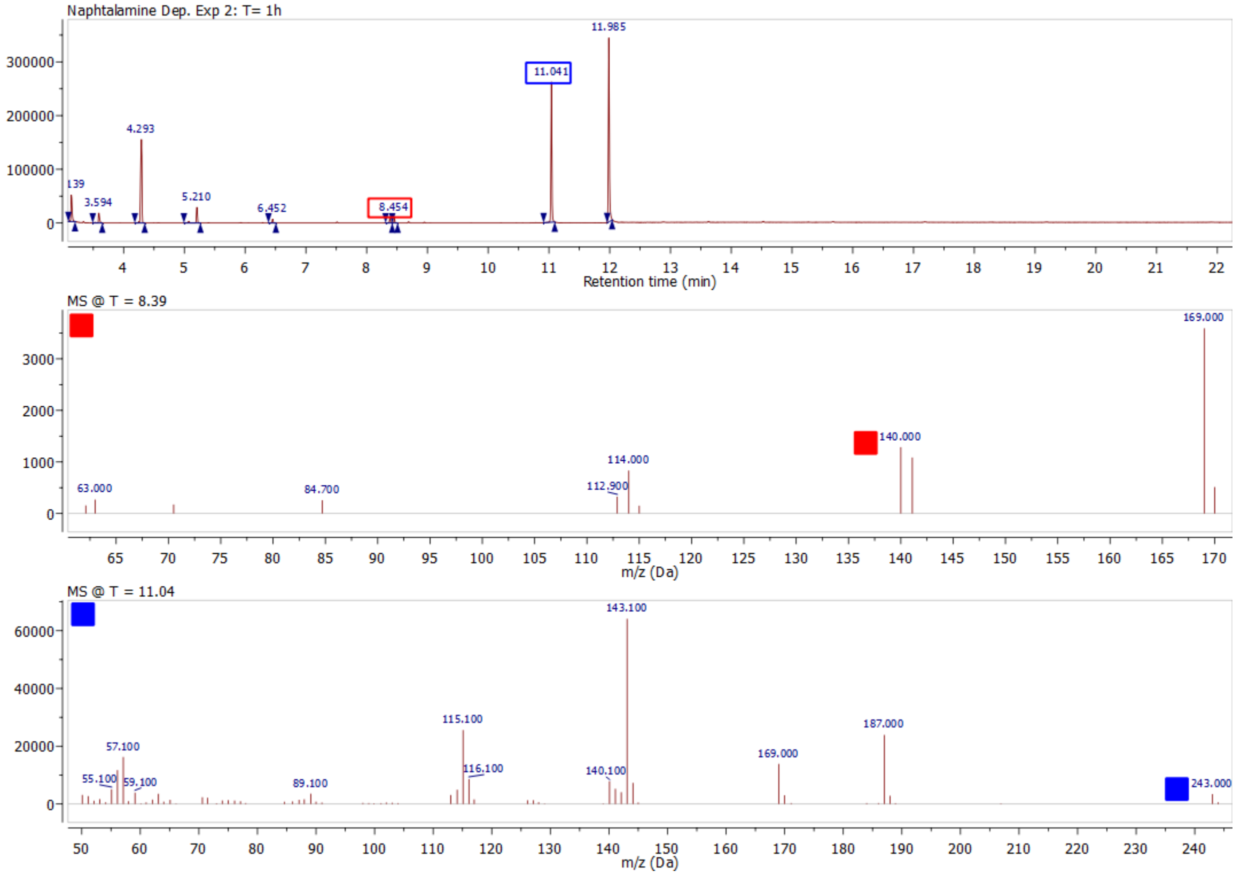

Figure S61: GCMS of (N-BOC) Naphtalamine deprotection reaction at time = 6 h. Boc protected starting material rt = 11.04. Intermediate rt = 8.455

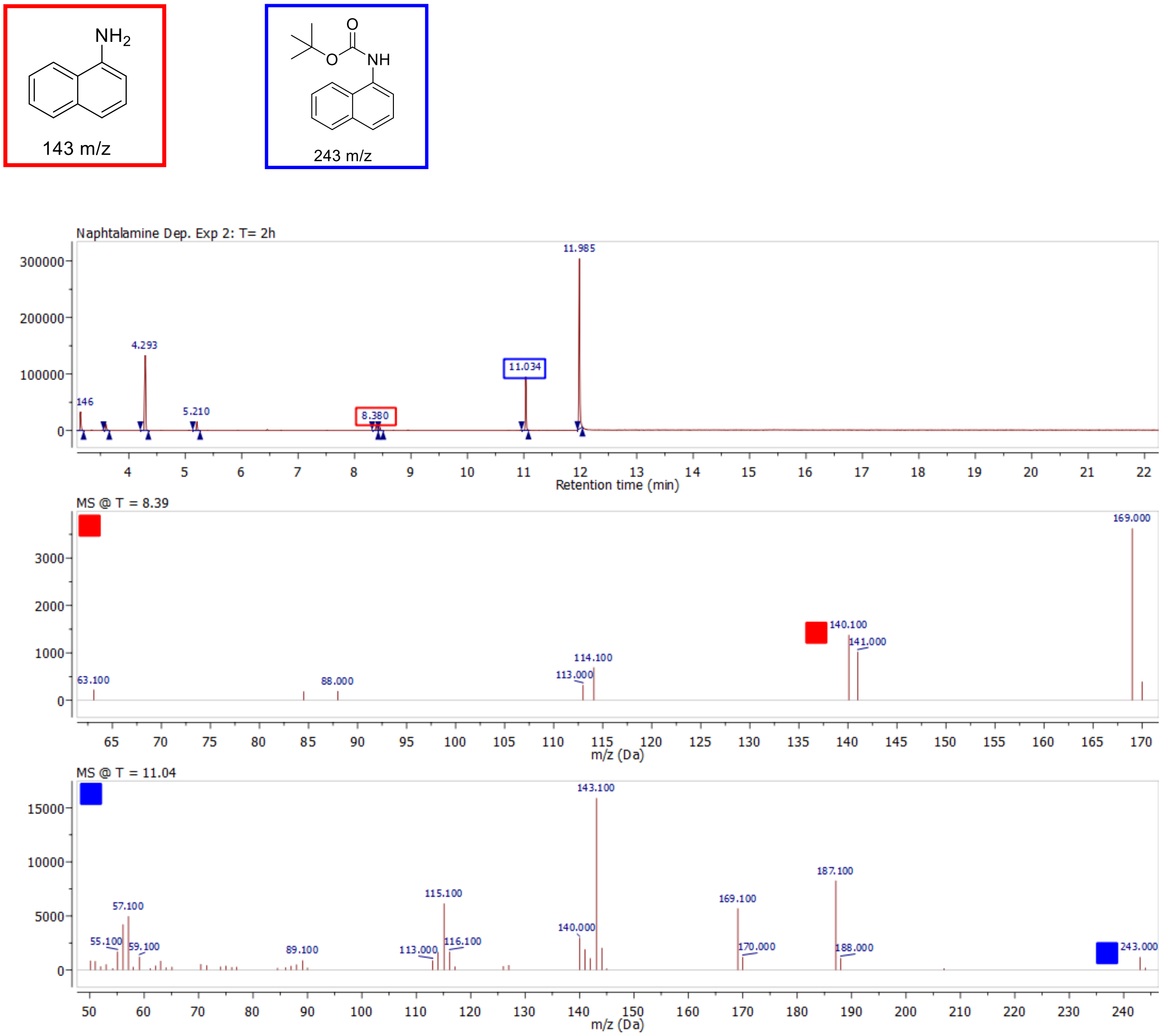

Figure S62: GCMS of (N-BOC) Naphtalamine deprotection reaction at time = 2 h. Boc protected starting material rt = 11.04. Intermediate rt = 8.455

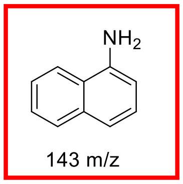

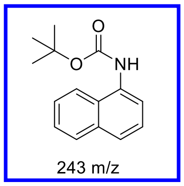

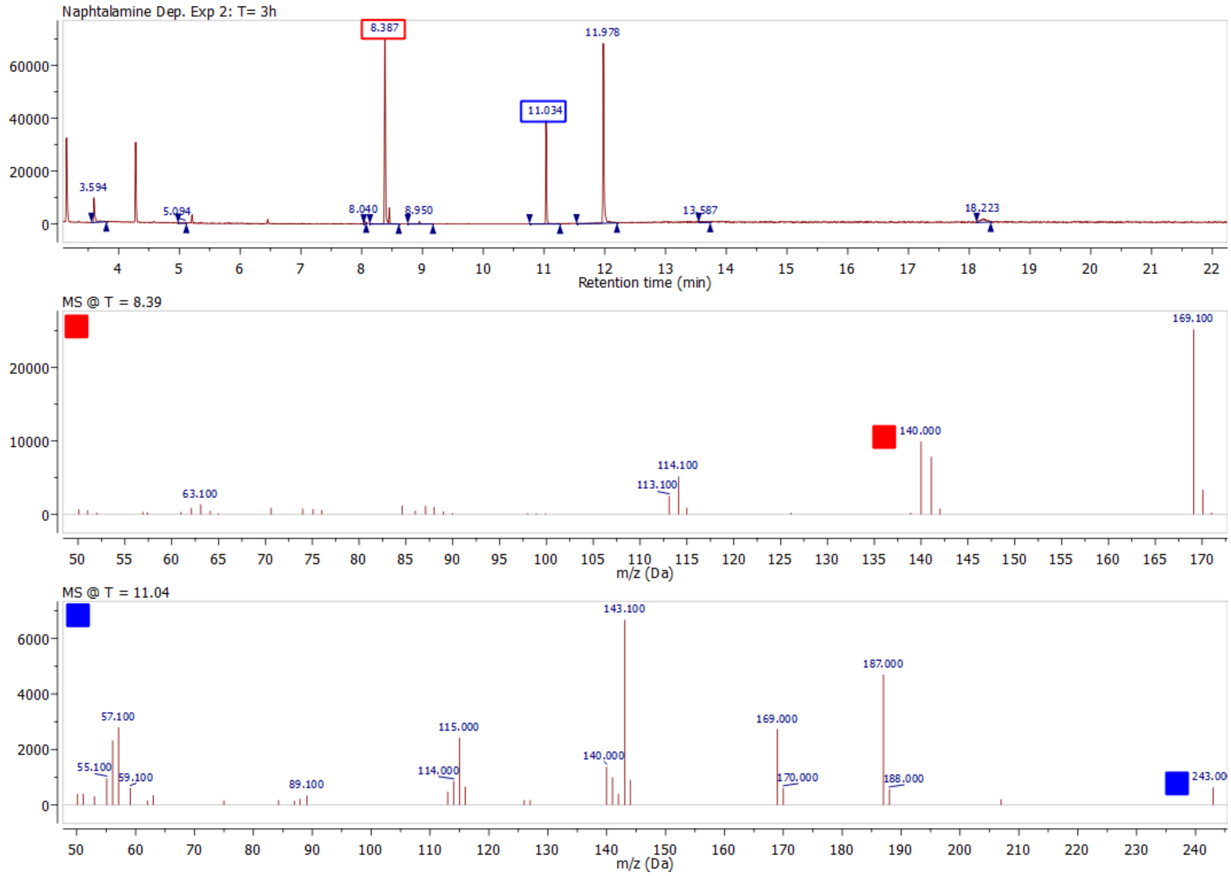

Figure S63: GCMS of (BOC) Naphtalamine deprotection reaction at time = 3 h. Boc protected starting material rt = 11.04. Intermediate rt = 8.455

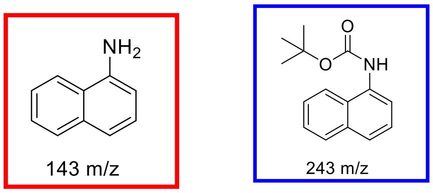

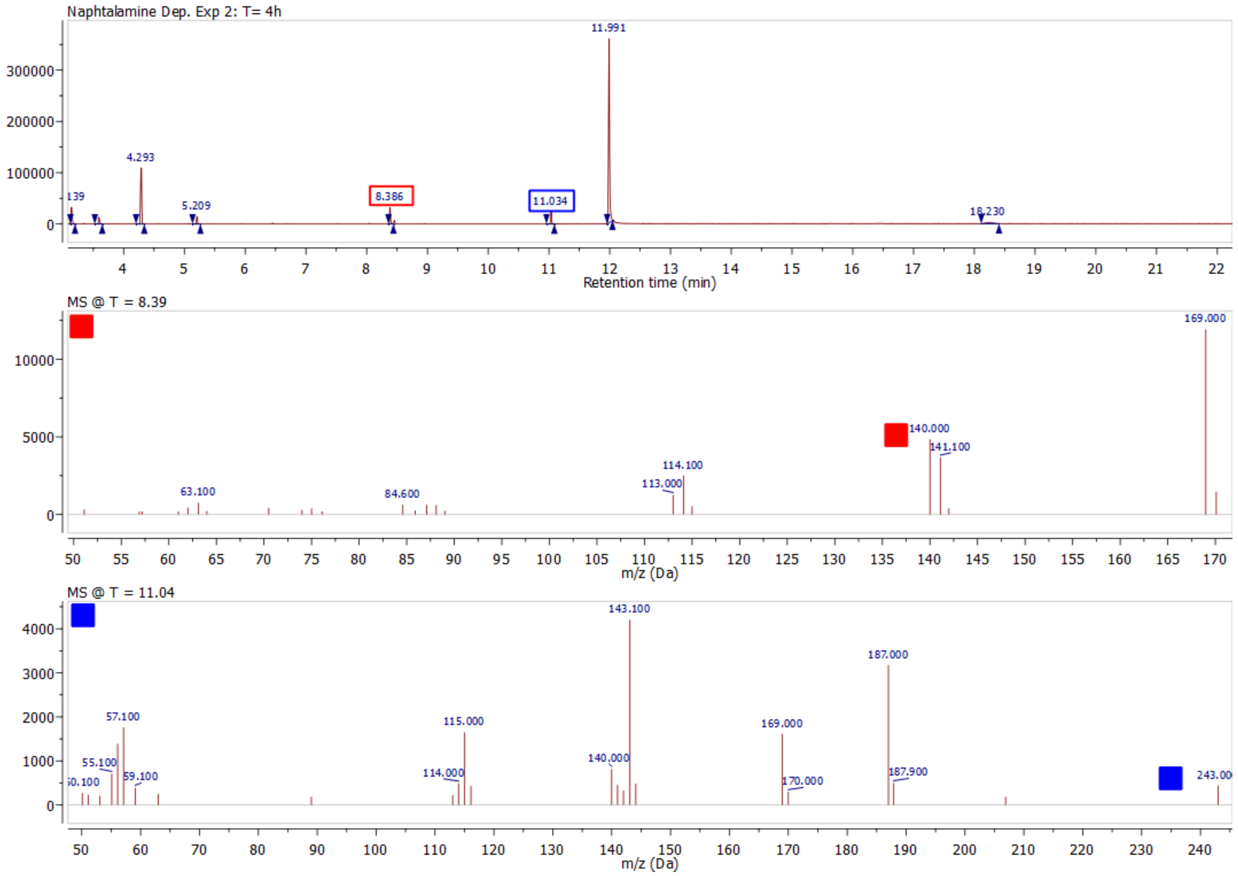

Figure S64: GCMS of (BOC) Naphtalamine deprotection reaction at time = 4 h. Boc protected starting material rt = 11.04. Intermediate rt = 8.455

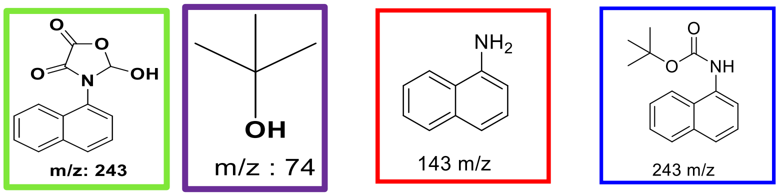

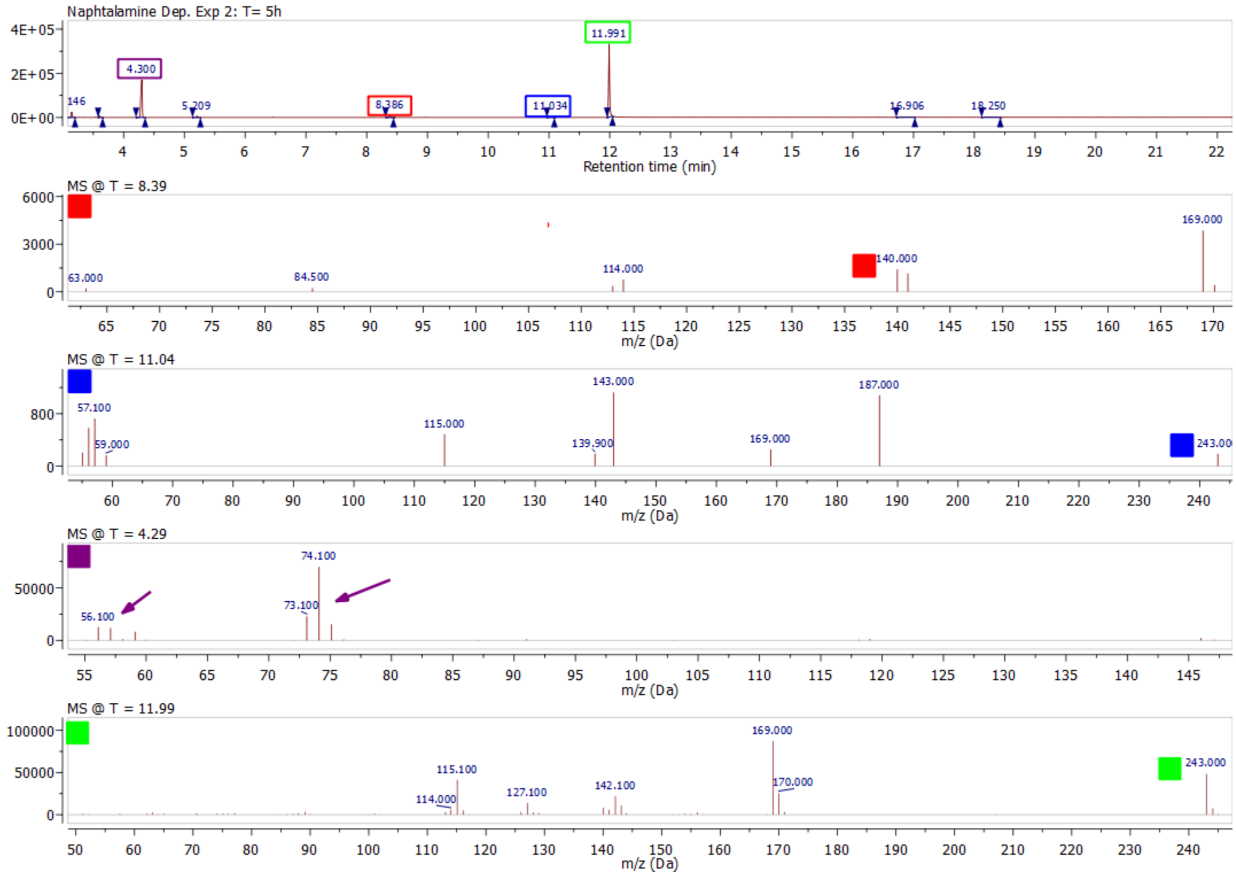

Figure S65: GCMS of (BOC) Naphtalamine deprotection reaction at time = 5 h. Boc protected starting material rt = 11.04. Intermediate rt = 8.455. Tert-butyl and t-butyl oxide ions rt = 4.3. Dichloro-intermediate located at rt= 11.991
